# Transcription Factor RBPJL Is Able to Repress Notch Target Gene Expression but Is Non-Responsive to Notch Activation

**DOI:** 10.3390/cancers13195027

**Published:** 2021-10-08

**Authors:** Leiling Pan, Philipp Hoffmeister, Aleksandra Turkiewicz, N. N. Duyen Huynh, Andreas Große-Berkenbusch, Uwe Knippschild, J. Christof M. Gebhardt, Bernd Baumann, Tilman Borggrefe, Franz Oswald

**Affiliations:** 1 Center for Internal Medicine, Department of Internal Medicine I, University Medical Center Ulm, Ulm University, Albert-Einstein-Allee 23, 89081 Ulm, Germany; leiling.pan@uni-ulm.de (L.P.); philipp.hoffmeister@uni-ulm.de (P.H.); 2 Institute of Biochemistry, University of Giessen, Friedrichstrasse 24, 35392 Giessen, Germany; turkiewicz.aleksandra.m@gmail.com; 3 Institute of Biophysics, Ulm University, Albert-Einstein-Allee 11, 89081 Ulm, Germany; nguyen.huynh@uni-ulm.de (N.N.D.H.); andreasgb@web.de (A.G.-B.); christof.gebhardt@uni-ulm.de (J.C.M.G.); 4 Department of General and Visceral Surgery, Surgery Center, Ulm University, Albert-Einstein-Allee 23, 89081 Ulm, Germany; uwe.knippschild@uniklinik-ulm.de; 5 Institute of Physiological Chemistry, Ulm University, Albert-Einstein-Allee 11, 89081 Ulm, Germany; bernd.baumann@uni-ulm.de

**Keywords:** Notch signaling, RBPJL, RBPJ, transcriptional repression, PDAC, Ptf1a, SHARP, AML

## Abstract

**Simple Summary:**

The transcription factor RBPJ is an integral part of the Notch signaling cascade. RBPJ can function as a coactivator when Notch signaling is activated but acts as a repressor in the absence of a Notch stimulus. Here, we characterized the function of RBPJL, a pancreas-specific paralog of RBPJ. Upon depletion of RBPJ using CRISPR/Cas9, we observed specific upregulation of Notch target gene expression. Reconstitution with RBPJL can compensate for the lack of RBPJ function in the repression of Notch target genes but is not able to mediate the Notch-dependent activation of gene expression. On the molecular level, we identified a limited capacity of RBPJL to interact with activated Notch1–4.

**Abstract:**

The Notch signaling pathway is an evolutionary conserved signal transduction cascade present in almost all tissues and is required for embryonic and postnatal development, as well as for stem cell maintenance, but it is also implicated in tumorigenesis including pancreatic cancer and leukemia. The transcription factor RBPJ forms a coactivator complex in the presence of a Notch signal, whereas it represses Notch target genes in the absence of a Notch stimulus. In the pancreas, a specific paralog of RBPJ, called RBPJL, is expressed and found as part of the heterotrimeric PTF1-complex. However, the function of RBPJL in Notch signaling remains elusive. Using molecular modeling, biochemical and functional assays, as well as single-molecule time-lapse imaging, we show that RBPJL and RBPJ, despite limited sequence homology, possess a high degree of structural similarity. RBPJL is specifically expressed in the exocrine pancreas, whereas it is mostly undetectable in pancreatic tumour cell lines. Importantly, RBPJL is not able to interact with Notch−1 to −4 and it does not support Notch-mediated transactivation. However, RBPJL can bind to canonical RBPJ DNA elements and shows migration dynamics comparable to that of RBPJ in the nuclei of living cells. Importantly, RBPJL is able to interact with SHARP/SPEN, the central corepressor of the Notch pathway. In line with this, RBPJL is able to fully reconstitute transcriptional repression at Notch target genes in cells lacking RBPJ. Together, RBPJL can act as an antagonist of RBPJ, which renders cells unresponsive to the activation of Notch.

## 1. Introduction

The highly conserved Notch signal transduction pathway controls numerous developmental decisions in embryonic and postnatal development and controls not only differentiation in several different organ systems but also stem cell maintenance and apoptosis. The pathway is highly sensitive to gene dosage; too little or too much signaling can promote oncogenesis. Notch1 itself is a proto-oncogene that is often found mutated in leukemia [1,2,3] and in breast cancer [4,5] Interestingly, in the context of skin cancer, Notch has been reported to have a tumour-suppressive function [6]. The activation of Notch signaling requires cell-to-cell contact and allows interaction between the Notch ligand on the signaling cell with the Notch receptor on the signal-receiving cell. Ligand-receptor interactions result in proteolytic cleavage of the Notch receptor and release of the Notch intracellular domain (NICD). Subsequently, NICD migrates into the nucleus, associates with the transcription factor RBPJ, assembles into a multifactorial coactivator complex and activates Notch target genes. RBPJ is also called CSL (CBF-1/Suppressor of Hairless/Lag-1), and is evolutionary conserved among *Homo sapiens*, *Drosophila melanogaster* and *C. elegans,* reviewed in [7]. In the absence of a Notch signal, RBPJ is still found at Notch target gene sites and represses the expression of Notch target genes. 

Notch target genes can be defined at promoters by (a) being bound by transcription factor RBPJ as measured by chromatin-immunoprecipitation [8,9,10], (b) the presence of a typical RBPJ binding motif GTGGGAA [11,12] and (c) transcriptional upregulation upon the induction of the activated form of Notch. Furthermore, Notch target genes can be downregulated upon the addition of gamma-secretase inhibitor (GSI) preventing the intracellular cleavage of the Notch receptor. Well-known Notch target genes include the proto-oncogene c-myc, as well as several members of the helix-loop-helix (bHLH) Hes- and Hey-transcription factor families [13,14] that again function as developmental master regulators. Interestingly, the Notch target genes, NRARP and Deltex, represent negative feedback regulators that make sure that the amplitude and duration of the Notch response is well controlled. 

The ubiquitously expressed transcription factor RBPJ is the central switch that can actively repress transcription in the absence of a Notch signal and support gene activation upon Notch activation. In the absence of a Notch signal, RBPJ remains bound at Notch target genes, recruits a SHARP/NCoR/HDAC-containing corepressor complex and actively represses transcription. Direct interactors of RBPJ have been described as SHARP/SPEN [15], KyoT2/FHL1 [16] and RITA [17]. SHARP/SPEN is able to recruit the NCoR/HDAC complex [18]. Previously, it was shown that several Notch target genes get derepressed upon depletion of RBPJ [19]. 

RBPJL is the only tissue-specific paralog of RBPJ, but its contribution in Notch signal transduction remains elusive. In the context of pancreas development, both RBPJ and RBPJL are able to form a heterotrimeric complex together with master regulator PTF1a and a common E-protein (bHLH) partner such as TCF12/HEB [20,21]. For the final differentiation step to the acinar lineage, RBPJL expression is strongly upregulated and ensures the effective transcription of acinar specific digestive enzymes, such as amylases, lipases and proteases, as components of the PTF1a complex. Apart from the activity of the Ptf1 complex, pancreas development also depends on canonical Notch signaling. The loss-of-function of one of the Notch components (*Notch1* and *Rbpj*) results in the depletion of epithelial precursors that in consequence do not promote acinar and islet cell formation and results in switching the cell fates into the early endocrine lineage [22,23]. In humans, missense mutations within the RBPJL gene have been detected in American Indians, resulting in the lower expression of RBPJL when compared to wildtype [24]. It is thought that RBPJL is important for keeping the acinar cell identity, since RBPJL-depleted cells start to express genes that are specific for the hepatic lineage [20]. 

In our study, we addressed the mechanisms of action of the pancreas-specific transcription factor RBPJL in comparison with its ubiquitously expressed paralog RBPJ. Both RBPJL and RBPJ bind to the same conserved octamer motif. Single-molecule experiments reveal that the binding times of both transcription factors within the nucleus of living cells are in the range of minutes. However, RBPJL shows slightly shorter binding times to chromatin suggesting a different composition of complexes. Indeed, RBPJL is unable to interact with the Notch1 intracellular domain (NICD) and other RAM-type binding partners like RBPJ. In addition, RBPJL does not support transactivation together with any of NICD1, -2, -3 or -4. However, both, RBPJL and RBPJ are able to interact with the corepressor SHARP. Importantly, we demonstrate that RBPJL can functionally compensate for the lack of RBPJ concerning the repression of endogenous Notch target genes. In summary, the RBPJ paralog RBPJL acts as a transcriptional repressor of Notch targets but is unable to respond to Notch-mediated transactivation.

## 2. Materials and Methods

### 2.1. Molecular Modeling of RBPJL

Homology modeling of mouse RBPJL was performed with swissmodel (https://swissmodel.expasy.org/, accessed on 2 April 2020). The crystal structure of mouse RBPJ/CSL bound to DNA (PDB entry 3BRG, [25]) was used for structural alignment. Modeling of human RBPJL was performed with swissmodel, alphafold2.0 [26] or robetta [27]. The crystal structure of human RBPJ/CSL (PDB entry 5EG6 [28]) was used for the structural alignment of human proteins. Figures were generated using PyMol (Molecular Graphics System, Version 2.0 Schrödinger, LLC). 

### 2.2. Cell Culture

The following cell lines were cultivated in Dulbecco’s modified eagle medium (DMEM^+/+^, Gibco, #41965-039) supplemented with 10% fetal calf serum (FCS) (Biochrom, #S0115), penicillin and streptomycin (Gibco, #15140-122): HEK293 (ATCC, CRL 1573), HeLa (ATCC, CCL 2), CRISPR-edited HeLa^RBPJ KO^ cells, AsPC-1 (ATCC, CRL-1682), PANC-1 (ATCC, CRL-1469), PA-TU-8902 (DSMZ, ACC 179), Capan-1 (ATCC, HTB-79), Panc-215 (kindly provided by P. Hermann, Ulm, Germany), MIA PaCa-2 (ATCC, CRL-1420), DAN-G (CLS, #300162) and HCT-116 (colorectal carcinoma, ATCC, CCL-247). Cell lines U-937 (histiocytic lymphoma, DSMZ, ACC 5), NB-4 (acute promyelocytic leukemia, DSMZ, ACC 207) and THP-1 (acute monocytic leukemia, DSMZ, ACC 16) were grown in RPMI-1640 medium (Gibco, #21875-034) supplemented with 10% FCS, penicillin and streptomycin. 

### 2.3. Retroviral Transduction of CRISPR/Cas9 RBPJ-Depleted Hela Cells for the Stable Expression of EGFP-Tagged RBPJL

HEK 293T cells (2.5 × 10^6^) were seeded in a 10 cm plate with 10 mL of DMEM^+/+^ medium and incubated at 37 °C and 5% CO_2_ for 24 h. Afterwards, 100 µL of DMEM^+/+^ medium, 1.5 µg of pVSV-G, 1.5 µg of pGAG-Pol and 7.0 µg of retroviral vector (see Table S1) were mixed and incubated for 20 min at room temperature (RT). Separately 30 µL of Lipofectamine 2000 transfection reagent (Invitrogen, #11668019) were added to 900 µL of DMEM^+/+^ medium. Both solutions were collected and incubated for 20 min at RT. Thereafter, the transfection mix was added to the HEK 293T cells and incubated at 37 °C and 5% CO_2_ for 48 h. Next, the viral supernatant was filtered (10 mL syringe and 0.45 micron filter), supplemented with 2 µg/mL of polybrene and used for the infection of HeLa^RBPJ KO^ cells seeded the day before (0.7 × 10^6^ per 1 well of a 6-well plate). In order to obtain fresh viral supernatant, HEK 293T cells were incubated with fresh DMEM^+/+^ medium for the next 24 h. HeLa^RBPJ KO^ cells were spinoculated with 5 mL of the resulting viral supernatant at 1800 rpm for 45 min. Afterwards, the supernatant was exchanged with the DMEM^+/+^ medium. The spinning procedure was repeated with fresh viral supernatant on the next day. After 48 h, cells were subjected to blasticidin (Gibco, #R21001) selection medium (2.5 µg/mL), expanded and collected for Western blotting and gene expression analysis. 

### 2.4. RNA Extraction and qRT-PCR

Tissues and cells were homogenized by QIAshredder (Qiagen, #79656) or lysed with TRIzol reagent (Ambion, #15596018), respectively. Total RNA was purified using the RNeasy Mini Kit (Qiagen, #74106) and the DNase I (Qiagen, #79254) accordingly to manufacturer′s instructions. RNA concentration was determined by the use of a NanoDrop 2000 (PeqLab Biotechnology). To reverse-transcribe RNA to cDNA, 1 μg RNA, 1 µL random primers (100 ng/µL), 1 µL dNTP-Mix and DEPC-treated water (in total 13 µL) were incubated for 5 min at 65 °C. Afterwards, 4 µL 5× First strand buffer, 2 µL 50 mM DTT and 1 µL SuperScript II reverse transcriptase (Invitrogen, #18064-014) were applied to the mixture and incubated for 1 h at 42 °C, followed by a heat inactivation step at 70 °C for 15 min. QuantiTect SYBR Green PCR kit (Qiagen, #204056) was used for the qPCR reaction in a Light Cycler 480 Real-Time PCR system (Roche) device. The expression of the genes of interest was normalized to the expression of the housekeeping gene *HPR*T1. The qRT-PCR assays used in this study are given in Table S1. 

### 2.5. Analysis of Single Cell RNAseq Data Set

The human pancreas scRNAseq data set (GSE81547 [29]) was reanalyzed as described in [30].

### 2.6. Mice

Mice were bred and housed in specific pathogen-free conditions in accordance with institutional, state and federal guidelines on animal welfare. All animal experiments were carried out in cooperation with the animal facility at the University of Ulm in accordance with the German animal protection law “Tierschutzgesetz” §8, Abs. 1 and 3. 

### 2.7. Tumor Tissue Samples

Tumor tissue and normal pancreatic tissue from 9 pancreatic ductal adenocarcinoma (PDAC) patients, whose informed consent was obtained prior to surgery, was drawn from the tissue bank of the Department of General and Visceral Surgery of the University Hospital Ulm. Tissue samples were collected during operation, and specimens were subjected to routine pathological analysis and defined as “PDAC” or “normal”. Sample collection was performed with the permission of the independent local ethics committee of the University of Ulm (approval 235/15).

### 2.8. Isolation of Primary Pancreatic Acinar Cells and ADM Assay

In order to further analyze the acinar cells in vitro, the pancreas was directly taken out from a C57BL/6 mouse and rinsed twice in ice cold HBSS (Corning, #21-021-CV) and centrifuged at 1000 rpm for 3 min at 4 °C. The pancreas was sliced into 1–5 mm pieces, and digested with 10 mL collagenaseP (2 mg) (Roche, #11213857001) solution for 20–30 min in the 37 °C incubator. Mechanical dissociation was performed by up and down pipetting of the cells (10 mL pipette) every 5 min. To stop the digestion, a 10 mL ice-cold washing solution [HBSS with 5% FCS (boiled at 56 °C for 50 min before use) and 10 mM HEPES (Gibco, #15630-056)] was applied. The whole mixture was centrifuged at 1000 rpm for 2 min at 4 °C. After washing twice using the washing solution, the mixture was filtered through a 100 µm cell strainer (Corning, #431752). Afterwards, the cell suspension was added dropwise on top of a 2 mL HBSS solution with 30% FCS. After centrifugation at 1000 rpm for 2 min at 4 °C, the acini were washed using 10 mL Waymouth’s medium (Gibco, #31220-023) [after adding 1% FCS and 0.1 mg/mL trypsin inhibitor (Merck, #T9003) and 1 μg/mL dexamethasone (Merck, #D2915)]. The acinar cells were mixed with Waymouth’s medium and growth factor reduced Matrigel (diluted 1:1.5) (Corning, #354230) and were seeded in a 24-well plate. Each well was incubated with 400 µL of the cell-gel mixture for 30 min at 37 °C. Subsequently, 600 µL of Waymouth’s medium was applied to each well. TGFα (500 ng/well) (Merck, #T7924) was added and used as positive control. The images were acquired using a Leica LEITZ DM-IRBE microscope and processed by QCapture Suite PLUS software (QImaging).

### 2.9. DNA Transfection

HEK293 and HeLa cells were transfected using the Calcium-Phosphate protocol (Promega, #E1200) or Lipofectamine 2000 transfection reagent (Invitrogen, #11668019), according to the manufacturer’s instructions. 

### 2.10. Protein Fractionation

In order to obtain nuclear extract (NE), protein fractionation was prepared as follows: 1 × 10^7^ cells were pelleted, washed in 10 mL of PBS, transferred to a 1.5 mL reaction tube and pelleted. The pellet was resuspended in 200 µL of freshly prepared extraction buffer A (10 mM Hepes pH 7.9, 10 mM KCl, 0.1 mM EDTA, 0.1 mM EGTA, 1 mM β-mercaptoethanol and 2 μL PMSF), incubated on ice, mixed with 5 µL of 10% NP-40 and centrifuged at 13,000 rpm for 10 s at 4 °C. Afterwards, the pellet was resuspended in 100 µL of freshly prepared extraction buffer C (20 mM Hepes pH 7.9, 400 mM NaCl, 1 mM EDTA, 1 mM EGTA 1 mM 1 mM β-mercaptoethanol and 2 μL PMSF), incubated on ice for 20 min and agitated every 4 min during the incubation time. The extraction mix was centrifuged at 13,000 rpm for 10 min at 4 °C. The resulting supernatant was transferred to a new 1.5 mL reaction tube for subsequent protein concentration measurement using the Bradford assay (BioRad, #5000006). Samples were afterwards subjected to Western blot analysis.

### 2.11. Co-Immunoprecipitation Experiments

Cells (HEK293) were transfected with the indicated constructs for the expression of GFP- and Flag-tagged proteins. Then, 24 h after transfection, cells were lysed in 600 μL CHAPS lysis buffer [10 mM 3-[(3-cholamidopropyl)-dimethylammonio]-1-propanesulfonate hydrate (CHAPS, Merck, #C3023), 50 mM Tris-HCl (pH7.8), 150 mM NaCl, 5 mM NaF, 0.5 mM phenylmethanesulfonyl fluoride (PMSF) (Merck, #P-7626) and 40 μL/mL cOmplete protease inhibitor cocktail (Roche, #13539320)]. Extracts were incubated with agarose-conjugated anti-Flag antibody (M2, Merck, #A2220) at 4 °C overnight. After washing (6 to 8 times with CHAPS lysis buffer), the precipitates were resuspended in 1× SDS-polyacrylamide gel loading buffer. The plasmids used in this study are given in Table S1. 

### 2.12. Western Blotting

Samples were mixed with 6× SDS loading dye and boiled for 5 min at 95 °C. All samples were applied to SDS-polyacrylamide gels and transferred electrophoretically at RT to PVDF membranes (Millipore, #IPVH00010) for 1 h at 250 mA using a Tris-glycine buffer system. The membranes were blocked for 1 h in skim milk [3% for anti-GFP (mouse monoclonal IgG, Roche, #11814460001); 5% for anti-TBP (rabbit polyclonal IgG, Santa Cruz, #sc-273)] with 0.1% Tween-20 in TBS prior to incubation with the primary antibodies. The membranes were blocked overnight at 4 °C in 3% skim milk for anti-Flag (mouse monoclonal IgG, M5, Merck, #F4042).

To detect the proteins of interest, the anti-GFP (1:1000 in 3% skim milk with 0.1% Tween-20 in TBS) and anti-TBP (1:500, in 5% skim milk with 0.1% Tween-20 in TBS) antibodies were incubated overnight at 4 °C, while anti-Flag (M5, 1:1500, in 3% skim milk with 0.1% Tween-20 in TBS) was incubated 1 h at RT. After washing five times for 5 min, the secondary antibody against mouse (1:5000, GE healthcare, NA931V) or against rabbit (1:5000, GE healthcare, NA934V) was applied. The membranes were finally incubated with ECL solution, and chemiluminescence was detected with either X-ray films (GE Healthcare, #70322) or chemiluminescence imaging system Vilber FUSION FX7 EDGE. The antibodies used in this study are given in Table S1.

### 2.13. Luciferase Assay

HeLa cells were seeded in 48-well plates at a density of 2.25 × 10^4^ cells/well. Transfection was performed with the Lipofectamine 2000 transfection reagent (Invitrogen, #11668019) using 0.25 μg/well of the reporter plasmid alone or together with various amounts of the expression plasmid (given in the corresponding figure legends). After 24 h, luciferase activity was determined from at least four independent experiments with 10 μL of cleared lysate in an LB 9501 luminometer (Berthold) by using the luciferase assay system from Promega (#E2980).

### 2.14. Fluorescence Microscopy

HeLa cells were plated (1 × 10^5^ cells/cm^2^) on chamber coverslips (Nunc-Lab-Tek, #155380). After 18 h, cells were transfected with 150 ng of GFP-RBPJL specific expression plasmids. 24 h after transfection, living cells were imaged using a fluorescence microscope (IX71, Olympus) equipped with a digital camera (C4742, Hamamatsu) and a 100-W mercury lamp (HBO 103W/2, Osram). Filter set for GFP detection: excitation, HQ470/40; emission, HQ525/50. Filter set for DAPI detection: D360/50; emission: D460/50. 

### 2.15. In Vitro Protein Translation

The in vitro protein translations were performed using the TNT-T7 coupled Reticulocyte lysate system (Promega, #L4610) according to the manufacturer’s instructions. Prior to electro mobility shift assays (EMSAs), the in vitro translations of wildtype (wt) and mutant RBPJL proteins were monitored by Western blotting using an anti-Flag antibody (M5, Merck, # F4042). 

### 2.16. Electro Mobility Shift Assay (EMSA)

Reticulocyte lysates (1 µL and 2 µL) from in vitro translations were used for electromobility shift assays (EMSAs). The binding reaction was performed in a buffer consisting of 10 mM Tris-HCl (pH 7.5), 100 mM NaCl, 0.1 mM EDTA, 0.5 mM DTT and 4% glycerol. For the binding reaction, 10 ng (0.02 U) poly(dI-dC) (GE Healthcare) and approximately 0.5 ng ^32^P-labeled oligonucleotides were added. The sequence of the double-stranded oligonucleotide FO233 (see Table S1) corresponded to the two RBPJ-binding sites within the EBV TP-1 promoter. DNA-protein complexes were separated using 5% polyacrylamide gels with 1× Tris-glycine-EDTA at RT. Gels were dried and exposed to X-ray films. 

### 2.17. Single Molecule Imaging and Residence Time Analysis

*Generation of stable cell lines and FACS sorting:* Halo-RBPJ-, Halo-RBPJ(R218H)- and Halo-RBPJL-expressing HeLa cell lines were generated by lentiviral transfection following a standard protocol (Addgene). For lentivirus production, Lenti-X-293T packaging cells were transiently transfected with psPAX2 (Addgene, #12260), pMD2.G (Addgene, #12259), and the transfer plasmid containing the tagged construct using JetPrime (Polyplus-transfection, #114-15). After 48 h of transfection, the virus was harvested by filtering the medium through a 0.45 µm membrane filter. HeLa cells were infected with filtered viral medium and incubated for 72 h at 37 °C and 5% CO_2_. Successfully transfected HeLa cells were sorted via FACS. For this, the cells stably expressing Halo-tagged RBPJ, RBPJ(R218H) and RBPJL were incubated with 1.25 µM Halo-Tag TMR ligand (Promega, #G8251) according to the manufacturer’s protocol. Unlabeled HeLa cells were used as negative control.

*Preparation of cells for imaging:* Cells were seeded on heatable glass bottom DeltaT dishes (Bioptechs) the day before imaging. On the next day, 3 pM silicon rhodamine (SiR) Halo-Tag ligand (kindly provided by Kai Johnson, MPI, Heidelberg, Germany) was applied to the cells for 15 min following the Halo-Tag staining protocol (Promega). On average, the labeling density was 6 spots per nucleus and frame. Subsequently, the cells were washed with PBS and recovered for 30 min in DMEM at 37 °C and 5% CO_2_. Afterwards, the cells were washed three times with PBS and imaged in 2 mL OptiMEM. 

*Microscope setup:* A custom-built fluorescence microscope (as described previously [31]) was used for single-molecule imaging. It contained a conventional Nikon body (TiE, Nikon) and was equipped with a 638 nm laser (IBEAM-SMART-640-S, 150 mW, Toptica), AOTF (AOTFnC-400.650-TN, AA Optoelectronics) and a high-NA objective (100×, NA 1.45, Nikon). The cells were illuminated with a highly inclined and laminated optical sheet (HILO) as described in [32]. The emitted fluorescence signal passed a multiband emission filter (F72-866, AHF, Tübingen, Germany) and was detected by an EMCCD camera (iXon Ultra DU 897, Andor, Belfast, UK). 

*Single molecule time-lapse imaging:* Time-lapse (tl) illumination with a fixed camera integration time of 50 ms and variable dark periods between two consecutive frames was performed in order to measure dissociation rates within a broad temporal range and to correct for photobleaching. Frame cycle times were 0.1 s, 0.4 s, 1.6 s, 6.4 s and 14 s for RBPJ, 0.1 s, 0.4 s, 1.6 s and 6.4 s for RBPJ(R218H) and 0.1 s, 0.4 s, 3.2 s and 14 s for RBPJL. Movies covered 30 s (0.1 s tl), 120 s (0.4 s tl), 480 s (1.6 s tl), 960 s (3.2 s tl and 6.4 s tl) and 1400 s (14 s tl). Before each measurement, the laser power was adjusted to 1.13 mW to avoid major differences due to photobleaching. 

*Single-molecule analysis using TrackIt:* Tracking analysis of single-molecule data was done with the software TrackIt [33]. Bright pixels were identified as fluorescent molecules if the signal-to-noise ratio (SNR) was above 4.5. To distinguish bound from diffusing molecules, we selected for tracks confined to a certain radius (tracking radius) for a certain time period (given by the minimum track length in units of frames). Tracking settings for tracking radius, minimum track length, gap frames and minimum segmentation length were adjusted for each time-lapse condition. The tracking radius was set to 0.9 pixels (0.1 s tl), 1.19 pixels (0.4 s tl), 1.75 pixels (1.6 s tl), 2.4 pixels (3.2 s tl), 2.8 pixels (6.4 s tl) and 3.1 pixels (14 s tl). The minimum track length was 3 frames for 0.1 s tl and 0.4 s tl and 2 frames for longer time-lapse conditions. To compensate the measurement noise, detected tracks were connected even if a molecule was not detected for a certain number of gap frames. In this study, 2 gap frames were allowed for 0.1 s tl, while 1 gap frame was allowed for the other tl conditions. The minimum segmentation length was set as 2 for all tl conditions.

*Survival-time distribution analysis using GRID:* For each tl condition, track lengths (in units of time) were combined and assembled to fluorescence survival-time distributions. The survival-time distributions of each construct were analyzed globally with GRID. GRID uses an inverse Laplace transformation to extract a dissociation rate spectrum from survival-time distributions [31,34]. This spectrum of dissociation rates (“event spectrum”) displays how frequently dissociation from a certain binding state with a corresponding dissociation rate occurs during an observation period. The event spectrum can be converted into a “state spectrum”, which gives information about the probability of a molecule to be in a certain binding state with a corresponding dissociation rate at any time snapshot. We report the state spectrum. To estimate the error of dissociation rates obtained by GRID, the analysis was repeated 499 times with 80% of the data for each run. The resampled GRID runs were merged into one state spectrum. The average dissociation rate and standard deviation of the slowest dissociation rate cluster was determined by manually setting borders that cover the resampled data. 

### 2.18. Statistical Analysis

Statistical tests and graphical data presentations were performed by means of GraphPad Prism 5.0 software. The statistical significance of differences between the indicated groups was tested by using the unpaired Student’s *t*-test. All data represent the mean ± s.d. (standard deviation) of three independent experiments. The level of statistical significance is presented by asterisks (*) *p*-value (*p*) > 0.05 = (NS), *p* ≤ 0.05 = (*), *p* ≤ 0.01 = (**), *p* ≤ 0.001 = (***). 

### 2.19. Antibodies, Plasmids, Oligonucleotides and Reagents

Detailed information is given in supplementary Table S1. 

## 3. Results

### 3.1. Structural Conservation of Transcription Factors RBPJ and Its Paralog RBPJL

**Figure 1:**
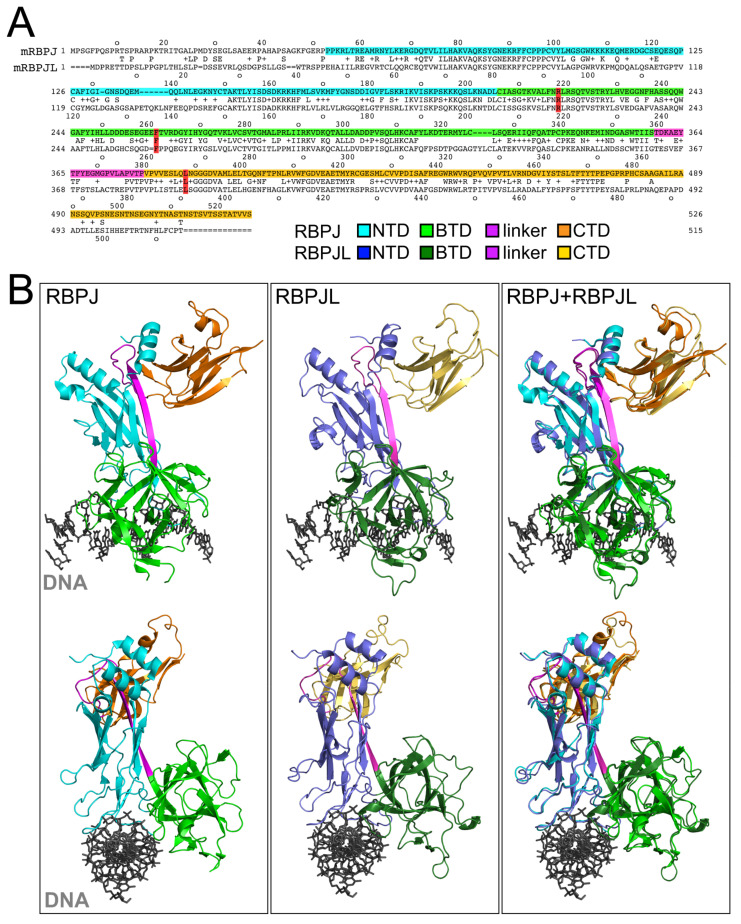
Comparison of RBPJ and RBPJL: (**A**) Protein sequence alignment of mouse RBPJ and mouse RBPJL. RBPJ consists of three domains: the NTD (N-terminal domain, cyan), the BTD (beta-trefoil domain, green), and the CTD (C-terminal domain, orange). The “linker region” between the BTD and the CTD is highlighted in magenta. The numbers indicate the amino acid positions. Residues within RBPJ critical for DNA binding (R218) and SHARP binding (F261 and L388, highlighted in red) are conserved between RBPJ and RBPJL. (**B**) Structural alignment of RBPJ and RBPJL in complex with DNA based on homology modeling. Structure of RBPJ bound to DNA (left; PDB entry 3BRG), RBPJL bound to DNA (middle) and the structural alignment of both complexes (right) reveal a high conservation on the structural level. The NTD, BTD and CTD of RBPJ are presented in the same color code as in (**A**). The putative homolog domains within RBPJL are labeled in dark blue (NTD), dark green (BTD) and dark yellow (CTD). The linker region is also colored in magenta. The DNA is colored in gray. Lower panels show the complexes after 90° rotation around a vertical axis revealing the responsible DNA binding regions of RBPJ and RBPJL. All structures, as well as the alignment, were performed using PyMOL (https://www.pymol.org, accessed on 2 April 2020). Color schemes are formated as in (**A**).

When comparing the primary amino acid sequence of RBPJ and its paralog RBPJL, the overall sequence similarity is low (Needleman-Wunsch alignment tool [35]: 49% identities, 64% positives, 6% gaps) (Figure 1A). However, the three structural domains NTD (N-terminal domain, cyan), BTD (beta-trefoil-domain, green) and CTD (C-terminal domain, orange) are apparent. A detailed analysis for human and mouse RBPJ and RBPJL alignments is given in Supplementary Table S1.

In order to compare the overall structure of RBPJ with RBPJL, we took advantage of the available X-ray structure of RBPJ in complex with DNA (Figure 1B, left side). Furthermore, for RBPJL, we used a crystal structure modeling tool (homology modeling by swissmodel) (Figure 1B, middle). The structure alignment revealed a high degree of similarity. The same was true when we aligned the human RBPJ crystal structure (pdb entry 5EG6) with human RBPJL structure models from swissmodel, alphafold2.0 [26] and robetta [27] (Figure S1). The latter two use a deep learning modeling approach instead of homology modeling. Again, the functional domains of RBPJ (NTD, BTD and CTD) are clearly aligning within the two structures (Figure 1B, right side). The linker regions of RBPJL are markedly different though the length is conserved. Importantly, the amino acid residues previously implicated in DNA binding (R218) and cofactor binding (F261 and L388) of RBPJ are conserved (RBPJL: R220, F262, L393). These amino acids are highlighted in red in the primary amino acid sequences (see Figure 1A). 

### 3.2. Expression of RBPJL Is Highly Specific and Overlaps with PTF1a

**Figure 2:**
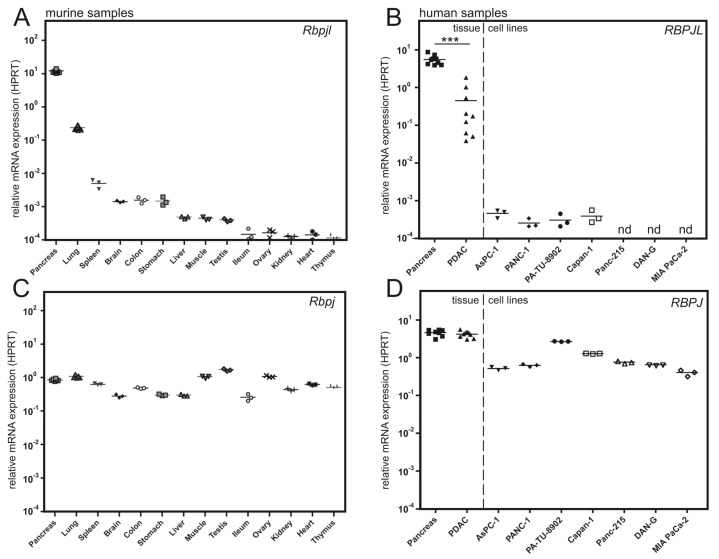
mRNA expression of RBPJL (**A**,**B**) and RBPJ (**C**,**D**) in murine and human tissue samples and PDAC cell lines. (**A**) Relative mRNA expression of Rbpjl in tissues from C57BL/6J mice analyzed by qRT-PCR. Rbpjl shows specific expression in pancreas (high), lung (median), spleen (low), brain, colon and stomach (very low). In the other tissues, Rbpjl mRNA is barely detectable. (**B**) Relative mRNA expression of RBPJL in human pancreas, PDAC and PDAC cell lines. Expression of RBPJL is downregulated in PDAC and lost in PDAC cell lines. (**C**,**D**) mRNA expression of Rbpj shows no significant tissue specificity in mice (**C**) and only a modest down regulation in some human PDAC cell lines compared to pancreatic tissue. All mRNA expressions levels were normalized by the HPRT housekeeping gene. *** *p* < 0.001, unpaired Student’s *t*-test.

We compared relative mRNA levels of RBPJL (Figure 2A,B) and RBPJ (Figure 2C,D) in different tissues from *Mus musculus* and *Homo sapiens* by qRT-PCR. 

The expression of RBPJ is ubiquitous, also clearly detectable in human pancreatic tissue, PDAC and pancreatic cancer cell lines (Figure 2D). In contrast, RBPJL expression is highly expressed in the pancreas in both mouse (Figure 2A) and human (Figure 2B). Surprisingly, in human PDAC samples RBPJL is significantly less expressed compared to RBPJ (compare Figure 2B,D). In addition, RBPJL expression is almost undetectable in human PDAC cell lines. Since tumor cells resemble a ductal fate in PDAC, we hypothesized that RBPJL not only is a pancreas specific marker, but more specifically, is an acinar marker of the pancreas. Therefore, we re-analyzed single-cell RNAseq data from human adult pancreas samples (GSE81547, [29]) with regard to the expression of the two paralogs RBPJ and RBPJL. Again, RBPJ is expressed in all subtypes of cells, including acinar-, ductal- and mesenchymal types (compare Figure S2A with Figure S2B). PTF1a is a well-known acinar marker, and, when mapping RNA-levels in single cells, the overlap is clearly in the acinar fraction (upper left) and a small amount in the progenitor fraction, see Figure S2C. The expression of RBPJL is almost identical to PTF1a expression (compare Figure S2C with Figure S2D). In addition, when we used a well-established acinar-to-ductal differentiation model ex vivo by adding TGFα to freshly isolated and dissociated pancreata from wildtype mice, ductal differentiation is evident after three days (Figure S3A, inlay at lower right). This acinar to ductal differentiation can be monitored by qRT-PCR showing the upregulation of the ductal marker cytokeratine 19 (Ck19) together with a downregulation of the acinar marker Ptf1a, amylase (Amy2a2) and again Rbpjl (Figure S3B). Together, RBPJL expression is specifically restricted to the pancreatic acinar lineage and strongly induced therein, whereas RBPJ is more ubiquitously expressed. 

### 3.3. RBPJL Does Not Interact with the Coactivator NICD

**Figure 3:**
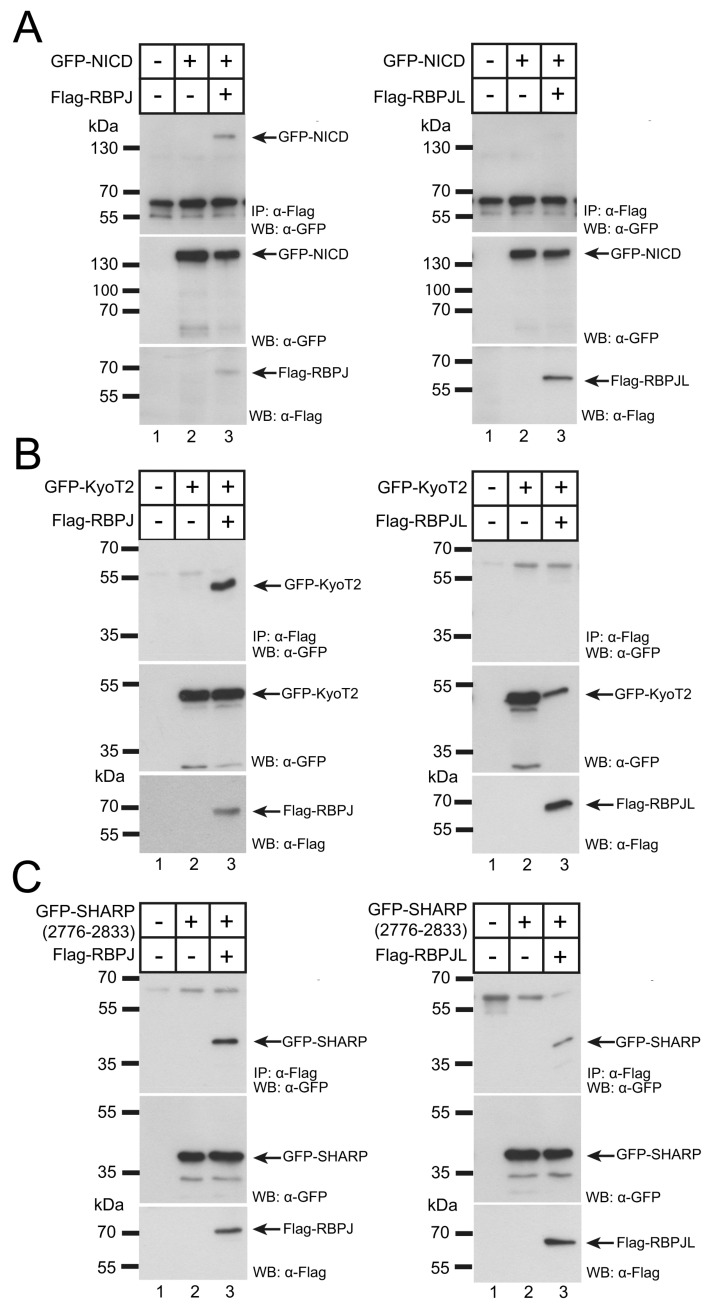
RBPJL does not interact with RBPJ “RAM”-type binding proteins (NICD, KyoT2) but does interact with corepressor SHARP. In contrast to RBPJ (left panels), coimmunoprecipitations (CoIPs) show no binding of RBPJL to NICD (**A**, right) and KyoT2 (**B**, right). (**C**) RBPJL interacts with corepressor SHARP (right) and with RBPJ (left). HEK293 cells were cotransfected with Flag-tagged RBPJ or RBPJL together with the indicated GFP-tagged constructs: NICD (which corresponds to the human NOTCH1 intracellular domain, aa 1761-2555), KyoT2 and SHARP (aa 2776-2833 correspond to the RBPJ interaction domain, RBPID). CoIPs were performed 24 h after transfection. Immunoprecipitation was performed using an anti-Flag antibody and coimmunoprecipitated proteins were detected by using an anti-GFP antibody (upper panels). Expression of proteins was verified by Western blotting (middle panels and lower panels). Original blots see Figure S8.

Transcription factor RBPJ is known to interact not only with DNA but also with the NICD and mechanistic details have been studied in great detail not only structurally but also biochemically and functionally [36,37] and reviewed in [38]. The NICD contacts the BTD and CTD domains of RBPJ, and this binding surface is conserved not only for NICD but also for additional cofactors KyoT2/FHL1 [39] and RITA [28]. Whereas RBPJ strongly interacts in co-immunoprecipitation experiments with NICD (Figure 3A, left), KyoT2/FHL1 (Figure 3B, left) and RITA (Figure S4A, left), RBPJL does not interact with NICD (Figure 3A, right), KyoT2 (Figure 3A, right) or RITA (Figure S4A, right). As a positive control, we used PTF1a, which was previously described as strongly interacting with RBPJL. This was also the case in our co-immunoprecipitation experiments: Both RBPJ and RBPJL were able to interact with PTF1a (Figure S4B). Importantly, both RBPJ and RBPJL also showed an interaction with the corepressor SHARP (Figure 3C). In summary, differently from RBPJ, RBPJL does not interact with the classical RAM-like binding partners NICD, KyoT2 or RITA but does interact with the Notch corepressor SHARP. 

**Figure 4:**
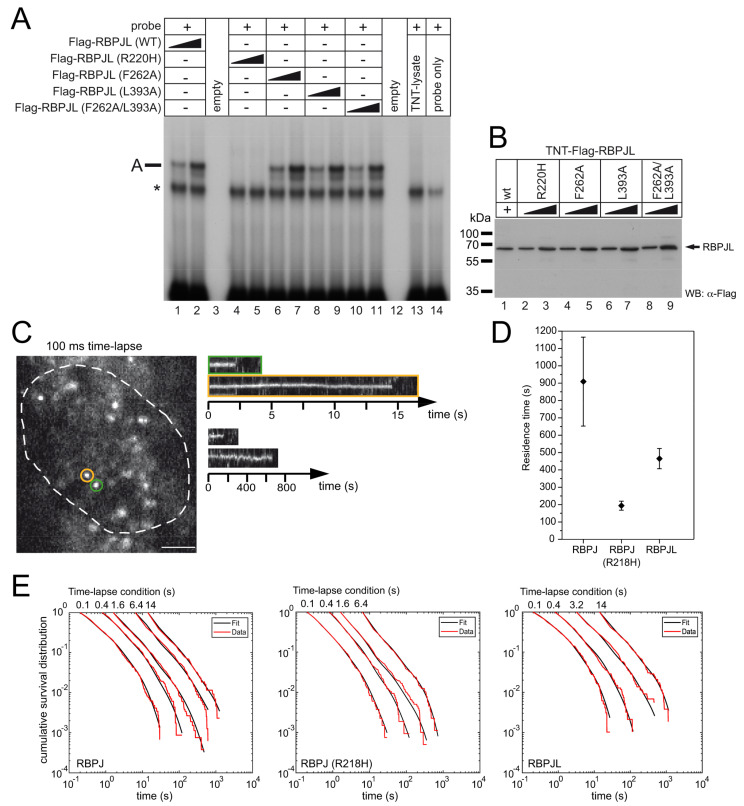
Nuclear binding of RBPJL compared to RBPJ. (**A**) EMSA analysis of in vitro translated wildtype RBPJL and mutated RBPJL proteins used in the study. RBPJL (wt) and mutants (F262A, L393A and F262A/L393A) show unchanged DNA-binding capacity to the canonical RBPJ binding sequence. Only the BTD-mutant R220H has lost DNA-binding capacity (lanes 4,5) The RBPJL-DNA binding complexes are labeled A (lane 1, 2, 6–11). The asterisk highlights an unspecific binding complex also seen in the negative controls (lanes 13 and 14). The ^32^P-labeled oligonucleotide (s) FO233F/R was used as probe. (**B**) Quality of RBPJL proteins after in vitro translation was verified by Western blotting using an anti-Flag antibody. Increasing amounts of TNT lysates (1 µL and 2 µL) were used for EMSA and Western blot. Original blots see Figure S8. (**C**–**E**): Comparison of residence times of RBPJ, RBPJ (R218H) and RBPJL in the nucleus of living cells. (**C**) Single-molecule fluorescence image of SiR-labeled HaloTag-RBPJ with 50 ms acquisition time. Scale bar denotes 3 µm. Right panels: Kymographs of the green and orange circled molecules of the 100 ms time-lapse movie and of molecules from a 14 s time-lapse measurement. (**D**) Residence times of RBPJ, RBPJ(R218H) and RBPJL calculated using the slowest dissociation rate cluster of the state spectra obtained by GRID. Error bars the denote standard deviation of the spectrum resampled 499 times with 80% of the data. (**E**) Cumulative survival time distribution of SiR-HaloTag-RBPJ, SiR-HaloTag-RBPJ(R218H) and SiR-HaloTag-RBPJL (red lines) at the time-lapse conditions indicated on top and survival-time functions obtained by GRID (black lines). Number of bound molecules/total number of molecules: RBPJ: 1459/19835 (100 ms time-lapse); 1149/19921 (400 ms time-lapse); 2648/26782 (1.6 s time-lapse); 1584/19203 (6.4 s time-lapse); 434/5593 (14 s time-lapse). RBPJ(R218H): 1329/16990 (100 ms time-lapse); 1064/20562 (400 ms time-lapse); 1978/22143 (1.6 s time-lapse); 882/11619 (6.4 s time-lapse). RBPJL: 975/19647 (100 ms time-lapse); 940/19921 (400 ms time-lapse); 878/12865 (3.2 s time-lapse); 525/7662 (14 s time-lapse).

To further characterize the molecular mechanism of RBPJL action, we took advantage of the combined structural and functional data of its paralog RBPJ [19] and the sequence comparison of RBPJL with RBPJ (Figure 1 and Figure S1). Subsequently, we generated RBPJL mutants at the positions R220H, F262A and L393A and the double mutant F262A/L393A (corresponding to the residues R218, F261 and L388 in RBPJ). These residues where shown to be involved in DNA binding and/or cofactor interaction of RBPJ [19,25]. We tested the ability of the corresponding mutants to bind DNA in electrophoretic-mobility-shift assays (EMSA) using a double-stranded oligo containing two TGGGAA-motifs representing a canonical RBPJ DNA-binding site (Figure 4A).

In vitro translated RBPJL variants used for the DNA binding assays were tested by Western blotting (Figure 4B). As expected, the R220H-mutant RBPJL was defective in DNA binding (Figure 4A, lane 4, 5), whereas all the other mutants were able to bind to DNA. 

Moreover, we compared the binding behaviour of RBPJ and RBPJL in the nucleus of live cells using single-molecule tracking (Figure 4C and Methods) [31,33]. To visualize single molecules, we created HeLa cell lines stably expressing RBPJ or RBPJL fused to a HaloTag [40], which we labeled with the organic dye SiR before imaging [41]. We enabled long observation times using time-lapse microscopy with 50 ms frame acquisition time and frame cycle times between 0.1 s and 14 s (see methods for details). Tracks of individual molecules, analyzed with TrackIt [33], revealed binding events in the nucleus of up to several hundred seconds (Figure 4C). We collected the binding times of each time-lapse condition and analyzed the resulting fluorescence survival-time distributions (Figure 4D) with the method GRID, which reveals spectra of dissociation rates [34]. Binding times can be calculated from these dissociation rate spectra by taking the inverse value. The dissociation rate spectra we obtained for both RBPJ and RBPJL were complex with several dissociation rate clusters (Supplementary Figure S6). For RBPJL, the longest binding time, corresponding to the dissociation rate cluster with smallest value, was reduced compared to RBPJ (Figure 4E).

To obtain further insight into the molecular underpinnings of the dissociation rate spectrum of RBPJ, we performed analogous measurements on the mutant RBPJ (R218H) [42], whose ability to bind DNA was disturbed—(Figure 4D and Supplementary Figure S6). For this mutant, binding events in the time-lapse condition of the longest frame cycle time of 14 s were extremely rare, wherefore we excluded this condition from the analysis. Compared to RBPJ, the longest binding time of RBPJ (R218H) was considerably reduced (Figure 4E). This indicates that the longest binding time of RBPJ is associated to DNA binding. 

In our comparison of the live-cell binding of RBPJ and RBPJL, we thus focused on the longest binding time (Figure 4E). We found the longest binding time was 910s (±256 s, mean ± s.d. from resampling) for RBPJ, compared to 194 s (±26 s, mean ± s.d. from resampling) for RBPJ(R218H) and 465 s (±58 s, mean ± s.d. from resampling) for RBPJL. Binding times in the range of minutes have also been reported for SRF [43], CDX2 [34], TBP [44], LacI [45] and TetR [46]. The two-fold difference in binding time between RBPJ and RBPJL might reflect the differences in complex composition of the two factors (see Figure 4 and Figure S6).

### 3.4. RBPJL Does Not Support Notch-Mediated Transactivation

Next, we performed functional Notch-dependent luciferase assays in RBPJ-depleted HeLa cells, reconstituted with either RBPJ or RBPJL. RBPJ was previously shown to support transcriptional activation together with NICD using a reporter gene construct containing 12 perfect RBPJ binding sites [47].

**Figure 5:**
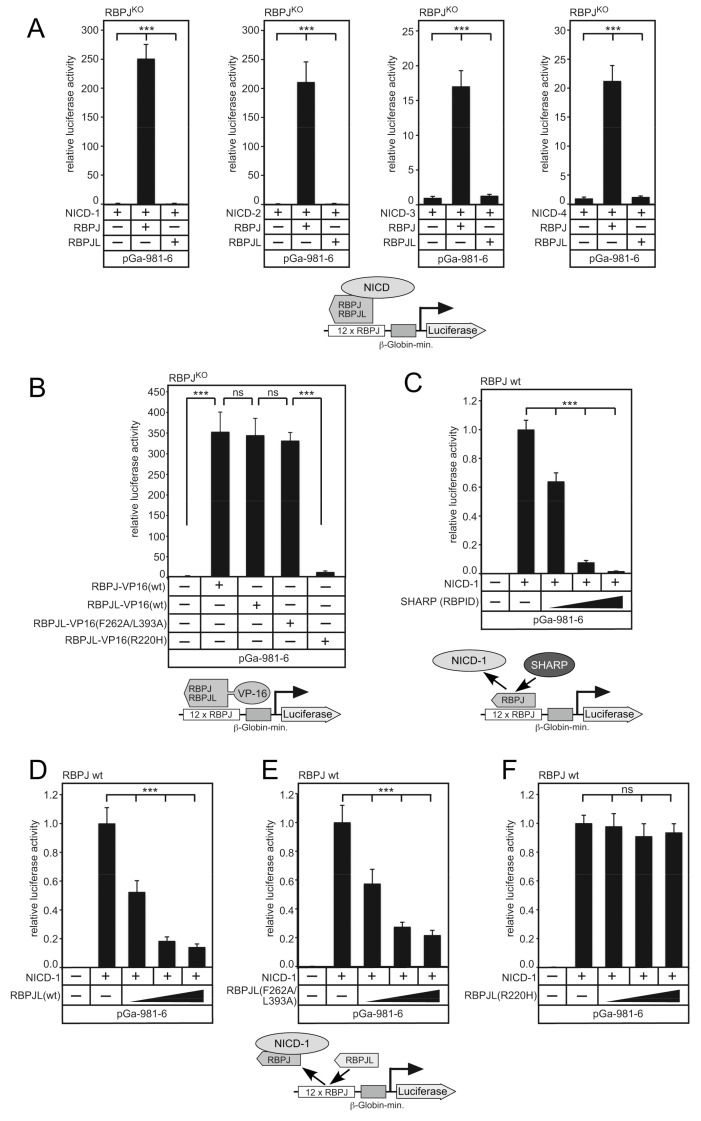
RBPJL binds to the canonical RBPJ-DNA binding sequence but cannot transactivate together with NICD1–4 proteins. (**A**) In contrast to RBPJ, RBPJL is not able to transactivate a Notch-dependent reporter together with the mammalian NICD proteins. HeLa^RBPJ-KO^ cells were transfected with the luciferase reporter construct pGa981/6 (250 ng) and with plasmids expressing NICD-1, -2, -3, -4 (10 ng), alone or together with either RBPJ (100 ng) or RBPJL (100 ng). Lower panel illustrates the reporter construct and protein expression in the transcription assay. (**B**) RBPJL fused to VP-16 is able to transactivate a Notch/RBPJ-dependent reporter. The pGa981/6 luciferase reporter construct (250 ng) was transfected alone or together with plasmids expressing either RBPJ-VP16(wt) (50 ng), RBPJL-VP16 (wt) (50 ng), RBPJL-VP16 (F262A/L393A) or RBPJL-VP16 (R220H) into Hela^RBPJ-KO^ cells. Lower panel illustrates the reporter construct and protein expression in the transcription assay. (**C**) Corepressor SHARP represses Notch dependent transactivation through the displacement of NICD from the Notch coactivator complex. The luciferase reporter construct pGa981/6 (250 ng) was transfected alone or together with either NICD (10 ng) alone or together with increasing amounts (50 ng, 100 ng and 150 ng) of SHARP expressing plasmids into HeLa cells. Lower panel illustrates the reporter construct and the proposed displacement mechanism. (**D**) RBPJL(wt) is able to displace the RBPJ/NICD coactivator complex at canonical RBPJ binding sites. (**E**,**F**) While the RBPJL mutant (F262A/L393A) is also able to displace the RBPJ/NICD coactivator complex complex similar to wildtype RBPJL (**E**), the RBPJL DNA binding mutant (R220H) is unable to do so (**F**). The luciferase reporter construct (250 ng) was transfected alone or together with either NICD (10 ng) or together with increasing amounts (50 ng, 100 ng and 150 ng) of RBPJL-expressing plasmids into HeLa cells. Lower panel illustrates the reporter construct and the proposed displacement mechanism. Luciferase activity was determined from total-cell extracts and normalized to the basal promoter activity of the reporter construct. Mean values and standard deviation are from six independent experiments, ns: not significant, *** indicates statistical significance (*p* < 0.001, Student’s *t*-test) compared to control.

Indeed, as shown in Figure 5C, NICD-mediated transactivation was strongly reduced after expression of SHARP. Since RBPJL and RBPJ bound to the same DNA sequence, we wanted to know if RBPJL was able to replace the whole RBPJ-NICD coactivator complex. Activated luciferase activity was significantly reduced after the coexpression of RBPJL (wt) and the RBPJL mutant (F262A/L393A) in a dose-dependent manner (Figure 5D,E). However, the DNA binding mutant RBPJL (R220H) was unable to reduce RBPJ-NICD transactivation. Thus, RBPJL is able to disturb Notch mediated transcription through the replacement of the RBPJ-NICD coactivator complex. 

### 3.5. RBPJL-SHARP Interaction Depends on Conserved Amino Acid Residues

Since we have shown that corepressor SHARP interacts with RBPJL (Figure 3C) using the same domain within SHARP (RBP Interaction Domain; RBPID) as for RBPJ binding, we wanted to investigate the interaction between RBPJL and SHARP in more detail. 

**Figure 6:**
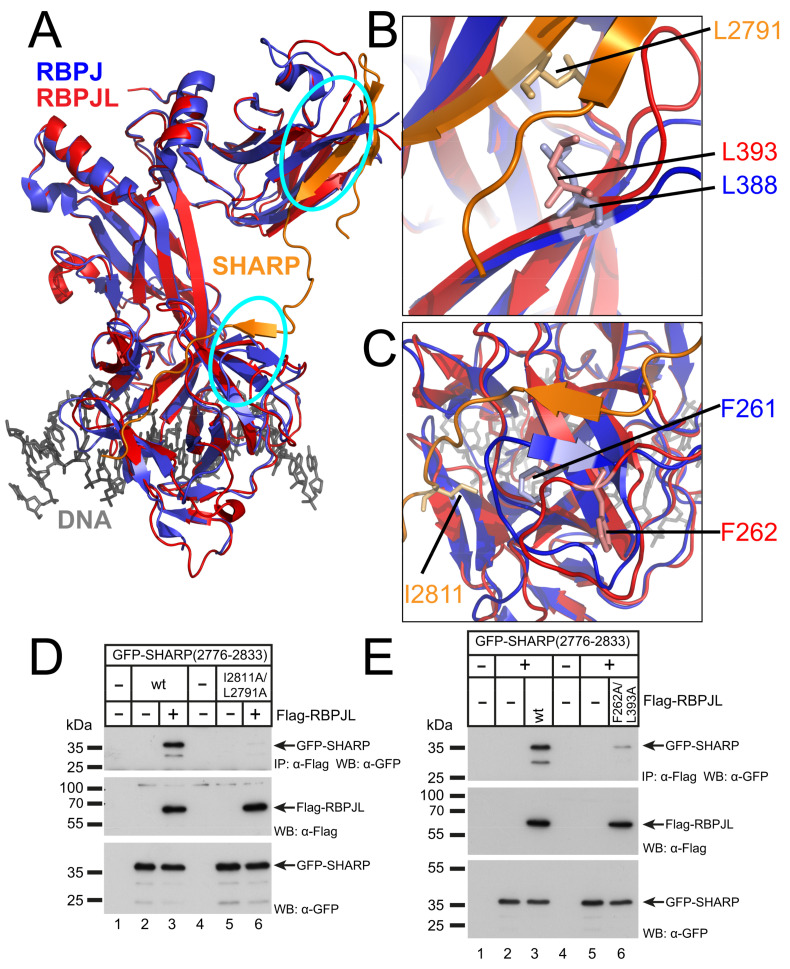
Conserved amino acid residues involved in RBPJL-SHARP interaction. (**A**) Structural alignment of the RBPJ-SHARP complex (PDB: 6DKS) with RBPJL (**B**) Zoom into the C-terminal domain (CTD) shows the localization of critical residues L2791 of SHARP (orange), L388 of RBPJ (blue) aligned with L393 of RBPJL (red). (**C**) Zoom into the beta-trefoil domain (BTD) shows the localization of critical residues I2811 of SHARP (orange), F261 of RBPJ (blue) aligned with F262 of RBPJL (red). (**D**) SHARP mutant I2811A/L2791A does not interact with RBPJL (compare lane 3 to 6). HEK293 cells were cotransfected with GFP-tagged SHARP (aa 2776-2833) wildtype or the I2811A/L2791A mutant and Flag-tagged RBPJL. CoIPs were performed 24 h after transfection using an anti-Flag antibody. (**E**) Mutant RBPJL (F262A/L393A) has lost most of the SHARP binding capacity. GFP-SHARP (aa 2776-2833) and Flag-tagged RBPJL wt or F262A/L393A mutant constructs were transfected into HEK293 cells. CoIPs were performed 24 h after transfection using an anti-Flag antibody. Expression of Flag-RBPJL (middle panel) and GFP-SHARP proteins (bottom panel) was verified by Western blotting. Original blots see Figure S8.

Therefore, we aligned the structure of the RBPJ-SHARP complex [19] (PDB: 6DKS) with the RBPJL structure model using PyMol software (Figure 6A). Previously, the co-crystal structure of RBPJ and the SHARP RBPID revealed that there are two interaction surfaces for SHARP on RBPJ (Figure 6A, cyan circles) and that amino acid residues L388 and F261 within RBPJ are necessary for SHARP binding [19]. In addition, two residues (L2791, I2811) were identified within the SHARP RBPID, critical for RBPJ binding. 

When comparing the RBPJ-SHARP complex with RBPJL in higher resolution, the structural overlap was recognized (Figure 6B,C). Thus, we used the RBPJ binding defective (L2791A/I2811A) SHARP RBPID (Figure 6D) in coimmunoprecipitation experiments with RBPJL (wt). The SHARP-mutant that no longer interacted with RBPJ was also deficient for RBPJL binding (Figure 6D) comparing wildtype-SHARP in lane 3 with mutant SHARP in lane 6. Next, we analyzed RBPJL mutants F262A, L393A and the double mutant F262A/L393A.

The corresponding amino acids within RBPJ are involved in SHARP interaction and show a high degree of three-dimensional alignment in the predicted structure of RBPJL (Figure 6A,B). Coimmunoprecipitation assays with the SHARP RBPID (2776-2833) revealed that the double mutant RBPJL (F262A/L393A) interacts significantly weaker than wildtype-RBPJL (Figure 6E). Taken together, the amino acid residues critical for SHARP-RBPJ interaction are also involved in SHARP-RBPJL interaction. Therefore, the binding mechanism of corepressor SHARP seems to be conserved within RBPJL. 

### 3.6. RBPJL Can Reconstitute Transcriptional Repression of Endogenous Notch Target Genes

**Figure 7:**
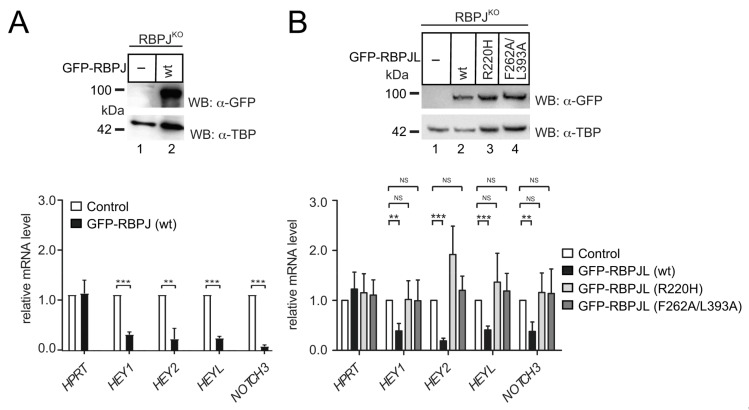
Reconstitution of Notch target gene repression in RBPJ knockout cells by RBPJ and RBPJL: (**A**) RBPJ is able to reconstitute repression of Notch target genes in RBPJ depleted cells. Cells were stably transfected with GFP-tagged RBPJ fusion protein. RBPJ expression and TBP expression were analyzed by Western blotting (upper). Total RNA was purified and analyzed by qRT-PCR with primers specific for Notch target genes HEY1, HEY2, HEYL and NOTCH3 in GFP-RBPJ transfected cells (lower). (**B**) RBPJL wt but neither the DNA binding mutant (R220H) nor the SHARP binding mutant (F262A/L393A) are able to reconstitute transcriptional repression of Notch-target genes in RBPJ knockout cells. Cells were transfected with RBPJL (WT, R220H, F262A/L393A) constructs. RBPJL fusion proteins and TBP were analyzed by Western blotting (upper). Notch-target genes were analyzed by qRT-PCR in transfected cells (RBPJL WT, R220H, F262A/L393A) (lower). All mRNA expression levels were normalized to the expression of the HPRT housekeeping gene. Mean values and standard deviation are from four independent experiments (** *p* < 0.01, *** *p* < 0.001, ns = not significant, unpaired Student’s *t*-test). Original blots see Figure S8.

Having shown the RBPJL-SHARP interaction, we wanted to characterize the functional consequences and determine the repressive capacity of RBPJL at endogenous Notch target genes. As we previously found that the deletion of RBPJ results in the derepression of Notch target genes in a mature T cell line [19] and in HeLa cells [48], we now performed rescue experiments re-expressing RBPJ or RBPJL fused to GFP using RBPJ-depleted HeLa cells (Figure 7A,B). 

Consistent with our former findings, the repression of Notch target genes Hey1, Hey2, HeyL and Notch3 was reconstituted. Expression was significantly reduced relative to house-keeping gene HPRT after re-expressing RBPJ in RBPJ-depleted HeLa cells (Figure 7A, lower panel). Next, we used RBPJ-depleted HeLa cells to stably express wildtype RBPJL, the DNA-binding defective RBPJL (R220H) and the SHARP-binding defective RBPJL (F262A/L393A) fused to GFP (Figure 7B). The protein expression of RBPJL and mutant-RBPJL was comparable (Figure 7B upper panel), and the cellular localization of RBPJL DNA-binding defective mutant (R220H) and SHARP-binding defective mutant (F262A/L393A) was comparable to that of wildtype RBPJL (Figure S5). Again, we measured the gene expression levels of several Notch target genes by qRT-PCR. Importantly, only wildtype RBPJL but not DNA binding defective (R220H) nor SHARP binding defective RBPJL (F262A/L393A) could rescue the transcriptional repression of endogenous Notch targets (Figure 7B, lower). 

Taken together, we conclude that RBPJL, the distantly related paralog of RBPJ, can indeed functionally compensate the repression capability of RBPJ and acts as a transcriptional repressor, most likely by recruiting the corepressor SHARP. 

### 3.7. Expression of RBPJL in a Tumorigenic Context

To gain insight on the role of RBPJL in cancer, we looked for the expression of RBPJL in several cell lines. Since specificity towards RBPJL of commercial anti-RBPJL antibodies was low, we consulted publicly available databases, for example the human protein atlas [49]. We validated the observed specific expression pattern in several AML cell lines using qRT-PCR. Surprisingly, in selected myeloid leukemia cell lines U937 (histiocytic lymphoma) and NB-4 (acute promyelocytic leukemia) we found RBPJL expression levels comparable to that of RBPJ. In THP-1 cells (acute monocytic leukemia), RBPJL expression was detectable, but less than that of RBPJ. (Figure S7A–C). In an unrelated colon cancer cell line, HCT-116, RBPJL was barely detectable (Figure S7D). Thus, it is possible that RBPJL offers a selective advantage for certain subtypes of myeloid leukemia, even in the absence of PTF1a, most probably deregulating Notch target genes. 

## 4. Discussion

Here, we have shown that RBPJL is a highly specific acinar marker and is significantly downregulated in PDAC and several PDAC cell lines. Although the sequence conservation between RBPJ and RBPJL is low, RBPJL is capable of replacing RBPJ with regard to transcriptional repression. Interestingly, RBPJL is re-expressed in leukemia (AML). 

### 4.1. RBPJL as an Acinus-Specific Exocrine Marker

RBPJL expression was already previously described as tissue-specific to the pancreas [21] but also to a lesser extent in the brain, spleen and lung [50], whereas the expression of RBPJ is ubiquitous. The highly specific expression as an acinar marker is in line with RBPJL’s function within the PTF1a-complex. Data from the McDonald laboratory strongly support an important function for RBPJL in the expression of acinar gene expression, due to its role within the activating PTF1a-trimeric complex [20]. Our rescue-experiments in RBPJ-depleted cells indicate that RBPJL also plays a PTF1a-independent role at bona fide Notch target genes. This is one aspect of RBPJL function, but the complete lack of interaction with all the different Notch-coactivators (NICD1, -2, -3 and -4) might be another. Our data argues for an additional role of RBPJL at Notch target genes. The strong expression of RBPJL will support repression but not Notch-mediated transactivation. Concerning diagnostic value, RBPJL can clearly serve as a negative marker for PDAC (loss of RBPJL expression) and could be potentially used for transdifferentiation experiments as a highly specific acinar marker. 

### 4.2. Functional Comparison between RBPJL and RBPJ

RBPJ and RBPJL, despite their limited amino acid sequence homology, are predicted to be structurally similar. Using reporter-gene assays, EMSA-assays and single-molecule tracking, we show the two paralogs exhibit comparable but not identical residence times within the minute (s) range. However, differences in complex formation capabilities of these two factors might result in overall shorter residence times of RBPJL compared to RBPJ, as revealed by our single-molecule experiments. A similarity of both paralogs has also been observed for their role within the PTF1 complex [21,23]. Although the DNA-binding specificity of the two paralogs is comparable, the cofactor binding and tissue expression is clearly different. It is striking that RBPJL displays such a tissue-specific expression pattern, especially in the pancreas, while its paralog RBPJ is ubiquitously expressed. Apart from its undisputed role within the PTF1 complex, in our view, it might also have a role as a functional opponent of RBPJ. It is known that RBPJ can bind to cofactors harboring a WxP motif including Notch1-4, KyoT2/FHL1 [36,37,38] and RITA [17]. A WxP motif binding surface is not conserved in RBPJL as presented biochemically in the present study. However, the binding to the central corepressor SHARP is conserved between RBPJ and RBPJL, and mutating the SHARP binding surface within RBPJL leads to the loss of repression. In the future, ChIPseq experiments for the genome-wide binding of RBPJL are required to unequivocally address direct gene regulation of RBPJL. Unfortunately, we were unable to perform such experiments due to a lack of suitable anti-RBPJL antibodies. Our data also strongly suggest an important role for cofactor SHARP in pancreas development and also for terminal acinar differentiation (or transdifferentiation). SHARP (MINT) knockout mice are embryonic lethal [51] and have not been analyzed with regard to pancreas development in detail. Conditional targeting of SHARP (MINT) [52] might allow to address its potentially important role in the pancreas in future experiments.

### 4.3. Re-Expression of RBPJL in Cancer

Expression levels of RBPJL are increased in certain cell lines, such as myeloid leukemia cell lines NB-4, U-937 and THP-1. Interestingly, in the myeloid lineage Notch signaling inhibits the growth and survival of myeloblastic leukemia, reviewed in [53]. Thus, it is tempting to speculate that the expression of RBPJL, which only represses but does not coactivate together with Notch, might be a selection advantage in certain cancer types. 

Along these lines, a tumour-suppressive role for enhanced Notch signaling has been postulated in skin cancer [54]. Thus, it will be interesting to see whether RBPJL expression can be associated with certain types of cancer in the clinical setting.

## 5. Conclusions

**Figure 8:**
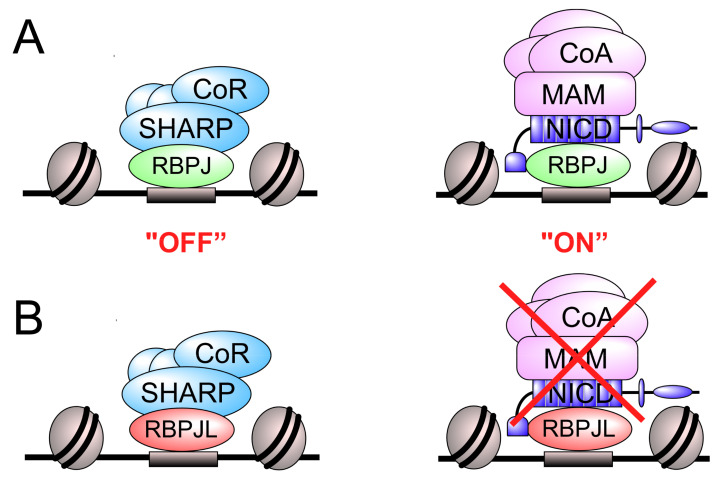
Model of RBPJ vs. RBPJL specific transcription complexes. (**A**) In the absence of activated Notch signaling, the RBPJ-SHARP complex represses the Notch target genes by recruiting corepressors (CoR; repressed state, left). Upon ligand binding to Notch receptor, the NICD is released, translocates to the nucleus and interacts with the transcription factor RBPJ. The RBPJ-NICD complex recruits Mastermind (MAM) and additional coactivators (CoA), and thereby activates Notch target gene expression (active state, right). (**B**) Proposed model of repression of Notch target genes via the RBPJL-SHARP complex in the absence of RBPJ. In RBPJ-depleted HeLa cells, the RBPJL interacts with SHARP and represses the Notch target genes by recruiting corepressors (left). However, RBPJL is unable to form a coactivator complex with NICD (right).

Here, we have shown that RBPJL, the pancreas-specific paralog of RBPJ, is a novel, highly specific exocrine marker. RBPJL is partially able to compensate for loss-of RBPJ concerning the gene repression of Notch target genes. RBPJL is able to recruit the corepressor SHARP/HDAC complex but is unable to facilitate Notch-mediated transactivation (Figure 8). Thus, in addition to its positive regulatory role in the PTF1-complex, RBPJL is able to repress Notch target gene expression. 

## Data Availability

Not applicable.

## Supplementary Materials

The following are available online at https://www.mdpi.com/article/10.3390/cancers13195027/s1, Figure S1: Structure prediction of RBPJL and alignment with the RBPJ crystal structure, Figure S2: *RBPJL* is a highly specific acinar marker, Figure S3: *Rbpjl* is downregulated during acinar to ductal differentiation ex vivo, Figure S4: RBPJL does not interact with RBPJ-“RAM”-type binding protein RITA but interacts with Ptf1a, Figure S5: Subcellular localization of GFP-RBPJL variants, Figure S6: State spectra of RBPJ, RBPJ (R218H) and RBPJL, Figure S7: Expression of RBPJL in non-pancreatic tumour cells, Figure S8: Original western blots. Table S1: qRT-PCR-Assays, Plasmids, Oligonucleotides, Reagents and Alignment Analysis.
