# Supplementary material for: Transcription Factor RBPJL Is Able to Repress Notch Target Gene Expression but Is Non-Responsive to Notch Activation
Source: Cancers (Basel). 2021 Oct 8;13(19):5027. doi: 10.3390/cancers13195027 (PMC8508133; doi:10.3390/cancers13195027)
Supplement: Supplementary file 1 [file cancers-13-05027-s001.zip › ijms-1395772-supplementary.pdf]

**SUPPLEMENTARY MATERIAL**

# Novel synthesized *N*-ethyl-piperazinyl-amides of C2-substituted oleanonic and ursonic acids exhibit cytotoxic effects through apoptotic cell death regulation

**Oxana Kazakova[1#*], Alexandra Mioc[2,3#], Irina Smirnova[1], Irina Baikova[1], Adrian Voicu[5*], Lavinia Vlaia[2,4], Ioana Macașoi[2,3], Marius Mioc[2,3], George Drăghici[2,3], Ştefana Avram[2,3], Cristina Dehelean[2,3], Codruța Şoica[2]**

1 Ufa Institute of Chemistry, UFRC RAS, 71, pr. Oktyabrya, Ufa, 450054, Russian Federation;
2 Faculty of Pharmacy, "Victor Babes" University of Medicine and Pharmacy, 2nd Eftimie Murgu Sq., Timisoara 300041, Romania
3 Res. Ctr. Pharmacotoxicol Evaluat, Faculty of Pharmacy, "Victor Babes" University of Medicine and Pharmacy Timisoara, 2nd Eftimie Murgu Sq., Timisoara 300041, Romania;
4 Res. Ctr. Drug Formulation and Technology, Faculty of Pharmacy, "Victor Babes" University of Medicine and Pharmacy Timisoara, 2nd Eftimie Murgu Sq., Timisoara 300041, Romania;
5 Faculty of Medicine, "Victor Babes" University of Medicine and Pharmacy, 2nd Eftimie Murgu Sq., Timisoara 300041, Romania

* Correspondence: obf@anrb.ru; adrian.voicu@umft.ro
# Authors with equal contribution jointly sharing the position as first author

## Abstract

A series of novel hybride chalcone *N*-ethyl-piperazinyl amide derivatives of oleanonic and ursonic acids were synthesized ad their antiproliferative potential was *in vitro* evaluated against the NCI-60 cancer cell line panel. Compounds 4 and 6 exhibited the highest overall antiproliferative activity with $GI_{50}$ values in some cases reaching nanomolar values. Thus, the two compounds were further assessed in detail in order to identify a possible antiproliferative mechanism of action. DAPI staining revealed that both compounds induced nuclei condensation and overall cell morphological changes consistent with apoptotic cell death. rtPCR analysis showed that up-regulation of pro-apoptic Bak gene combined with the down-regulation of the pro-survival Bcl-XL and Bcl-2 genes caused altered ratios between the proapoptotic and antiapoptotic proteins' levels, leading to overall induced apoptosis. Molecular docking analysis revealed that both compounds exhibited high scores for Bcl-XL inhibition suggesting that compounds may induce apoptotic cell death through targeted anti-apoptotic protein inhibition, as well.

**Key words:** oleanonic acid, ursonic acid, arylidene, piperazine, anticancer activity, NCI-60, antiproliferative activity, CAM assay, rtPCR, molecular docking

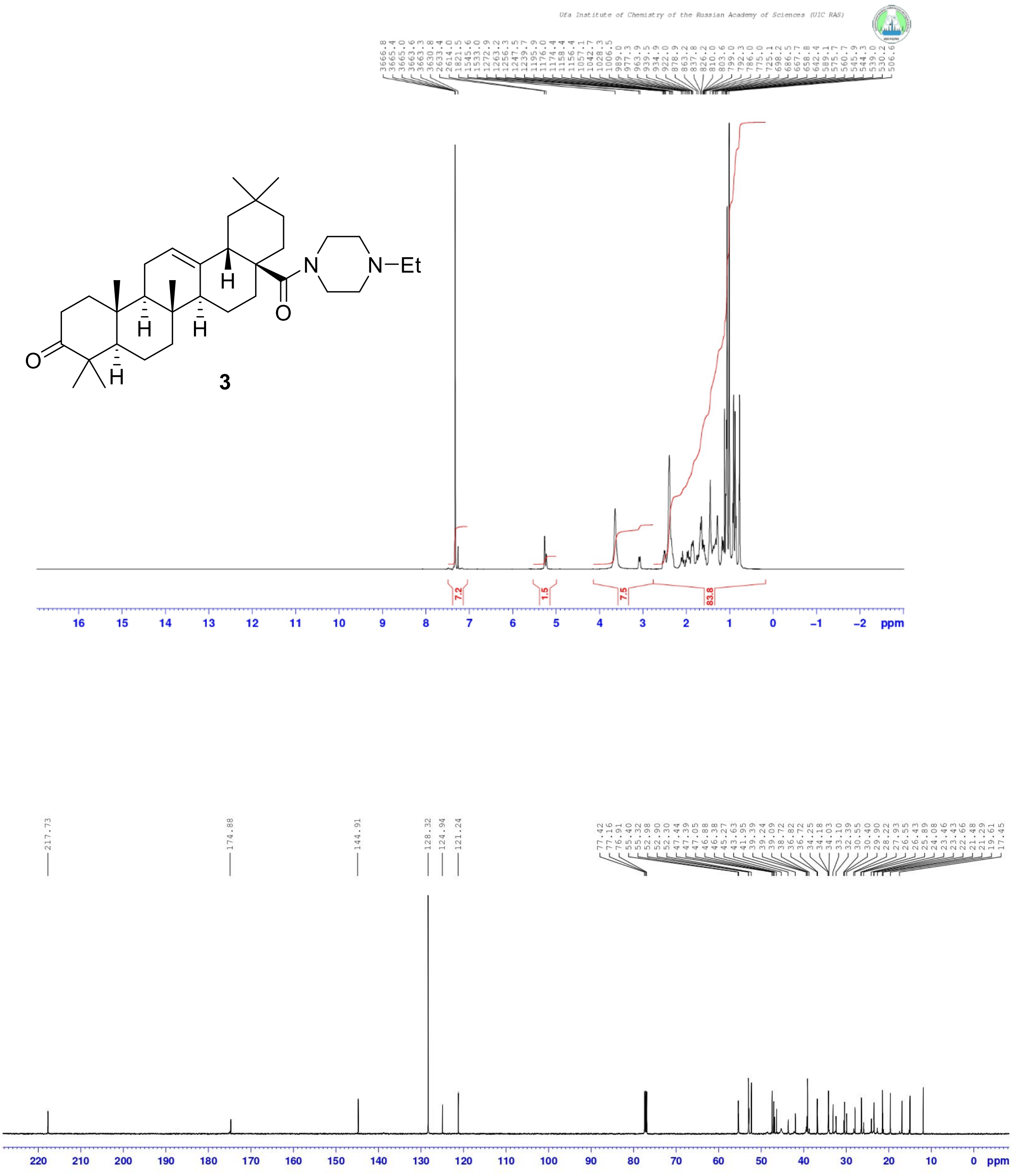


**Figure S1**. $^{1}$H NMR and $^{13}$C NMR spectra of compound **3**

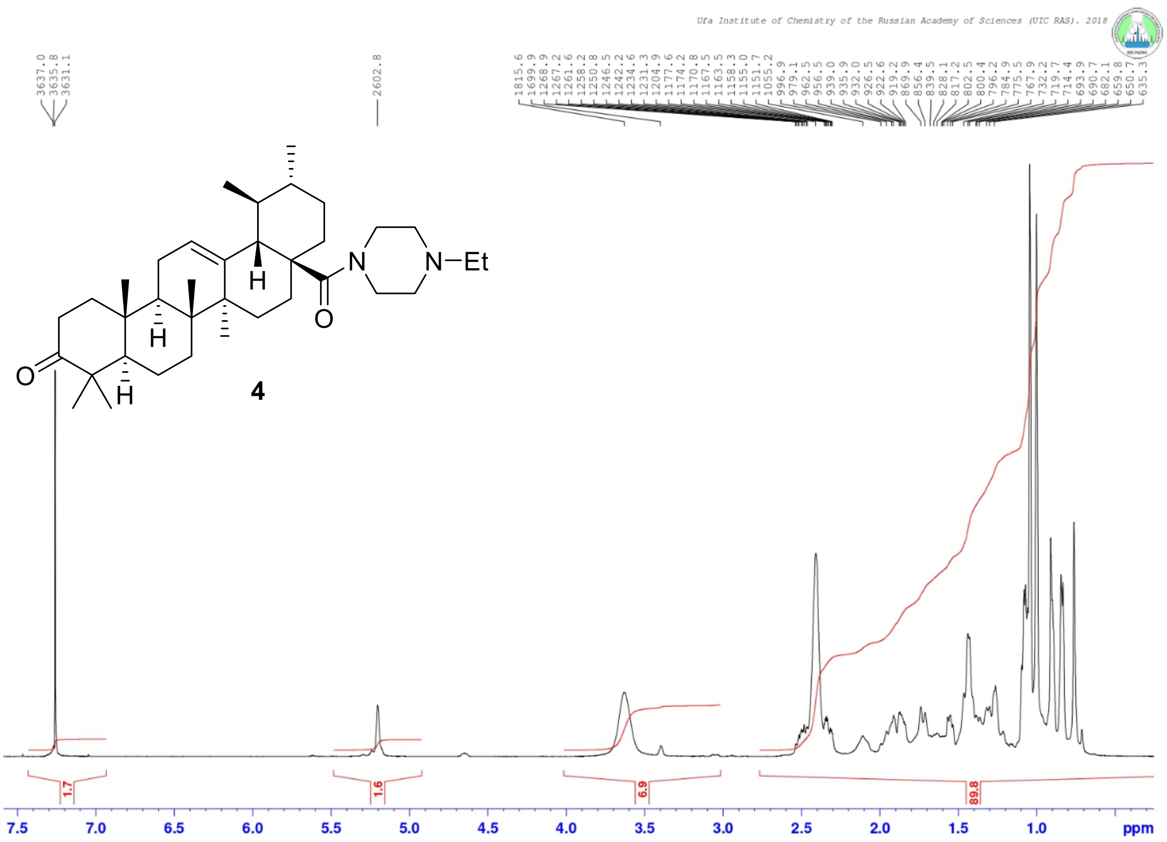
Ufa Institute of Chemistry of the Russian Academy of Sciences (UIC RAS). 2018
4
ppm

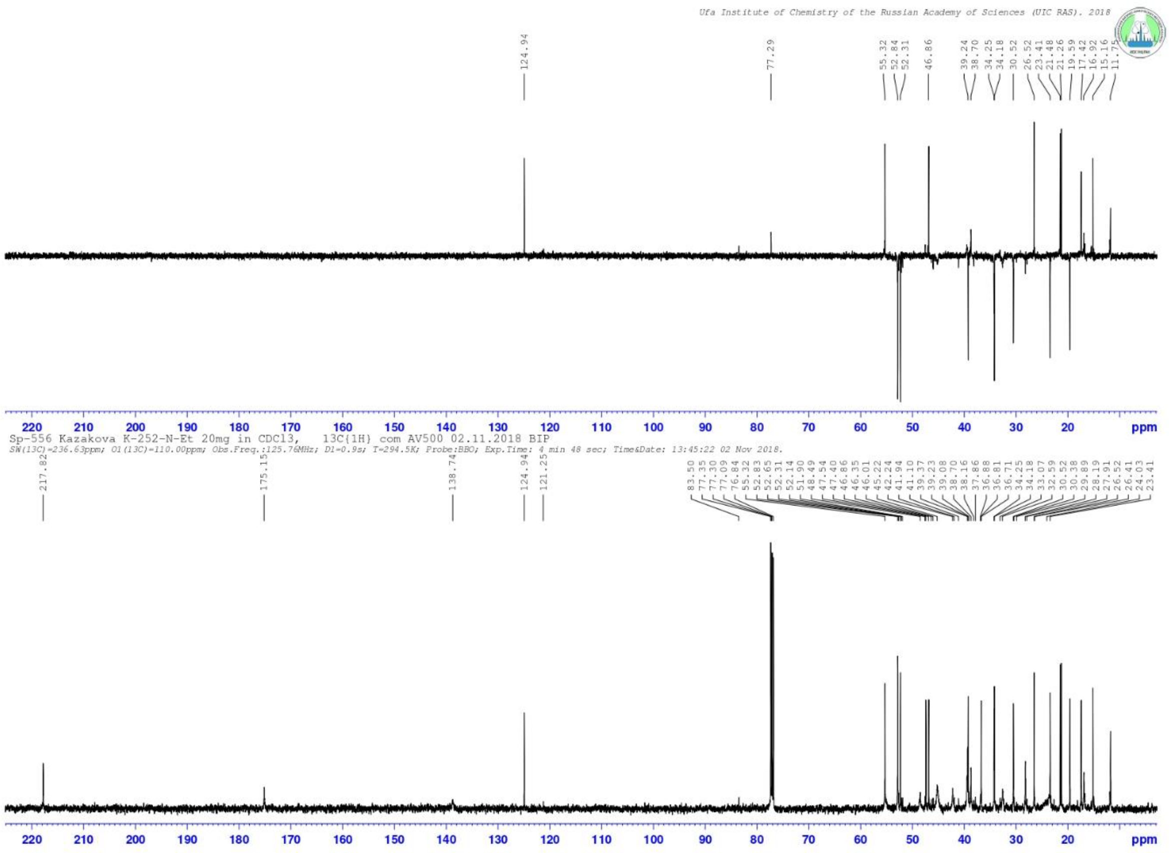

**Figure S2**. $^{1}H$ NMR and $^{13}C$ NMR spectra of compound **4**.

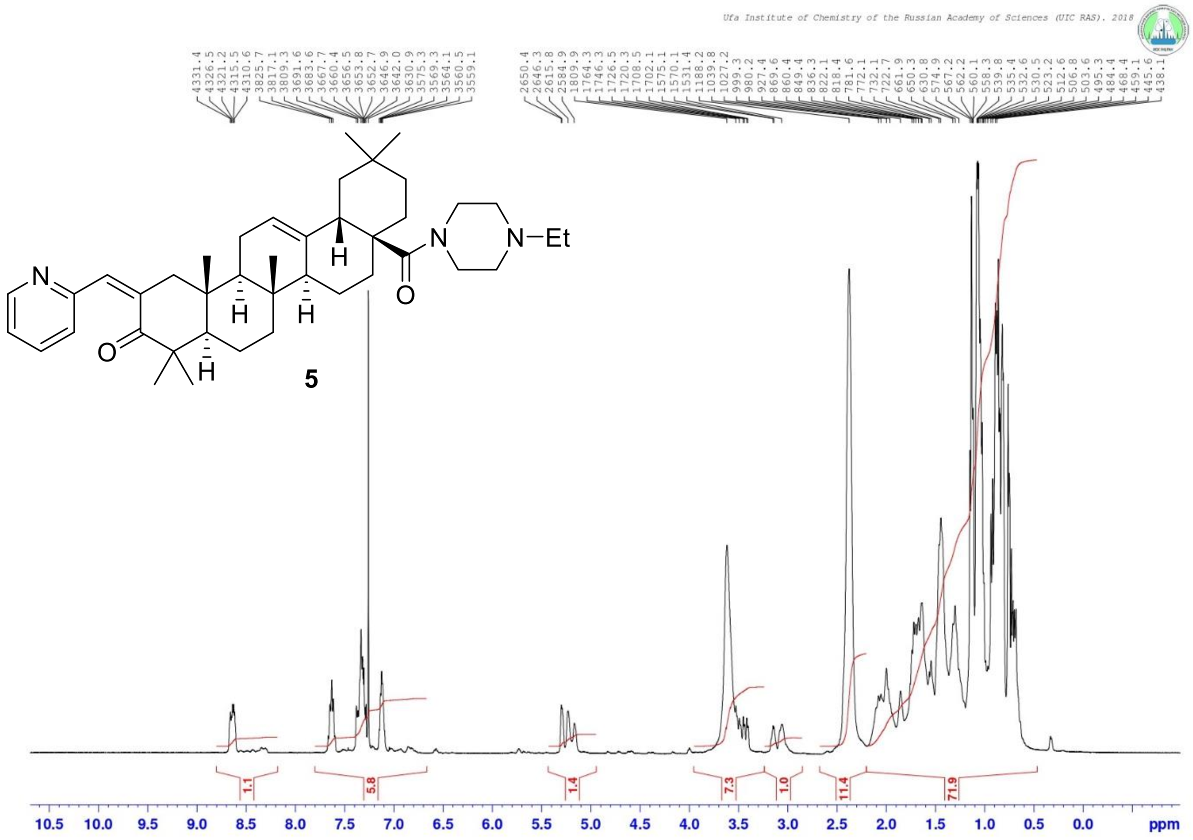


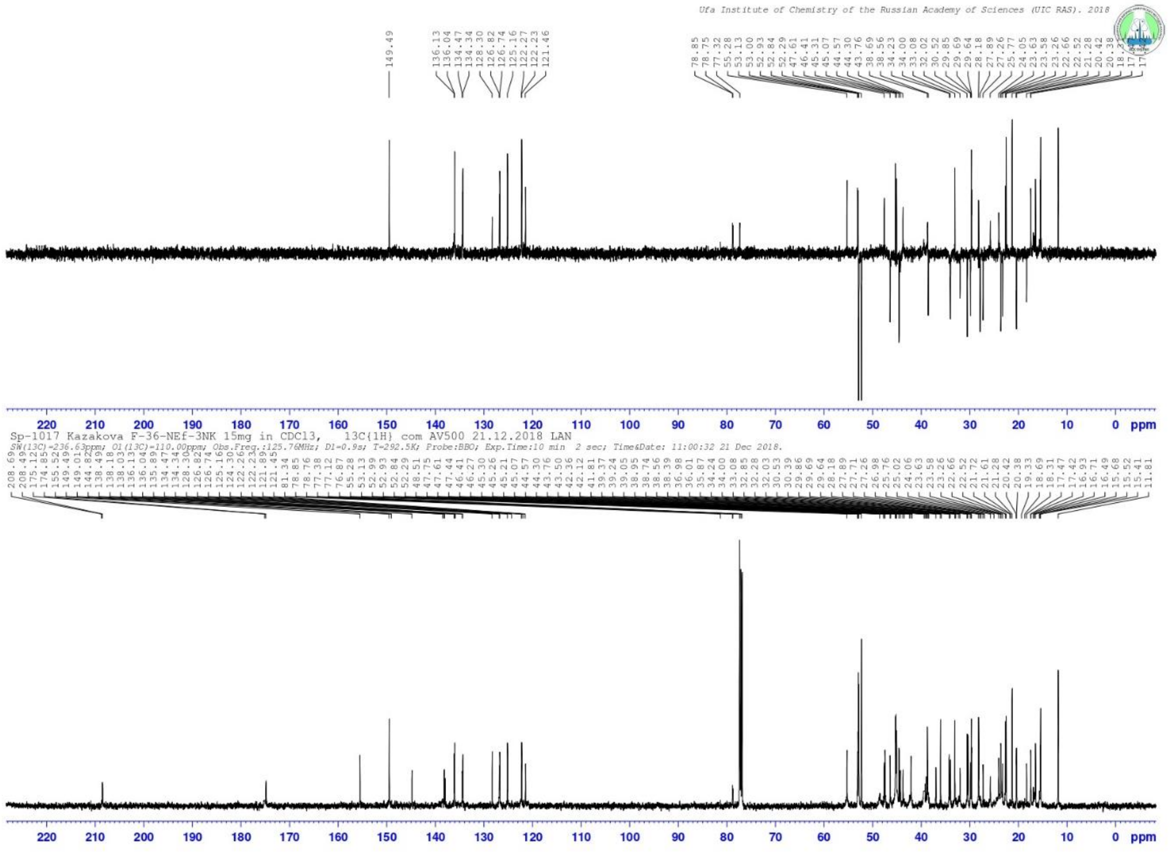


**Figure S3**. $^{1}$H NMR and $^{13}$C NMR spectra of compound **5**

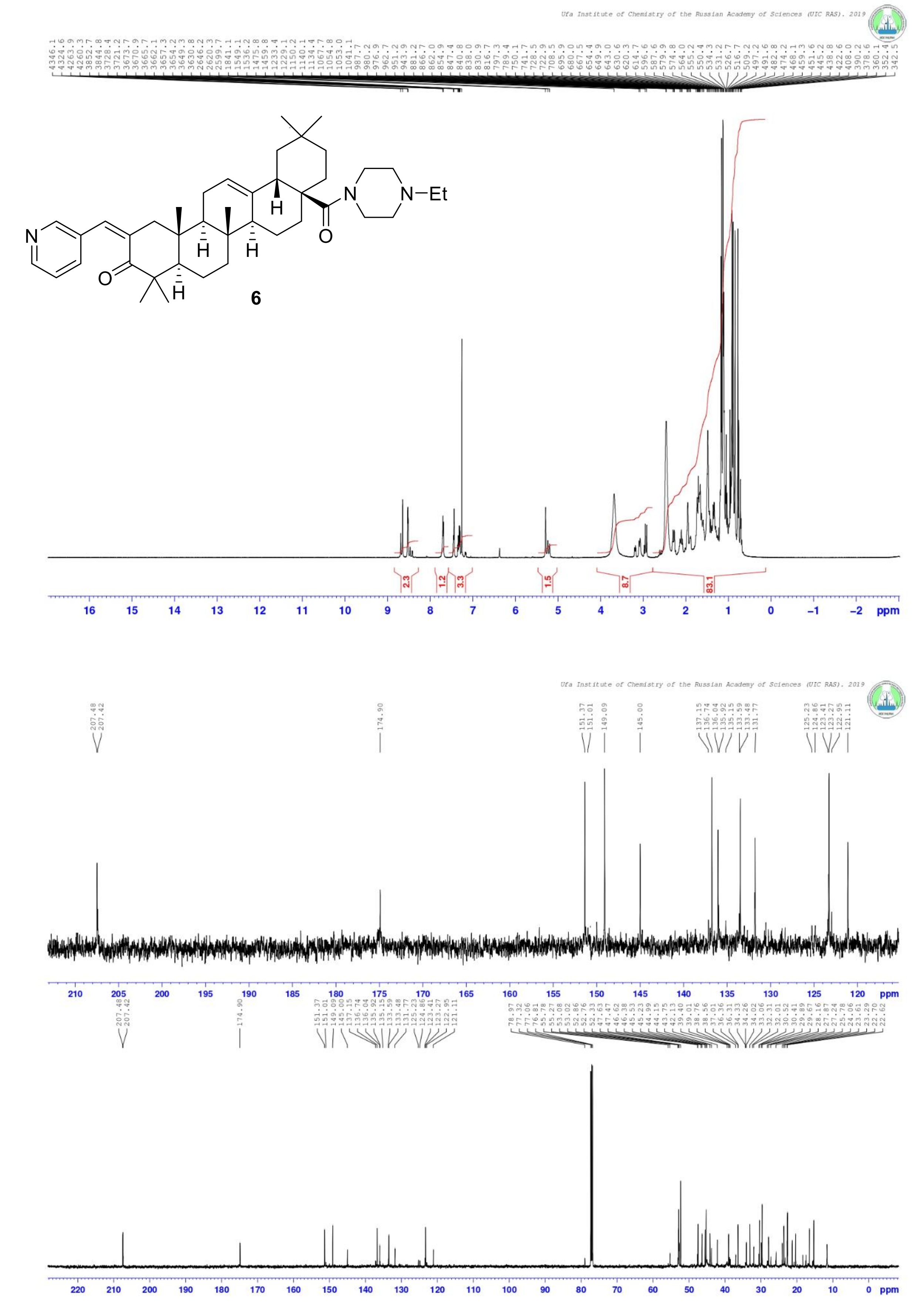


**Figure S4**. $^{1}$H NMR and $^{13}$C NMR spectra of compound **6**

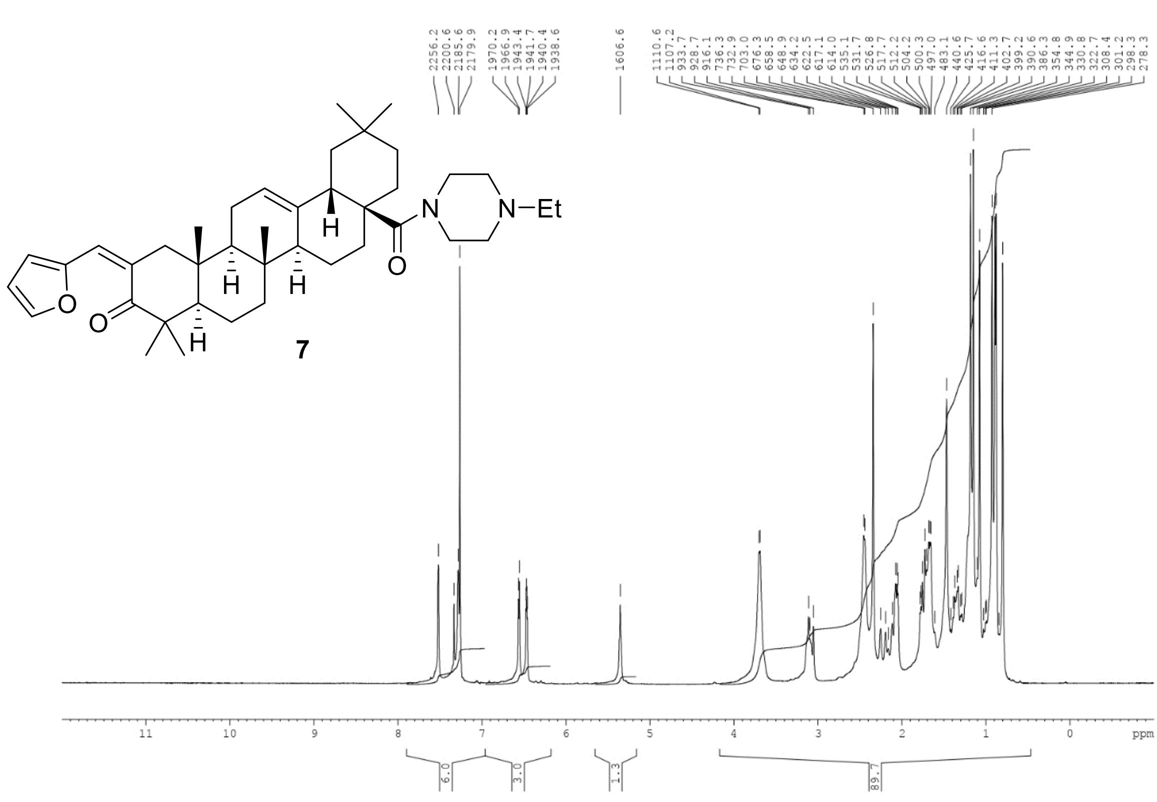

O
O
H
H
H
H
N
N−Et
O
2256.2
2200.6
2185.6
2179.9
1970.2
1966.9
1943.4
1941.7
1940.4
1938.6
1606.6
1110.6
1107.2
933.7
928.7
916.1
736.3
732.9
703.0
676.3
658.5
648.9
634.2
622.5
617.1
614.0
535.1
531.7
526.8
517.7
512.2
504.2
500.3
497.0
483.1
440.6
425.7
416.6
411.3
402.7
399.2
390.6
386.3
354.8
344.9
330.8
322.7
308.4
301.2
298.3
278.3
11
10
9
8
7
6
5
4
3
2
1
0
ppm
6.0
3.0
1.3
89.7


**7**

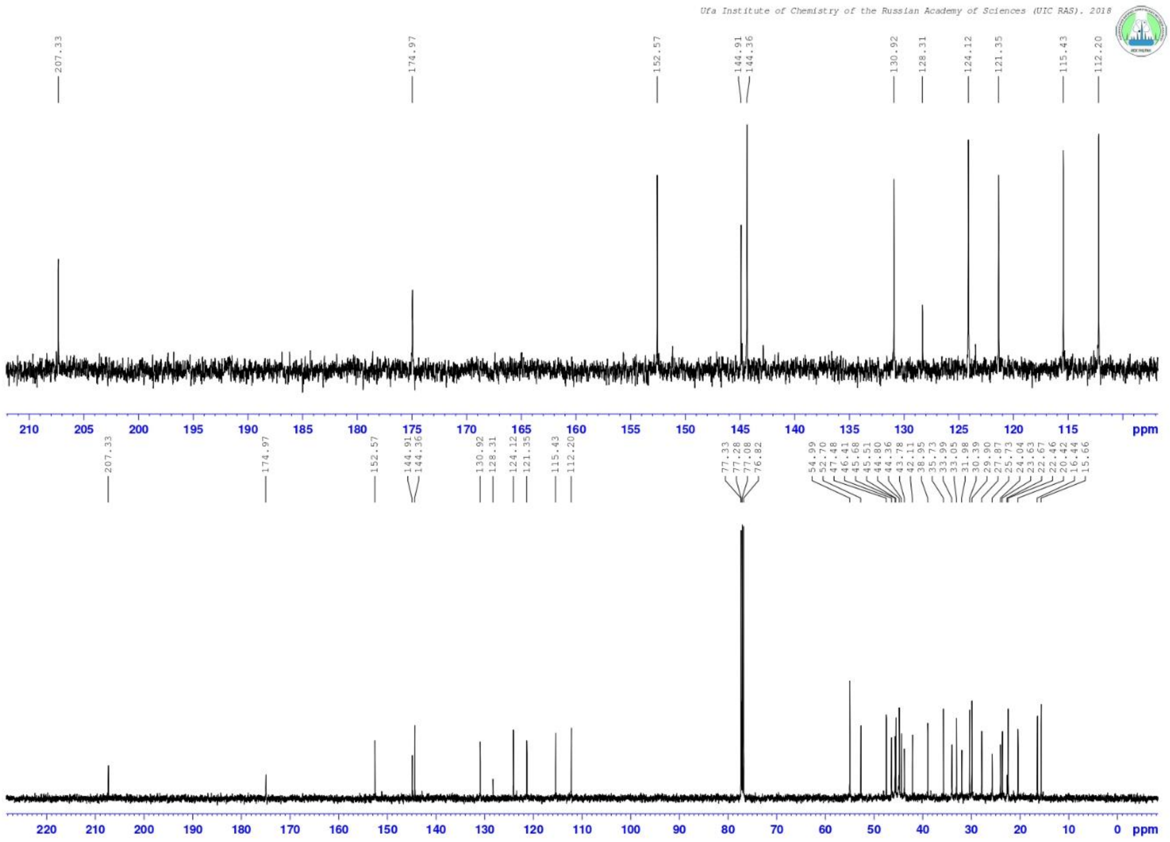


**Figure S5**. $^{1}$H NMR and $^{13}$C NMR spectra of compound **7**

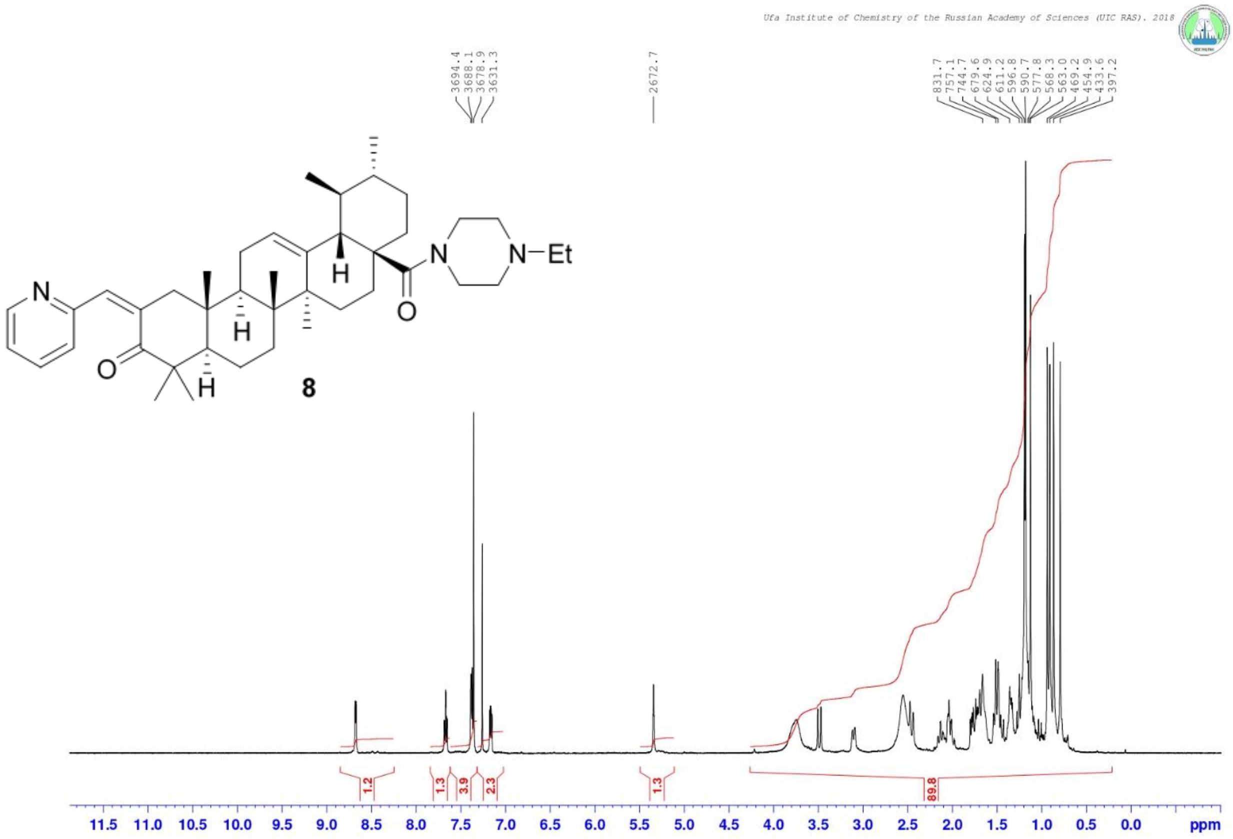

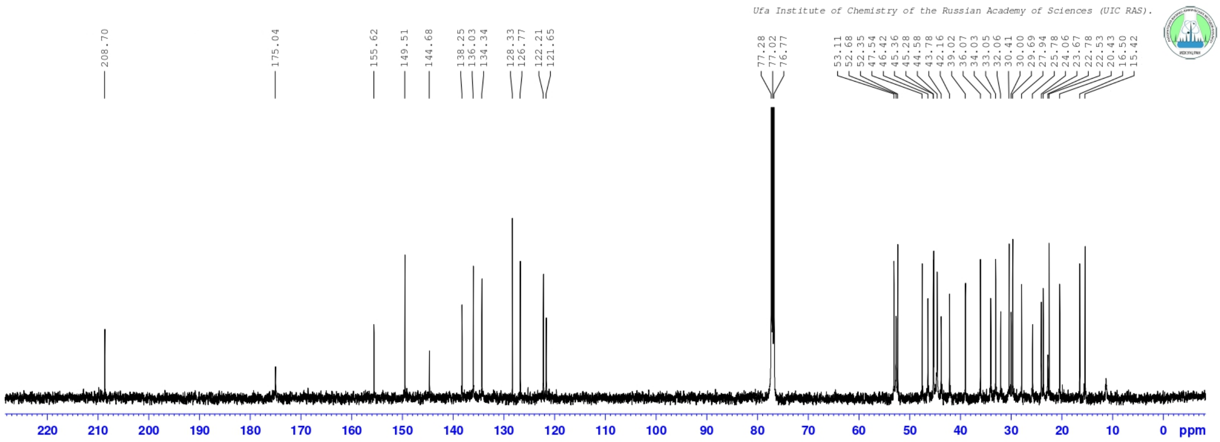

**Figure S6**. $^{1}$H NMR and $^{13}$C NMR spectra of compound **8**

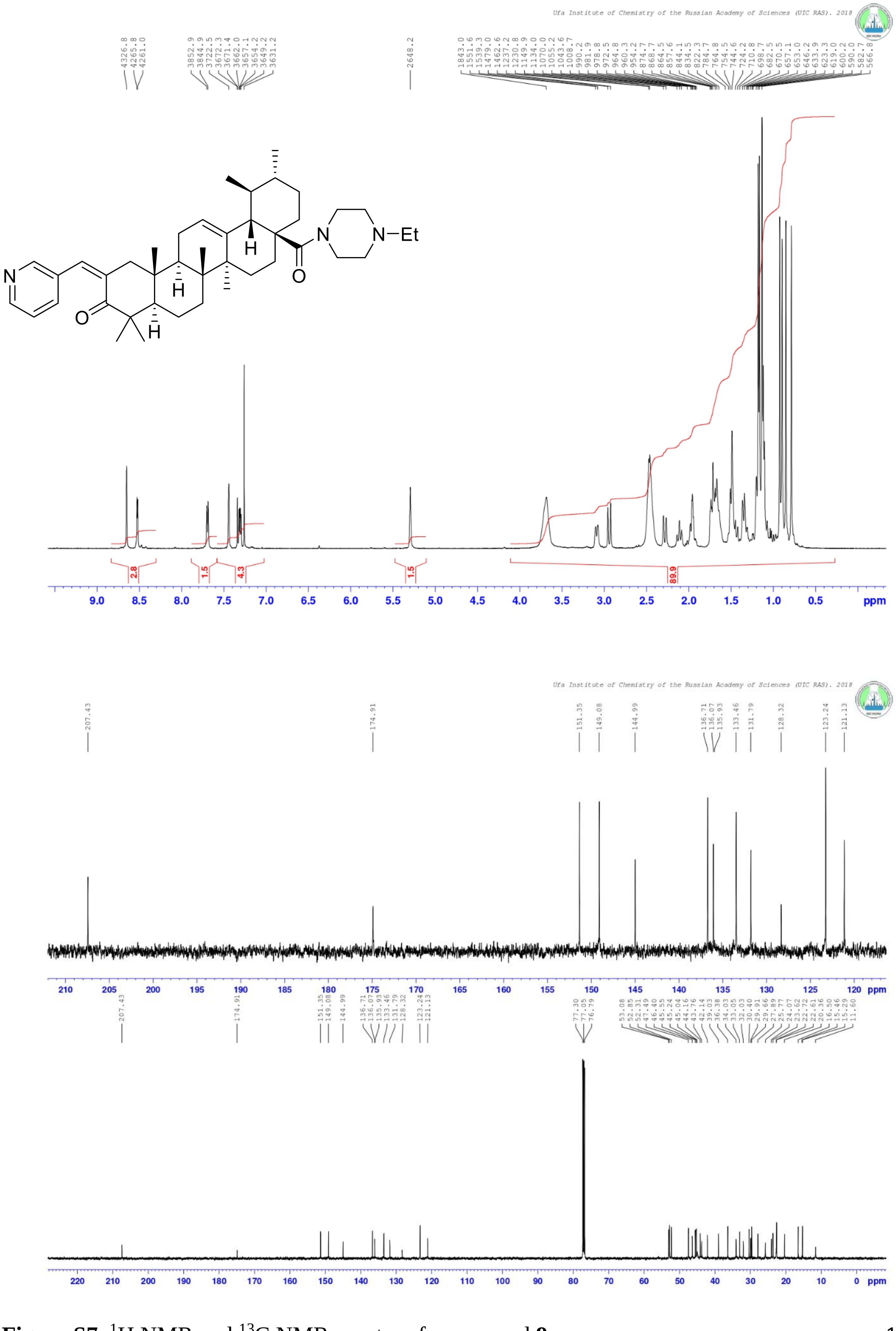


**Figure S7**. $^{1}$H NMR and $^{13}$C NMR spectra of compound **9**

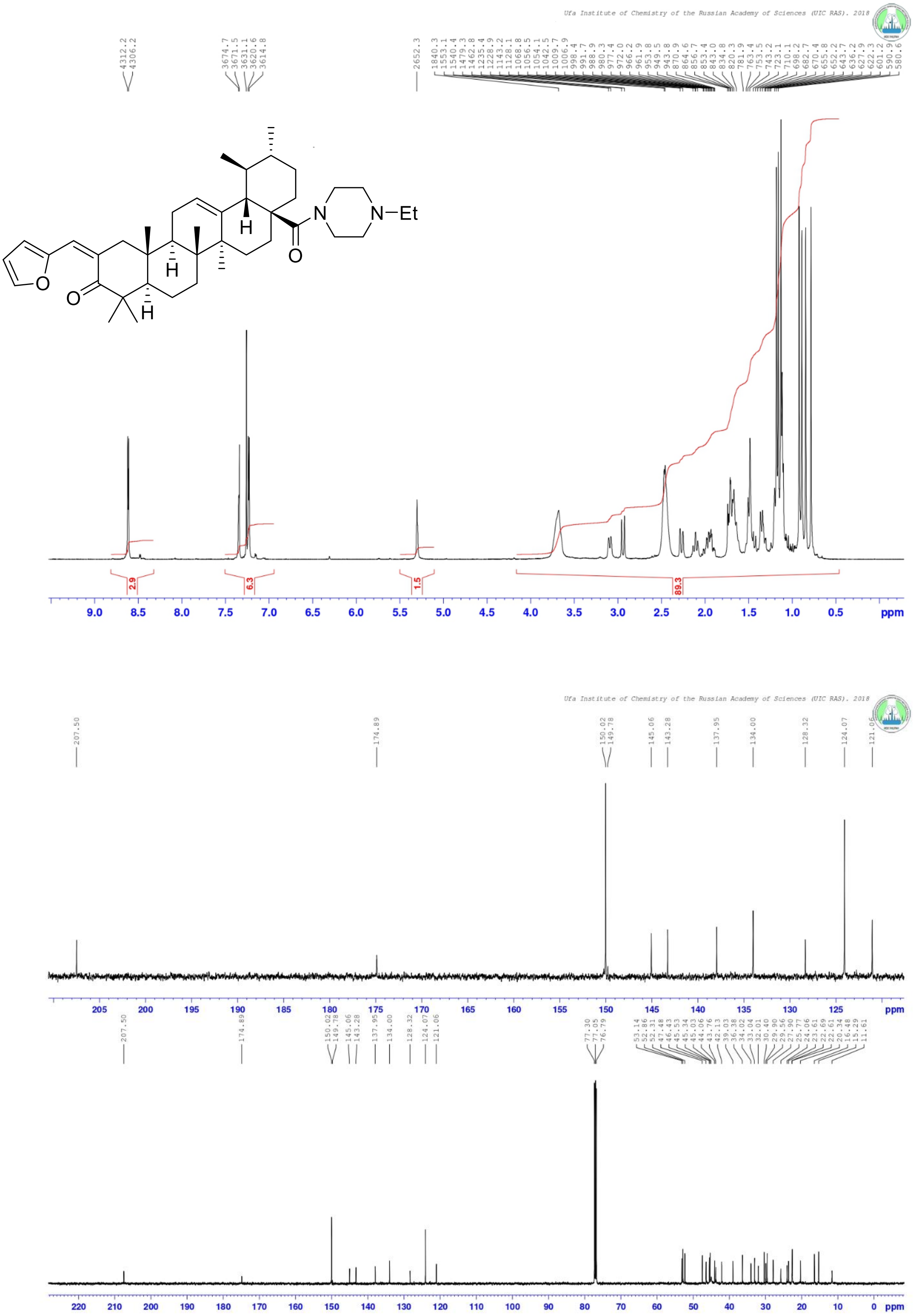


**Figure S8**. $^{1}H$ NMR and $^{13}C$ NMR spectra of compound **10**

| Developmental Therapeutics Program | NSC: D-811947 / 1 | Conc: 1.00E-5 Molar | Test Date: Jan 14, 2019 |
|---|---|---|---|
| One Dose Mean Graph | Experiment ID: 1901OS55 | | Report Date: Feb 24, 2021 |

| Panel/Cell Line | Growth Percent |
|---|---|
| **Leukemia** | |
| CCRF-CEM | 37.47 |
| HL-60(TB) | -35.82 |
| K-562 | 21.11 |
| MOLT-4 | 10.12 |
| RPMI-8226 | 9.86 |
| SR | 12.15 |
| **Non-Small Cell Lung Cancer** | |
| A549/ATCC | 46.06 |
| EKVX | 63.34 |
| HOP-62 | 78.84 |
| HOP-92 | 44.94 |
| NCI-H226 | 70.97 |
| NCI-H23 | 64.95 |
| NCI-H322M | 81.23 |
| NCI-H460 | 25.54 |
| NCI-H522 | 44.74 |
| **Colon Cancer** | |
| COLO 205 | 44.16 |
| HCC-2998 | 62.76 |
| HCT-116 | 19.06 |
| HCT-15 | 33.56 |
| HT29 | 8.29 |
| KM12 | 41.24 |
| SW-620 | 31.83 |
| **CNS Cancer** | |
| SF-268 | 41.52 |
| SF-295 | 76.29 |
| SF-539 | 48.94 |
| SNB-19 | 71.11 |
| SNB-75 | 15.59 |
| U251 | 46.77 |
| **Melanoma** | |
| LOX IMVI | 43.82 |
| MALME-3M | 40.53 |
| M14 | 57.06 |
| MDA-MB-435 | 33.87 |
| SK-MEL-2 | 77.05 |
| SK-MEL-28 | 64.26 |
| SK-MEL-5 | 49.22 |
| UACC-257 | 32.83 |
| UACC-62 | 47.34 |
| **Ovarian Cancer** | |
| IGROV1 | 65.02 |
| OVCAR-3 | 56.42 |
| OVCAR-4 | 50.71 |
| OVCAR-5 | 72.17 |
| OVCAR-8 | 63.00 |
| NCI/ADR-RES | 39.67 |
| SK-OV-3 | 70.14 |
| **Renal Cancer** | |
| 786-0 | 50.49 |
| A498 | -7.95 |
| ACHN | 65.74 |
| CAKI-1 | 60.01 |
| RXF 393 | 60.97 |
| SN12C | 51.84 |
| TK-10 | 80.36 |
| UO-31 | 34.08 |
| **Prostate Cancer** | |
| PC-3 | 26.09 |
| DU-145 | 76.63 |
| **Breast Cancer** | |
| MCF7 | 39.89 |
| MDA-MB-231/ATCC | 56.54 |
| HS 578T | 40.73 |
| BT-549 | 58.56 |
| T-47D | 56.39 |
| MDA-MB-468 | 30.47 |
| Mean | 46.01 |
| Delta | 81.83 |
| Range | 117.05 |

**Mean Growth Percent - Growth Percent**

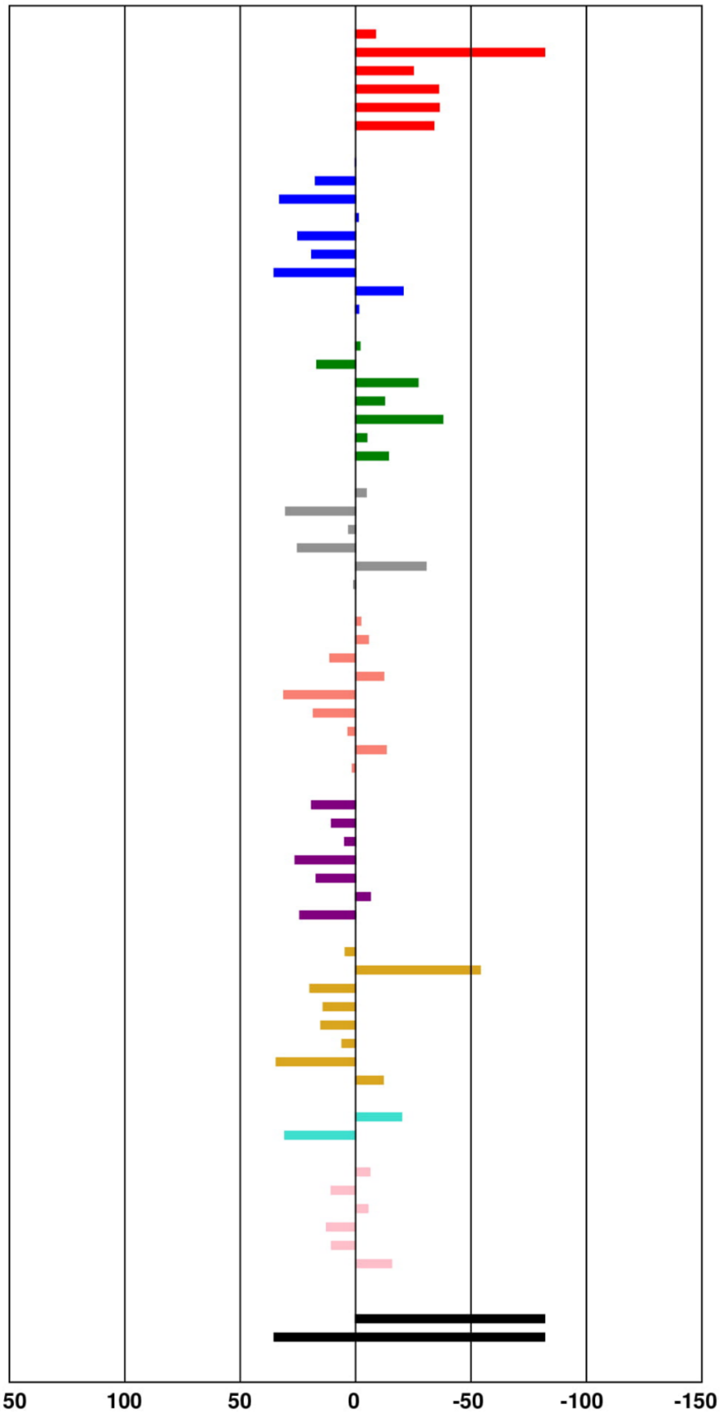


**Figure S9**. NCI-60 one dose data for compound **3**

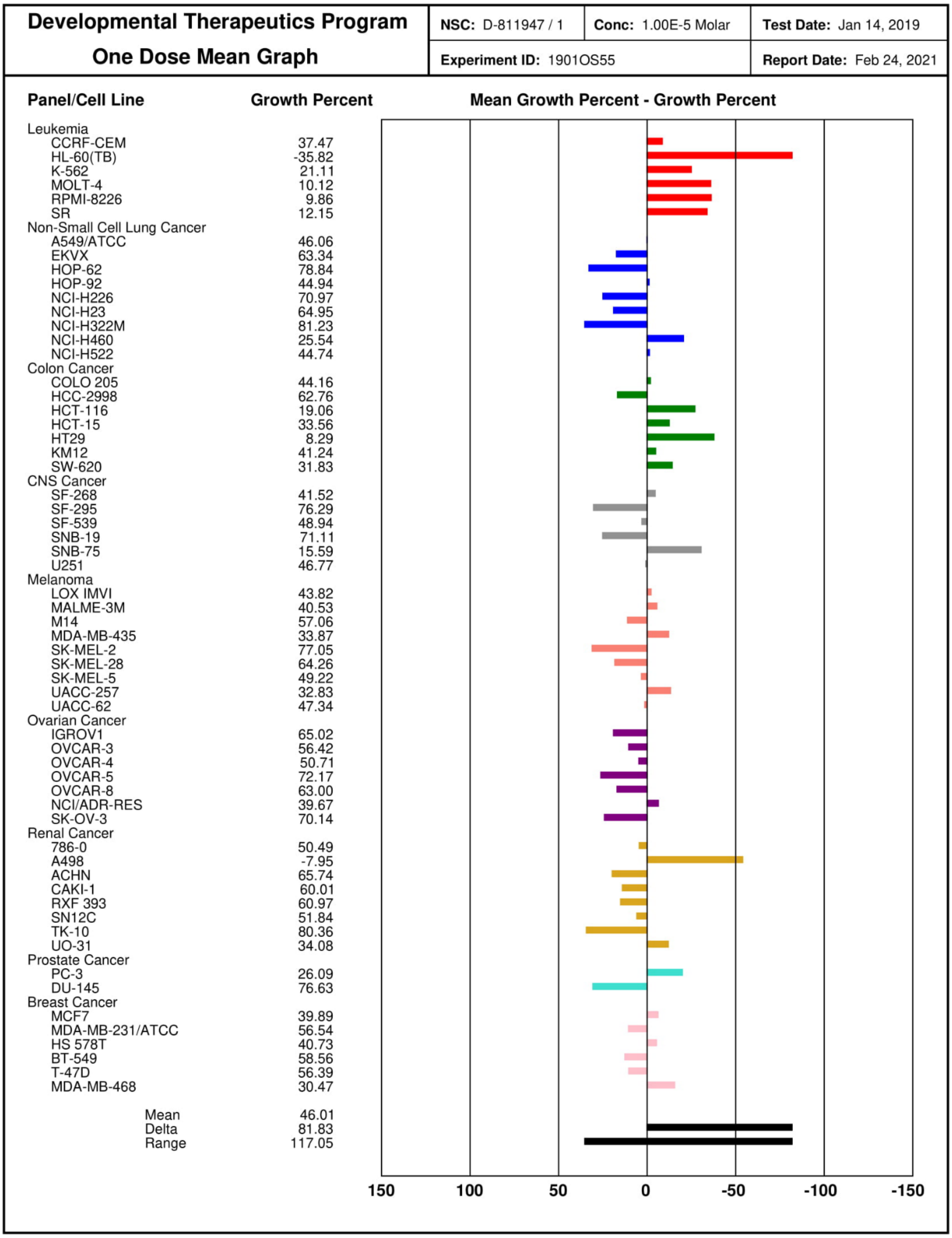


**Figure S10**. NCI-60 one dose data for compound **4**

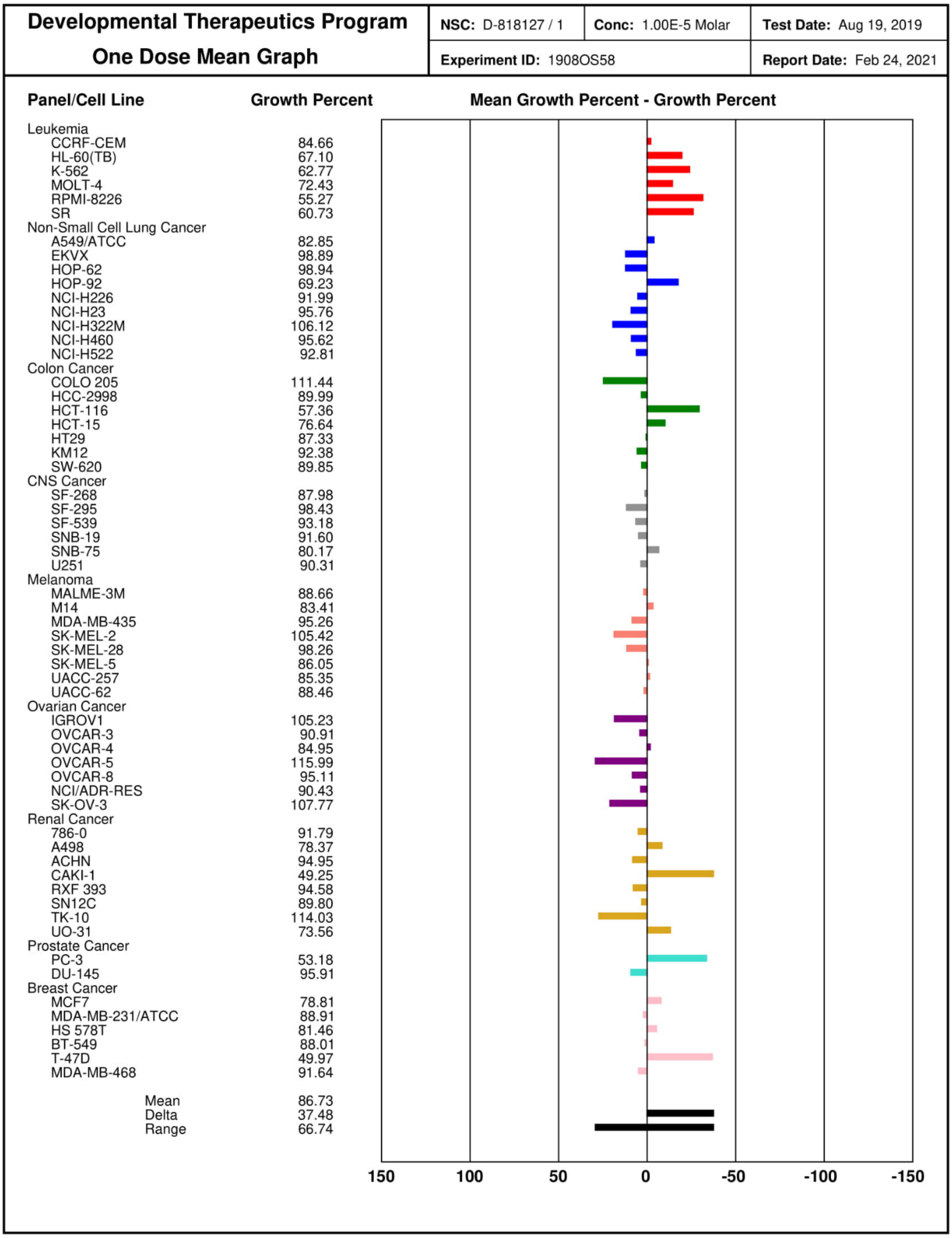


**Figure S11**. NCI-60 one dose data for compound **5**

**Developmental Therapeutics Program**

**One Dose Mean Graph**

| | |
|---|---|
| **NSC:** D-818136 / 1 | **Conc:** 1.00E-5 Molar |
| **Test Date:** Aug 19, 2019 | **Experiment ID:** 1908OS58 |
| **Report Date:** Feb 24, 2021 | |

| Panel/Cell Line | Growth Percent |
|---|---|
| Leukemia | |
| CCRF-CEM | 33.39 |
| HL-60(TB) | -37.33 |
| K-562 | 6.69 |
| MOLT-4 | 4.16 |
| RPMI-8226 | 5.88 |
| SR | 4.78 |
| Non-Small Cell Lung Cancer | |
| A549/ATCC | 13.53 |
| EKVX | 25.37 |
| HOP-62 | 44.50 |
| HOP-92 | 9.80 |
| NCI-H226 | 32.91 |
| NCI-H23 | 27.11 |
| NCI-H322M | 62.29 |
| NCI-H460 | 12.13 |
| NCI-H522 | 28.39 |
| Colon Cancer | |
| COLO 205 | 25.88 |
| HCC-2998 | 18.93 |
| HCT-116 | 13.26 |
| HCT-15 | 13.67 |
| HT29 | 10.57 |
| KM12 | 33.84 |
| SW-620 | 27.56 |
| CNS Cancer | |
| SF-268 | 37.58 |
| SF-295 | 16.67 |
| SF-539 | 41.51 |
| SNB-19 | 48.31 |
| SNB-75 | 45.27 |
| U251 | -88.15 |
| Melanoma | |
| MALME-3M | 30.35 |
| M14 | 34.43 |
| MDA-MB-435 | 34.89 |
| SK-MEL-2 | 41.94 |
| SK-MEL-28 | 41.74 |
| SK-MEL-5 | -91.03 |
| UACC-257 | 3.05 |
| UACC-62 | 26.64 |
| Ovarian Cancer | |
| IGROV1 | 51.65 |
| OVCAR-3 | 28.60 |
| OVCAR-4 | 7.28 |
| OVCAR-5 | 83.75 |
| OVCAR-8 | 27.08 |
| NCI/ADR-RES | 8.32 |
| SK-OV-3 | 56.02 |
| Renal Cancer | |
| 786-0 | 50.20 |
| A498 | 45.09 |
| ACHN | 33.36 |
| CAKI-1 | 31.48 |
| RXF 393 | -7.84 |
| SN12C | 29.52 |
| TK-10 | 52.38 |
| UO-31 | 33.33 |
| Prostate Cancer | |
| PC-3 | 17.23 |
| DU-145 | 50.03 |
| Breast Cancer | |
| MCF7 | 5.68 |
| MDA-MB-231/ATCC | 40.66 |
| HS 578T | 54.58 |
| BT-549 | 25.73 |
| T-47D | 20.13 |
| MDA-MB-468 | -6.42 |
| Mean | 23.36 |
| Delta | 114.39 |
| Range | 174.78 |

**Mean Growth Percent - Growth Percent**

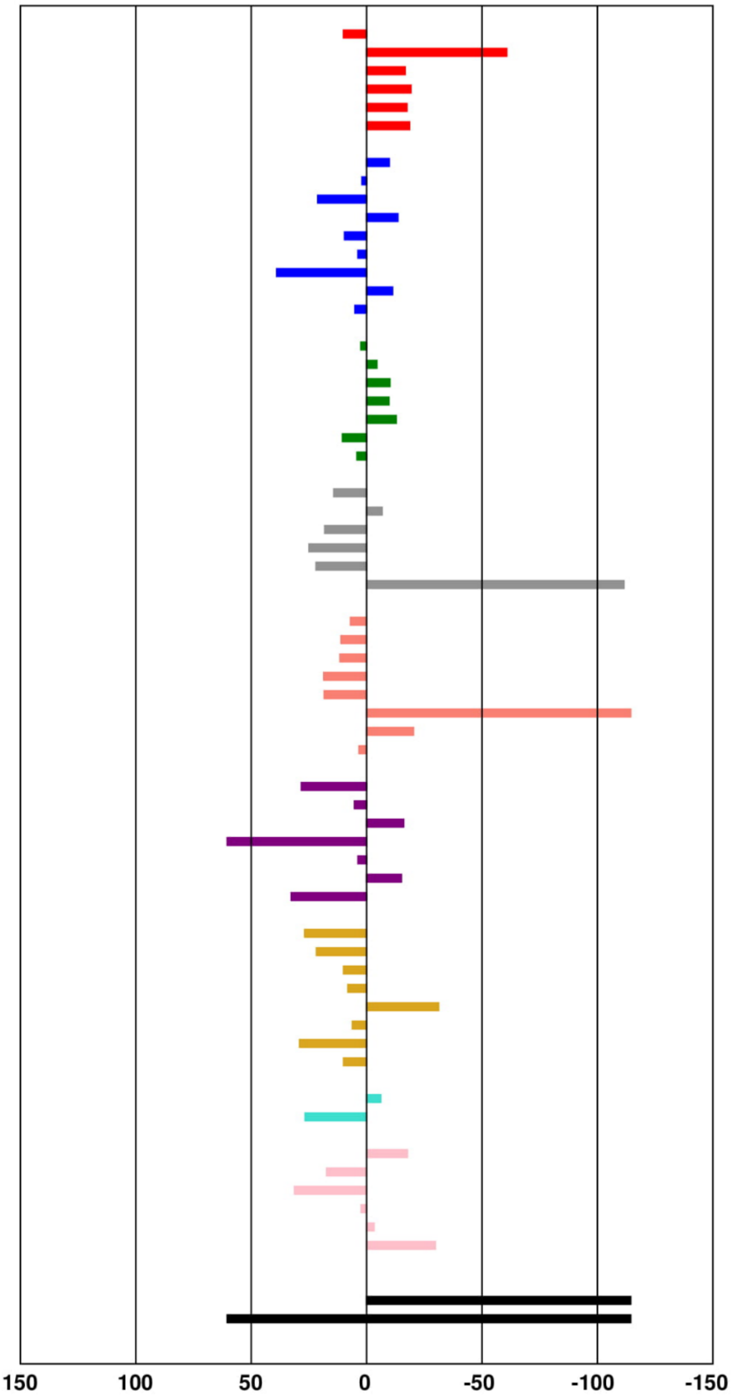


**Figure S12**. NCI-60 one dose data for compound **6**

**Developmental Therapeutics Program**

**One Dose Mean Graph**

| **NSC:** D-818138 / 1 | **Conc:** 1.00E-5 Molar | **Test Date:** Aug 19, 2019 |
|---|---|---|
| **Experiment ID:** 1908OS58 | | **Report Date:** Feb 24, 2021 |

| Panel/Cell Line | Growth Percent |
|---|---|
| Leukemia | |
| CCRF-CEM | 75.19 |
| HL-60(TB) | 16.53 |
| K-562 | 68.22 |
| MOLT-4 | 41.38 |
| RPMI-8226 | 48.45 |
| SR | 50.27 |
| Non-Small Cell Lung Cancer | |
| A549/ATCC | 80.03 |
| EKVX | 84.81 |
| HOP-62 | 95.41 |
| HOP-92 | 51.83 |
| NCI-H226 | 89.19 |
| NCI-H23 | 95.02 |
| NCI-H322M | 96.44 |
| NCI-H460 | 90.17 |
| NCI-H522 | 86.96 |
| Colon Cancer | |
| COLO 205 | 109.05 |
| HCC-2998 | 86.61 |
| HCT-116 | 54.29 |
| HCT-15 | 57.64 |
| HT29 | 68.36 |
| KM12 | 92.26 |
| SW-620 | 83.23 |
| CNS Cancer | |
| SF-268 | 58.09 |
| SF-295 | 97.52 |
| SF-539 | 88.66 |
| SNB-19 | 89.60 |
| SNB-75 | 56.76 |
| U251 | 69.01 |
| Melanoma | |
| MALME-3M | 92.42 |
| M14 | 84.51 |
| MDA-MB-435 | 83.16 |
| SK-MEL-2 | 100.99 |
| SK-MEL-28 | 89.43 |
| SK-MEL-5 | 92.64 |
| UACC-257 | 82.95 |
| UACC-62 | 88.55 |
| Ovarian Cancer | |
| IGROV1 | 98.26 |
| OVCAR-3 | 92.21 |
| OVCAR-4 | 73.40 |
| OVCAR-5 | 103.10 |
| OVCAR-8 | 92.50 |
| NCI/ADR-RES | 65.60 |
| SK-OV-3 | 109.58 |
| Renal Cancer | |
| 786-0 | 72.84 |
| A498 | 80.38 |
| ACHN | 86.04 |
| CAKI-1 | 46.49 |
| RXF 393 | 73.02 |
| SN12C | 72.44 |
| TK-10 | 104.36 |
| UO-31 | 73.46 |
| Prostate Cancer | |
| PC-3 | 47.85 |
| DU-145 | 86.77 |
| Breast Cancer | |
| MCF7 | 79.60 |
| MDA-MB-231/ATCC | 75.96 |
| HS 578T | 84.52 |
| BT-549 | 80.12 |
| T-47D | 64.05 |
| MDA-MB-468 | 66.11 |
| Mean | 78.38 |
| Delta | 61.85 |
| Range | 93.05 |

**Mean Growth Percent - Growth Percent**

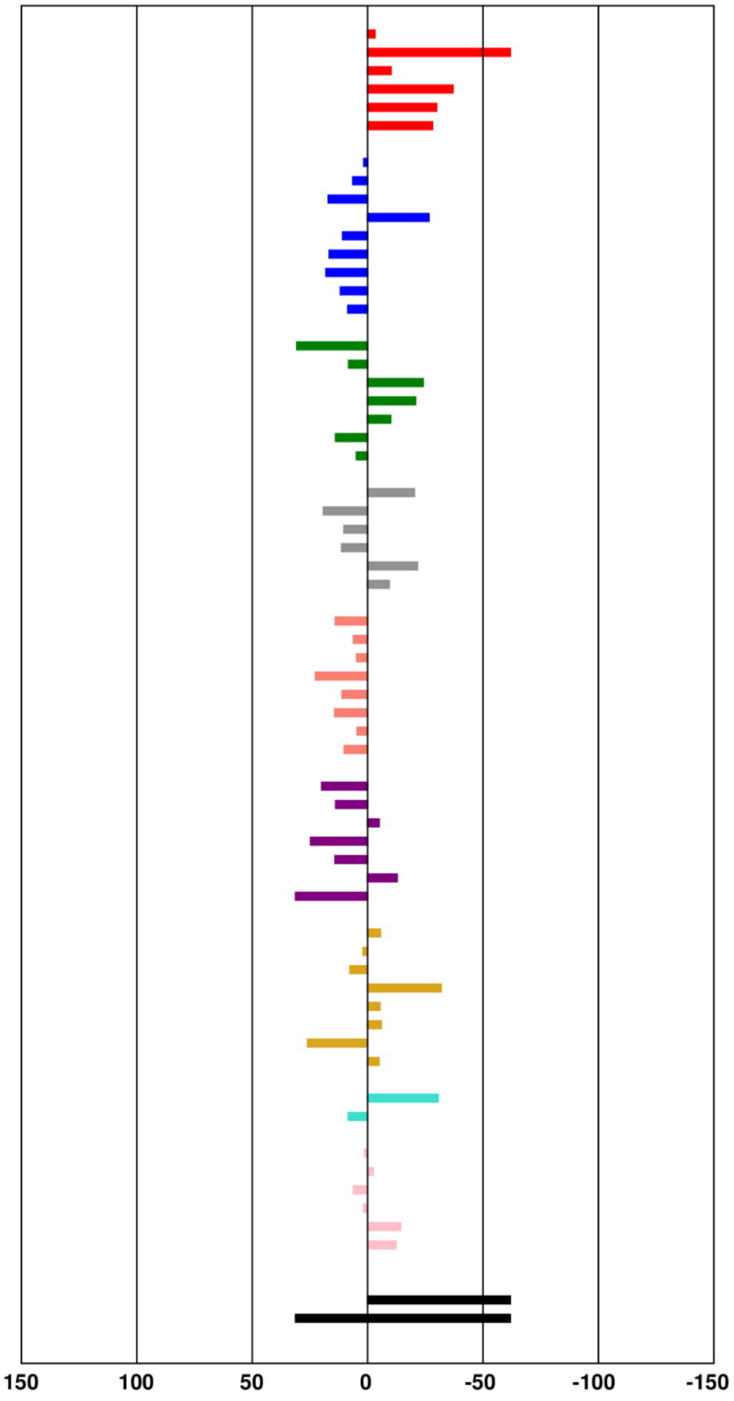


**Figure S13**. NCI-60 one dose data for compound **7**

| Developmental Therapeutics Program | NSC: D-811948 / 1 | Conc: 1.00E-5 Molar | Test Date: Jan 14, 2019 |
|---|---|---|---|
| One Dose Mean Graph | Experiment ID: 1901OS55 | | Report Date: Feb 24, 2021 |

| Panel/Cell Line | Growth Percent |
|---|---|
| Leukemia | |
| CCRF-CEM | 93.64 |
| HL-60(TB) | 86.45 |
| K-562 | 92.34 |
| MOLT-4 | 74.76 |
| RPMI-8226 | 83.71 |
| SR | 76.91 |
| Non-Small Cell Lung Cancer | |
| A549/ATCC | 94.82 |
| EKVX | 95.23 |
| HOP-62 | 99.71 |
| HOP-92 | 83.39 |
| NCI-H226 | 95.74 |
| NCI-H23 | 106.02 |
| NCI-H322M | 93.15 |
| NCI-H460 | 91.66 |
| NCI-H522 | 86.79 |
| Colon Cancer | |
| COLO 205 | 98.08 |
| HCC-2998 | 104.53 |
| HCT-116 | 86.78 |
| HCT-15 | 93.71 |
| HT29 | 97.20 |
| KM12 | 99.66 |
| SW-620 | 96.71 |
| CNS Cancer | |
| SF-268 | 91.03 |
| SF-295 | 102.31 |
| SF-539 | 97.04 |
| SNB-19 | 89.74 |
| SNB-75 | 81.82 |
| U251 | 94.95 |
| Melanoma | |
| LOX IMVI | 96.20 |
| MALME-3M | 83.39 |
| M14 | 101.17 |
| MDA-MB-435 | 92.60 |
| SK-MEL-2 | 103.70 |
| SK-MEL-28 | 99.53 |
| SK-MEL-5 | 97.89 |
| UACC-257 | 92.30 |
| UACC-62 | 90.99 |
| Ovarian Cancer | |
| IGROV1 | 94.75 |
| OVCAR-3 | 93.95 |
| OVCAR-4 | 94.55 |
| OVCAR-5 | 102.25 |
| OVCAR-8 | 94.88 |
| NCI/ADR-RES | 102.85 |
| SK-OV-3 | 97.48 |
| Renal Cancer | |
| 786-0 | 96.42 |
| A498 | 79.14 |
| ACHN | 95.14 |
| CAKI-1 | 76.48 |
| RXF 393 | 99.62 |
| SN12C | 91.98 |
| TK-10 | 99.13 |
| UO-31 | 72.90 |
| Prostate Cancer | |
| PC-3 | 76.95 |
| DU-145 | 96.14 |
| Breast Cancer | |
| MCF7 | 78.78 |
| MDA-MB-231/ATCC | 86.84 |
| HS 578T | 89.13 |
| BT-549 | 107.55 |
| T-47D | 70.92 |
| MDA-MB-468 | 98.42 |
| Mean | 92.36 |
| Delta | 21.44 |
| Range | 36.63 |

Mean Growth Percent - Growth Percent

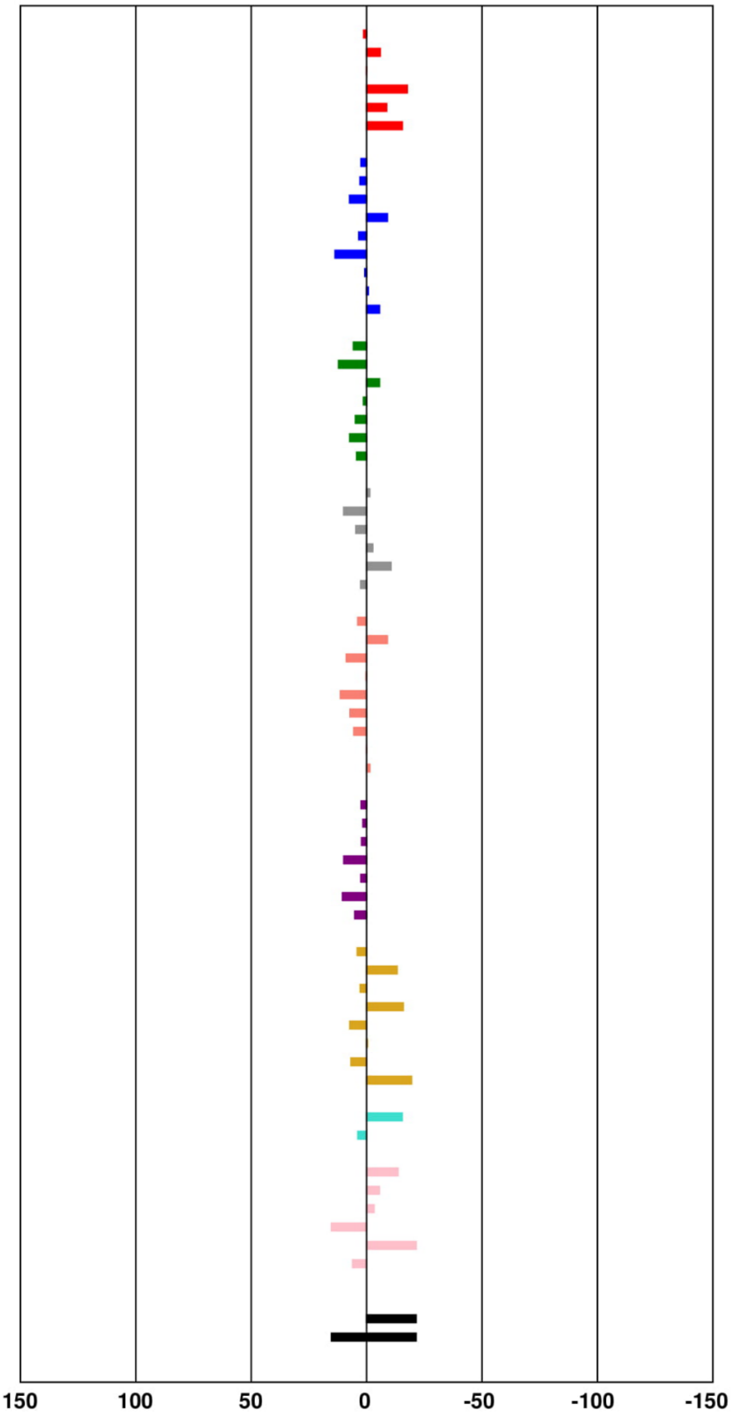


**Figure S14**. NCI-60 one dose data for compound **8**

| Developmental Therapeutics Program | NSC: D-811949 / 1 | Conc: 1.00E-5 Molar | Test Date: Jan 14, 2019 |
|---|---|---|---|
| One Dose Mean Graph | Experiment ID: 1901OS55 | | Report Date: Feb 24, 2021 |

| Panel/Cell Line | Growth Percent |
|---|---|
| Leukemia | |
| CCRF-CEM | 8.77 |
| HL-60(TB) | -38.20 |
| K-562 | 6.47 |
| MOLT-4 | 2.70 |
| RPMI-8226 | -15.25 |
| SR | -4.44 |
| Non-Small Cell Lung Cancer | |
| A549/ATCC | 32.03 |
| EKVX | 41.49 |
| HOP-62 | 44.45 |
| HOP-92 | 56.94 |
| NCI-H226 | 47.84 |
| NCI-H23 | 34.16 |
| NCI-H322M | 65.71 |
| NCI-H460 | 7.51 |
| NCI-H522 | 17.70 |
| Colon Cancer | |
| COLO 205 | 11.51 |
| HCC-2998 | 19.58 |
| HCT-116 | 10.31 |
| HCT-15 | 15.22 |
| HT29 | 12.12 |
| KM12 | 18.17 |
| SW-620 | 13.30 |
| CNS Cancer | |
| SF-268 | 51.01 |
| SF-295 | 37.29 |
| SF-539 | 8.77 |
| SNB-19 | 45.93 |
| SNB-75 | 27.63 |
| U251 | 27.94 |
| Melanoma | |
| LOX IMVI | 14.91 |
| MALME-3M | 11.97 |
| M14 | 40.87 |
| MDA-MB-435 | 23.98 |
| SK-MEL-2 | 28.34 |
| SK-MEL-28 | 37.42 |
| SK-MEL-5 | -23.36 |
| UACC-257 | 25.97 |
| UACC-62 | 33.84 |
| Ovarian Cancer | |
| IGROV1 | 50.90 |
| OVCAR-3 | 25.86 |
| OVCAR-4 | 26.72 |
| OVCAR-5 | 56.74 |
| OVCAR-8 | 32.64 |
| NCI/ADR-RES | 26.72 |
| SK-OV-3 | 54.26 |
| Renal Cancer | |
| 786-0 | 42.38 |
| A498 | 43.80 |
| ACHN | 20.69 |
| CAKI-1 | 39.78 |
| RXF 393 | 3.08 |
| SN12C | 29.60 |
| TK-10 | 43.07 |
| UO-31 | 10.46 |
| Prostate Cancer | |
| PC-3 | 16.75 |
| DU-145 | 47.99 |
| Breast Cancer | |
| MCF7 | 8.54 |
| MDA-MB-231/ATCC | 34.75 |
| HS 578T | 25.18 |
| BT-549 | 41.17 |
| T-47D | 21.99 |
| MDA-MB-468 | 16.36 |
| Mean | 25.33 |
| Delta | 63.53 |
| Range | 103.91 |

Mean Growth Percent - Growth Percent

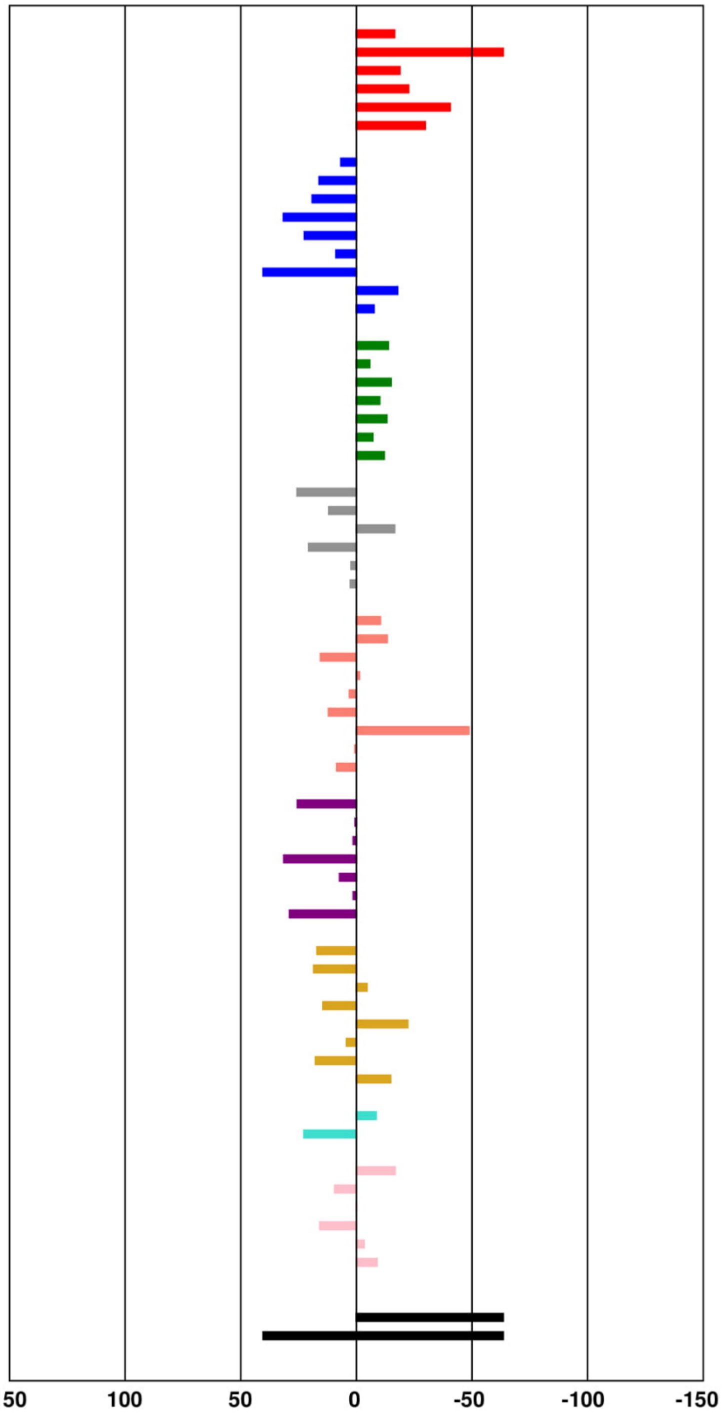


**Figure S15**. NCI-60 one dose data for compound **9**

**Developmental Therapeutics Program** | **NSC:** D-811951 / 1 | **Conc:** 1.00E-5 Molar | **Test Date:** Jan 14, 2019

**One Dose Mean Graph** | **Experiment ID:** 1901OS55 | **Report Date:** Feb 24, 2021

| Panel/Cell Line | Growth Percent |
|---|---|
| Leukemia | |
| CCRF-CEM | 68.24 |
| HL-60(TB) | 47.20 |
| K-562 | 47.62 |
| MOLT-4 | 57.10 |
| RPMI-8226 | 36.36 |
| SR | 31.17 |
| Non-Small Cell Lung Cancer | |
| A549/ATCC | 70.37 |
| EKVX | 81.87 |
| HOP-62 | 82.50 |
| HOP-92 | 84.08 |
| NCI-H226 | 68.52 |
| NCI-H23 | 77.68 |
| NCI-H322M | 87.06 |
| NCI-H460 | 65.24 |
| NCI-H522 | 70.51 |
| Colon Cancer | |
| COLO 205 | 72.86 |
| HCC-2998 | 51.39 |
| HCT-116 | 40.08 |
| HCT-15 | 65.78 |
| HT29 | 55.74 |
| KM12 | 66.35 |
| SW-620 | 69.16 |
| CNS Cancer | |
| SF-268 | 68.53 |
| SF-295 | 91.35 |
| SF-539 | 73.90 |
| SNB-19 | 72.88 |
| SNB-75 | 52.78 |
| U251 | 61.97 |
| Melanoma | |
| LOX IMVI | 65.77 |
| MALME-3M | 76.61 |
| M14 | 65.90 |
| MDA-MB-435 | 65.18 |
| SK-MEL-2 | 94.19 |
| SK-MEL-28 | 77.82 |
| SK-MEL-5 | 74.03 |
| UACC-257 | 72.85 |
| UACC-62 | 76.93 |
| Ovarian Cancer | |
| IGROV1 | 77.14 |
| OVCAR-3 | 66.26 |
| OVCAR-4 | 79.19 |
| OVCAR-5 | 78.63 |
| OVCAR-8 | 99.14 |
| NCI/ADR-RES | 74.35 |
| SK-OV-3 | 84.04 |
| Renal Cancer | |
| 786-0 | 64.31 |
| A498 | 106.50 |
| ACHN | 67.96 |
| CAKI-1 | 54.59 |
| RXF 393 | 54.09 |
| SN12C | 61.56 |
| TK-10 | 97.74 |
| UO-31 | 46.00 |
| Prostate Cancer | |
| PC-3 | 32.08 |
| DU-145 | 70.69 |
| Breast Cancer | |
| MCF7 | 52.62 |
| MDA-MB-231/ATCC | 61.49 |
| HS 578T | 63.02 |
| BT-549 | 100.66 |
| T-47D | 58.03 |
| MDA-MB-468 | 67.26 |
| Mean | 68.42 |
| Delta | 37.25 |
| Range | 75.33 |

**Mean Growth Percent - Growth Percent**

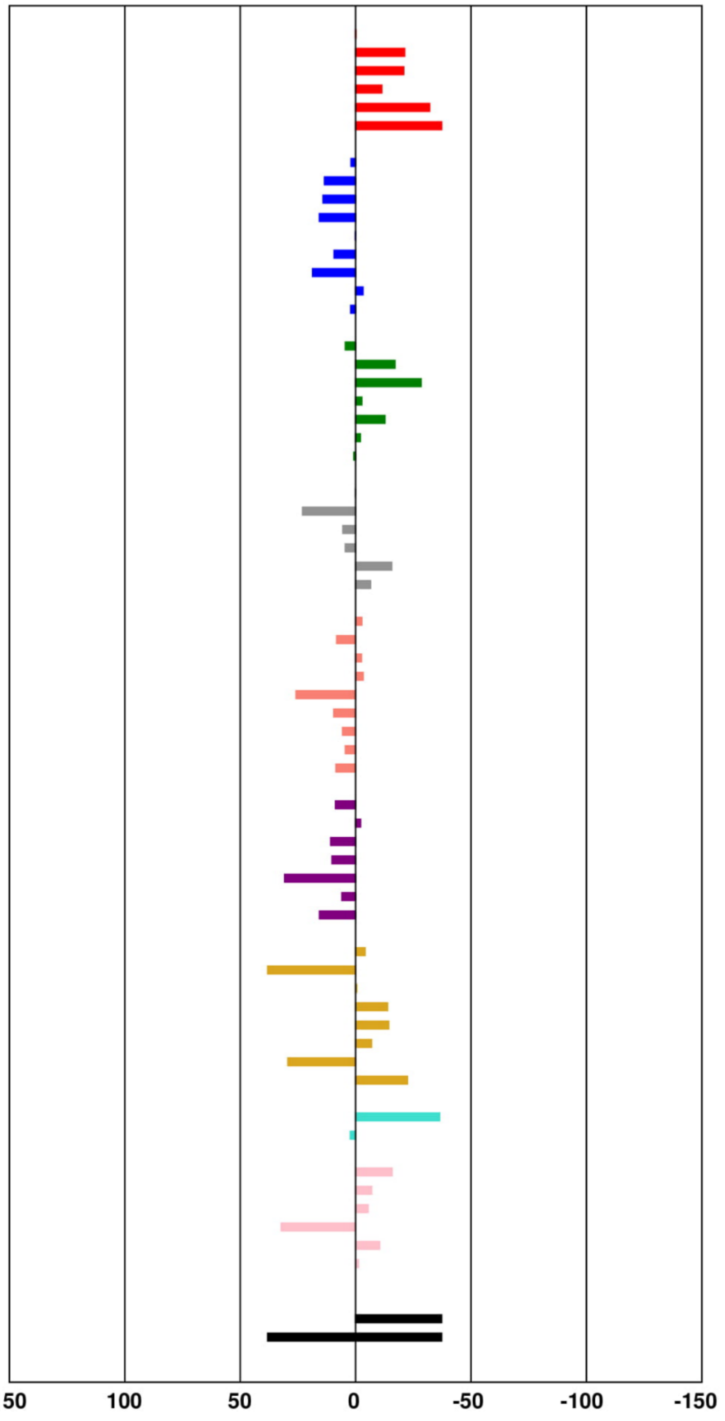


**Figure S16**. NCI-60 one dose data for compound **10**

| National Cancer Institute Developmental Therapeutics Program<br>Dose Response Curves | NSC: D - 811946 / 1 | SSPL: 1AJD | EXP. ID: 1902NS68 |
|---|---|---|---|
| | Report Date: March 16, 2019 | Test Date: February 25, 2019 | |

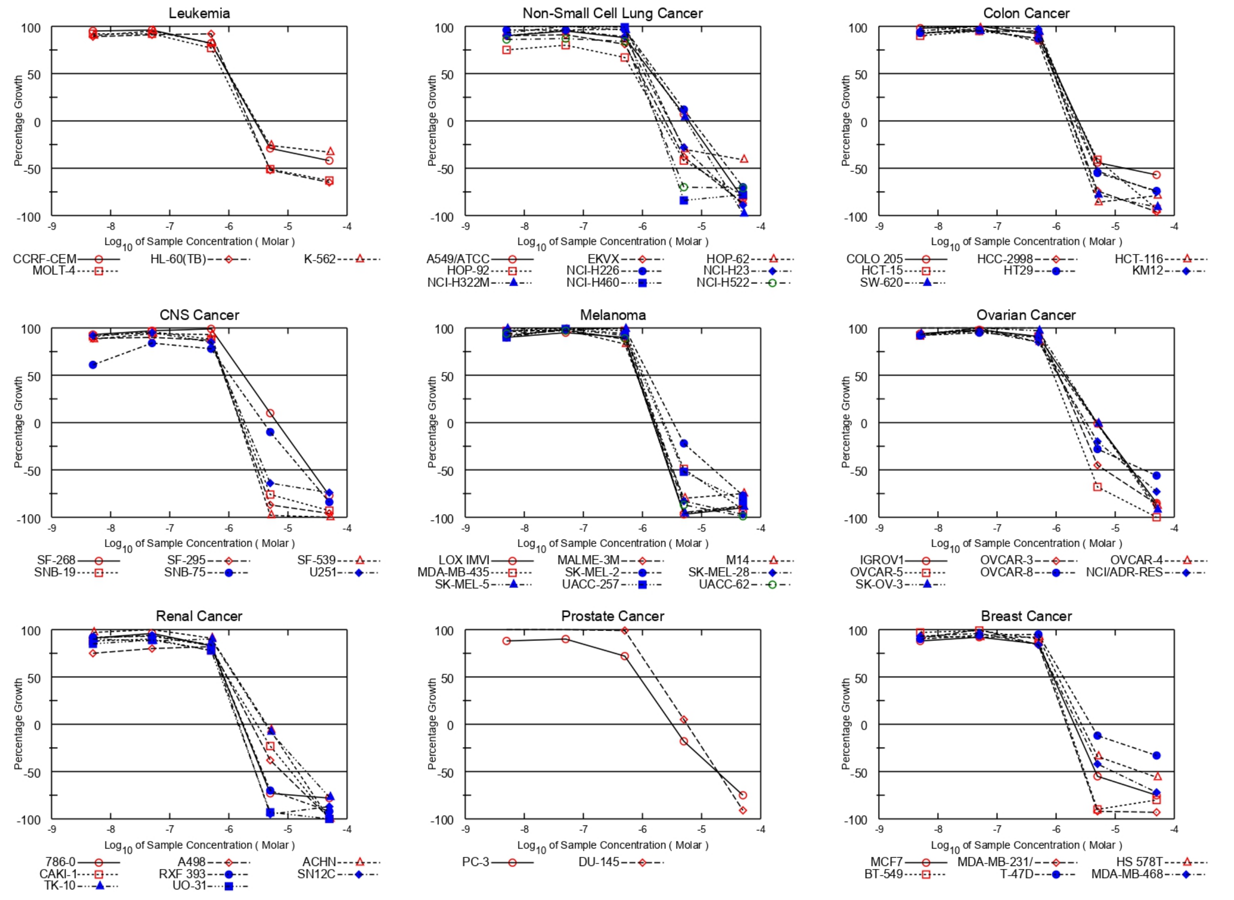

# National Cancer Institute Developmental Therapeutics Program
# In-Vitro Testing Results

| NSC : D - 811946 / 1 | Experiment ID : 1902NS68 | Test Type : 08 | Units : Molar |
|---|---|---|---|
| Report Date : March 16, 2019 | Test Date : February 25, 2019 | QNS : | MC : |
| COMI : PAV-114 | Stain Reagent : SRB Dual-Pass Related | SSPL : 1AJD | |

| | | | Log10 Concentration | | | | | | | | | | | | |
|---|---|---|---|---|---|---|---|---|---|---|---|---|---|---|---|
| | Time | | Mean Optical Densities | | | | | Percent Growth | | | | | | | |
| Panel/Cell Line | Zero | Ctrl | -8.3 | -7.3 | -6.3 | -5.3 | -4.3 | -8.3 | -7.3 | -6.3 | -5.3 | -4.3 | GI50 | TGI | LC50 |
| **Leukemia** | | | | | | | | | | | | | | | |
| CCRF-CEM | 0.479 | 2.636 | 2.519 | 2.561 | 2.257 | 0.340 | 0.278 | 95 | 96 | 82 | -29 | -42 | 9.76E-7 | 2.74E-6 | > 5.00E-5 |
| HL-60(TB) | 0.854 | 3.137 | 2.894 | 2.941 | 2.958 | 0.410 | 0.300 | 89 | 91 | 92 | -52 | -65 | 9.80E-7 | 2.18E-6 | 4.84E-6 |
| K-562 | 0.266 | 2.149 | 1.964 | 2.060 | 1.814 | 0.198 | 0.179 | 90 | 95 | 82 | -26 | -33 | 9.94E-7 | 2.90E-6 | > 5.00E-5 |
| MOLT-4 | 0.712 | 2.871 | 2.703 | 2.709 | 2.368 | 0.348 | 0.267 | 92 | 92 | 77 | -51 | -63 | 8.09E-7 | 1.99E-6 | 4.90E-6 |
| **Non-Small Cell Lung Cancer** | | | | | | | | | | | | | | | |
| A549/ATCC | 0.377 | 1.875 | 1.719 | 1.806 | 1.698 | 0.481 | 0.068 | 90 | 95 | 88 | 7 | -82 | 1.47E-6 | 5.98E-6 | 2.18E-5 |
| EKVX | 0.541 | 1.478 | 1.389 | 1.390 | 1.302 | 0.338 | 0.071 | 90 | 91 | 81 | -38 | -87 | 9.16E-7 | 2.42E-6 | 8.95E-6 |
| HOP-62 | 0.545 | 1.436 | 1.432 | 1.496 | 1.549 | 0.383 | 0.321 | 100 | 107 | 113 | -30 | -41 | 1.38E-6 | 3.09E-6 | > 5.00E-5 |
| HOP-92 | 1.331 | 1.918 | 1.772 | 1.800 | 1.723 | 0.774 | 0.273 | 75 | 80 | 67 | -42 | -79 | 7.13E-7 | 2.06E-6 | 8.22E-6 |
| NCI-H226 | 1.088 | 1.968 | 1.932 | 1.935 | 1.941 | 1.194 | 0.330 | 96 | 96 | 97 | 12 | -70 | 1.78E-6 | 7.01E-6 | 2.87E-5 |
| NCI-H23 | 0.618 | 1.929 | 1.798 | 1.881 | 1.788 | 0.447 | 0.069 | 90 | 96 | 89 | -28 | -89 | 1.08E-6 | 2.90E-6 | 1.16E-5 |
| NCI-H322M | 0.722 | 1.960 | 1.880 | 1.956 | 1.911 | 0.767 | 0.013 | 93 | 100 | 96 | 4 | -98 | 1.57E-6 | 5.43E-6 | 1.68E-5 |
| NCI-H460 | 0.281 | 2.379 | 2.396 | 2.508 | 2.366 | 0.046 | 0.061 | 101 | 106 | 99 | -84 | -78 | 9.31E-7 | 1.75E-6 | 3.27E-6 |
| NCI-H522 | 0.575 | 1.516 | 1.382 | 1.395 | 1.366 | 0.170 | 0.165 | 86 | 87 | 84 | -70 | -71 | 8.31E-7 | 1.75E-6 | 3.69E-6 |
| **Colon Cancer** | | | | | | | | | | | | | | | |
| COLO 205 | 0.565 | 2.235 | 2.197 | 2.325 | 2.098 | 0.317 | 0.243 | 98 | 105 | 92 | -44 | -57 | 1.02E-6 | 2.37E-6 | 1.45E-5 |
| HCC-2998 | 0.399 | 1.355 | 1.291 | 1.309 | 1.307 | 0.104 | 0.017 | 93 | 95 | 95 | -74 | -96 | 9.22E-7 | 1.82E-6 | 3.60E-6 |
| HCT-116 | 0.281 | 2.368 | 2.203 | 2.317 | 2.045 | 0.041 | 0.060 | 92 | 98 | 84 | -86 | -79 | 7.98E-7 | 1.57E-6 | 3.09E-6 |
| HCT-15 | 0.330 | 2.258 | 2.072 | 2.166 | 2.016 | 0.194 | 0.024 | 90 | 95 | 87 | -41 | -93 | 9.77E-7 | 2.39E-6 | 7.41E-6 |
| HT29 | 0.343 | 1.887 | 1.784 | 1.823 | 1.690 | 0.154 | 0.088 | 93 | 96 | 87 | -55 | -74 | 9.13E-7 | 2.05E-6 | 4.59E-6 |
| KM12 | 0.403 | 1.907 | 1.834 | 1.931 | 1.863 | 0.191 | 0.103 | 95 | 102 | 97 | -53 | -75 | 1.03E-6 | 2.22E-6 | 4.79E-6 |
| SW-620 | 0.296 | 1.795 | 1.793 | 1.726 | 1.707 | 0.065 | 0.027 | 100 | 95 | 94 | -78 | -91 | 9.02E-7 | 1.76E-6 | 3.44E-6 |
| **CNS Cancer** | | | | | | | | | | | | | | | |
| SF-268 | 0.554 | 1.914 | 1.817 | 1.873 | 1.901 | 0.687 | 0.129 | 93 | 97 | 99 | 10 | -77 | 1.77E-6 | 6.49E-6 | 2.46E-5 |
| SF-295 | 0.604 | 2.350 | 2.152 | 2.176 | 2.129 | 0.081 | 0.023 | 89 | 90 | 87 | -87 | -96 | 8.20E-7 | 1.59E-6 | 3.08E-6 |
| SF-539 | 0.892 | 2.637 | 2.433 | 2.541 | 2.523 | 0.016 | -0.007 | 88 | 94 | 93 | -98 | -100 | 8.43E-7 | 1.54E-6 | 2.80E-6 |
| SNB-19 | 0.500 | 2.090 | 1.946 | 2.021 | 1.894 | 0.121 | 0.035 | 91 | 96 | 88 | -76 | -93 | 8.49E-7 | 1.72E-6 | 3.47E-6 |
| SNB-75 | 1.185 | 2.038 | 1.708 | 1.900 | 1.849 | 1.063 | 0.187 | 61 | 84 | 78 | -10 | -84 | 1.03E-6 | 3.82E-6 | 1.72E-5 |
| U251 | 0.333 | 1.723 | 1.606 | 1.657 | 1.509 | 0.119 | 0.086 | 92 | 95 | 85 | -64 | -74 | 8.54E-7 | 1.85E-6 | 4.00E-6 |
| **Melanoma** | | | | | | | | | | | | | | | |
| LOX IMVI | 0.260 | 1.870 | 1.708 | 1.786 | 1.715 | 0.008 | 0.026 | 90 | 95 | 90 | -97 | -90 | 8.21E-7 | 1.52E-6 | 2.80E-6 |
| MALME-3M | 0.711 | 1.775 | 1.695 | 1.758 | 1.648 | 0.035 | 0.082 | 92 | 98 | 88 | -95 | -88 | 8.07E-7 | 1.51E-6 | 2.84E-6 |
| M14 | 0.415 | 1.670 | 1.615 | 1.648 | 1.452 | 0.085 | 0.103 | 96 | 98 | 83 | -80 | -75 | 7.94E-7 | 1.61E-6 | 3.28E-6 |
| MDA-MB-435 | 0.560 | 2.344 | 2.295 | 2.369 | 2.289 | 0.285 | 0.047 | 97 | 101 | 97 | -49 | -92 | 1.05E-6 | 2.30E-6 | 5.22E-6 |
| SK-MEL-2 | 1.442 | 2.923 | 2.874 | 2.893 | 2.929 | 1.131 | 0.329 | 97 | 98 | 100 | -22 | -77 | 1.29E-6 | 3.33E-6 | 1.62E-5 |
| SK-MEL-28 | 0.632 | 1.856 | 1.752 | 1.849 | 1.786 | 0.109 | 0.016 | 91 | 99 | 94 | -83 | -97 | 8.89E-7 | 1.70E-6 | 3.27E-6 |
| SK-MEL-5 | 0.998 | 3.271 | 3.241 | 3.275 | 3.241 | 0.036 | 0.108 | 99 | 100 | 99 | -96 | -89 | 8.88E-7 | 1.60E-6 | 2.89E-6 |
| UACC-257 | 0.984 | 2.353 | 2.214 | 2.338 | 2.249 | 0.477 | 0.169 | 90 | 99 | 92 | -52 | -83 | 9.85E-7 | 2.19E-6 | 4.88E-6 |
| UACC-62 | 0.847 | 2.792 | 2.678 | 2.731 | 2.581 | 0.113 | 0.006 | 94 | 97 | 89 | -87 | -99 | 8.35E-7 | 1.61E-6 | 3.09E-6 |
| **Ovarian Cancer** | | | | | | | | | | | | | | | |
| IGROV1 | 0.413 | 1.809 | 1.728 | 1.778 | 1.687 | 0.408 | 0.062 | 94 | 98 | 91 | -1 | -85 | 1.40E-6 | 4.85E-6 | 1.91E-5 |
| OVCAR-3 | 0.274 | 0.830 | 0.838 | 0.866 | 0.828 | 0.150 | 0.038 | 101 | 106 | 100 | -45 | -86 | 1.10E-6 | 2.44E-6 | 6.52E-6 |
| OVCAR-4 | 0.583 | 1.202 | 1.157 | 1.250 | 1.108 | 0.572 | 0.069 | 93 | 108 | 85 | -2 | -88 | 1.26E-6 | 4.74E-6 | 1.80E-5 |
| OVCAR-5 | 0.585 | 1.647 | 1.557 | 1.611 | 1.552 | 0.189 | -0.007 | 92 | 97 | 91 | -68 | -100 | 9.07E-7 | 1.87E-6 | 3.87E-6 |
| OVCAR-8 | 0.412 | 1.911 | 1.787 | 1.840 | 1.768 | 0.296 | 0.181 | 92 | 95 | 90 | -28 | -56 | 1.10E-6 | 2.89E-6 | 3.01E-5 |
| NCI/ADR-RES | 0.644 | 2.341 | 2.228 | 2.289 | 2.091 | 0.516 | 0.172 | 93 | 97 | 85 | -20 | -73 | 1.08E-6 | 3.24E-6 | 1.83E-5 |
| SK-OV-3 | 0.956 | 2.102 | 2.026 | 2.125 | 2.073 | 0.942 | 0.077 | 93 | 102 | 97 | -1 | -92 | 1.51E-6 | 4.83E-6 | 1.72E-5 |
| **Renal Cancer** | | | | | | | | | | | | | | | |
| 786-0 | 0.557 | 2.467 | 2.302 | 2.381 | 2.144 | 0.150 | 0.123 | 91 | 96 | 83 | -73 | -78 | 8.14E-7 | 1.70E-6 | 3.56E-6 |
| A498 | 1.673 | 2.468 | 2.270 | 2.311 | 2.326 | 1.043 | 0.010 | 75 | 80 | 82 | -38 | -99 | 9.27E-7 | 2.42E-6 | 7.92E-6 |
| ACHN | 0.436 | 1.870 | 1.823 | 1.903 | 1.740 | 0.412 | -0.027 | 97 | 102 | 91 | -6 | -100 | 1.33E-6 | 4.38E-6 | 1.48E-5 |
| CAKI-1 | 0.499 | 2.233 | 2.034 | 2.056 | 1.851 | 0.384 | 0.002 | 88 | 90 | 78 | -23 | -100 | 9.46E-7 | 2.96E-6 | 1.12E-5 |
| RXF 393 | 0.772 | 1.582 | 1.513 | 1.529 | 1.446 | 0.229 | 0.065 | 92 | 93 | 83 | -70 | -92 | 8.22E-7 | 1.74E-6 | 3.69E-6 |
| SN12C | 0.477 | 1.876 | 1.746 | 1.795 | 1.654 | 0.025 | 0.064 | 91 | 94 | 84 | -95 | -87 | 7.76E-7 | 1.48E-6 | 2.81E-6 |
| TK-10 | 0.963 | 2.433 | 2.282 | 2.254 | 2.283 | 0.886 | 0.222 | 90 | 88 | 90 | -8 | -77 | 1.27E-6 | 4.14E-6 | 2.03E-5 |
| UO-31 | 0.484 | 1.685 | 1.504 | 1.551 | 1.418 | 0.033 | -0.028 | 85 | 89 | 78 | -93 | -100 | 7.26E-7 | 1.42E-6 | 2.79E-6 |
| **Prostate Cancer** | | | | | | | | | | | | | | | |
| PC-3 | 0.655 | 1.917 | 1.766 | 1.785 | 1.560 | 0.540 | 0.164 | 88 | 90 | 72 | -18 | -75 | 8.75E-7 | 3.17E-6 | 1.83E-5 |
| DU-145 | 0.348 | 1.407 | 1.405 | 1.496 | 1.402 | 0.400 | 0.031 | 100 | 108 | 99 | 5 | -91 | 1.67E-6 | 5.62E-6 | 1.86E-5 |
| **Breast Cancer** | | | | | | | | | | | | | | | |
| MCF7 | 0.309 | 1.762 | 1.589 | 1.651 | 1.546 | 0.140 | 0.077 | 88 | 92 | 85 | -55 | -75 | 8.92E-7 | 2.03E-6 | 4.63E-6 |
| MDA-MB-231/ATCC | 0.556 | 1.485 | 1.403 | 1.502 | 1.335 | 0.044 | 0.038 | 91 | 102 | 84 | -92 | -93 | 7.79E-7 | 1.50E-6 | 2.88E-6 |
| HS 578T | 0.942 | 1.807 | 1.744 | 1.735 | 1.735 | 0.621 | 0.414 | 93 | 92 | 92 | -34 | -56 | 1.07E-6 | 2.68E-6 | 2.65E-5 |
| BT-549 | 1.116 | 2.293 | 2.256 | 2.286 | 2.174 | 0.112 | 0.222 | 97 | 99 | 90 | -90 | -80 | 8.33E-7 | 1.58E-6 | 3.00E-6 |
| T-47D | 0.853 | 2.013 | 1.900 | 1.946 | 1.960 | 0.753 | 0.574 | 90 | 94 | 95 | -12 | -33 | 1.33E-6 | 3.89E-6 | > 5.00E-5 |
| MDA-MB-468 | 0.913 | 1.700 | 1.645 | 1.673 | 1.575 | 0.530 | 0.257 | 93 | 96 | 84 | -42 | -72 | 9.31E-7 | 2.32E-6 | 9.27E-6 |

National Cancer Institute Developmental Therapeutics Program
Mean Graphs

NSC : D - 811946/1 | Units :Molar | SSPL :1AJD | EXP. ID :1902NS68

Report Date :March 16, 2019 | Test Date :February 25, 2019

| Panel/Cell Line | $Log_{10}$GI50 | $Log_{10}$TGI | $Log_{10}$LC50 |
|---|---|---|---|
| **Leukemia** | | | |
| CCRF-CEM | -6.01 | -5.56 | > -4.30 |
| HL-60(TB) | -6.01 | -5.66 | -5.32 |
| K-562 | -6.00 | -5.54 | > -4.30 |
| MOLT-4 | -6.09 | -5.70 | -5.31 |
| **Non-Small Cell Lung Cancer** | | | |
| A549/ATCC | -5.83 | -5.22 | -4.66 |
| EKVX | -6.04 | -5.62 | -5.05 |
| HOP-62 | -5.86 | -5.51 | > -4.30 |
| HOP-92 | -6.15 | -5.69 | -5.09 |
| NCI-H226 | -5.75 | -5.15 | -4.54 |
| NCI-H23 | -5.97 | -5.54 | -4.94 |
| NCI-H322M | -5.80 | -5.27 | -4.77 |
| NCI-H460 | -6.03 | -5.76 | -5.48 |
| NCI-H522 | -6.08 | -5.76 | -5.43 |
| **Colon Cancer** | | | |
| COLO 205 | -5.99 | -5.62 | -4.84 |
| HCC-2998 | -6.04 | -5.74 | -5.44 |
| HCT-116 | -6.10 | -5.80 | -5.51 |
| HCT-15 | -6.01 | -5.62 | -5.13 |
| HT29 | -6.04 | -5.69 | -5.34 |
| KM12 | -5.99 | -5.65 | -5.32 |
| SW-620 | -6.04 | -5.75 | -5.46 |
| **CNS Cancer** | | | |
| SF-268 | -5.75 | -5.19 | -4.61 |
| SF-295 | -6.09 | -5.80 | -5.51 |
| SF-539 | -6.07 | -5.81 | -5.55 |
| SNB-19 | -6.07 | -5.77 | -5.46 |
| SNB-75 | -5.99 | -5.42 | -4.76 |
| U251 | -6.07 | -5.73 | -5.40 |
| **Melanoma** | | | |
| LOX IMVI | -6.09 | -5.82 | -5.55 |
| MALME-3M | -6.09 | -5.82 | -5.55 |
| M14 | -6.10 | -5.79 | -5.48 |
| MDA-MB-435 | -5.98 | -5.64 | -5.28 |
| SK-MEL-2 | -5.89 | -5.48 | -4.79 |
| SK-MEL-28 | -6.05 | -5.77 | -5.49 |
| SK-MEL-5 | -6.05 | -5.80 | -5.54 |
| UACC-257 | -6.01 | -5.66 | -5.31 |
| UACC-62 | -6.08 | -5.79 | -5.51 |
| **Ovarian Cancer** | | | |
| IGROV1 | -5.85 | -5.31 | -4.72 |
| OVCAR-3 | -5.96 | -5.61 | -5.19 |
| OVCAR-4 | -5.90 | -5.32 | -4.74 |
| OVCAR-5 | -6.04 | -5.73 | -5.41 |
| OVCAR-8 | -5.96 | -5.54 | -4.52 |
| NCI/ADR-RES | -5.97 | -5.49 | -4.74 |
| SK-OV-3 | -5.82 | -5.32 | -4.76 |
| **Renal Cancer** | | | |
| 786-0 | -6.09 | -5.77 | -5.45 |
| A498 | -6.03 | -5.62 | -5.10 |
| ACHN | -5.88 | -5.36 | -4.83 |
| CAKI-1 | -6.02 | -5.53 | -4.95 |
| RXF 393 | -6.08 | -5.76 | -5.43 |
| SN12C | -6.11 | -5.83 | -5.55 |
| TK-10 | -5.89 | -5.38 | -4.69 |
| UO-31 | -6.14 | -5.85 | -5.55 |
| **Prostate Cancer** | | | |
| PC-3 | -6.06 | -5.50 | -4.74 |
| DU-145 | -5.78 | -5.25 | -4.73 |
| **Breast Cancer** | | | |
| MCF7 | -6.05 | -5.69 | -5.33 |
| MDA-MB-231/ATCC | -6.11 | -5.82 | -5.54 |
| HS 578T | -5.97 | -5.57 | -4.58 |
| BT-549 | -6.08 | -5.80 | -5.52 |
| T-47D | -5.88 | -5.41 | > -4.30 |
| MDA-MB-468 | -6.03 | -5.63 | -5.03 |
| MID | -6.0 | -5.61 | -5.1 |
| Delta | 0.15 | 0.24 | 0.45 |
| Range | 0.4 | 0.7 | 1.25 |

GI50

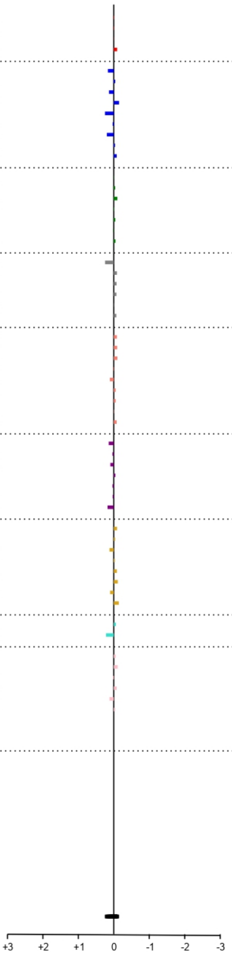


TGI

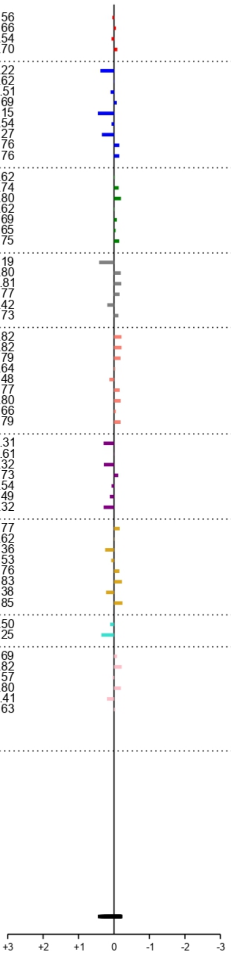


LC50

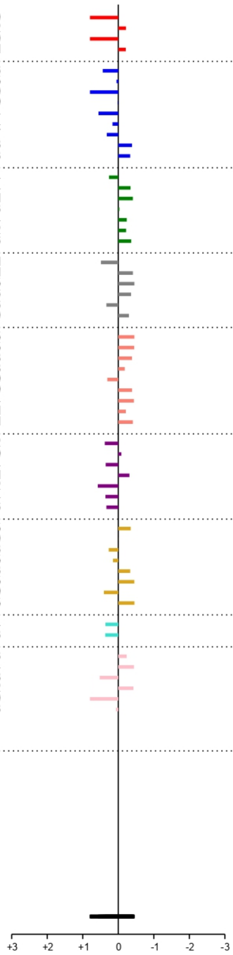

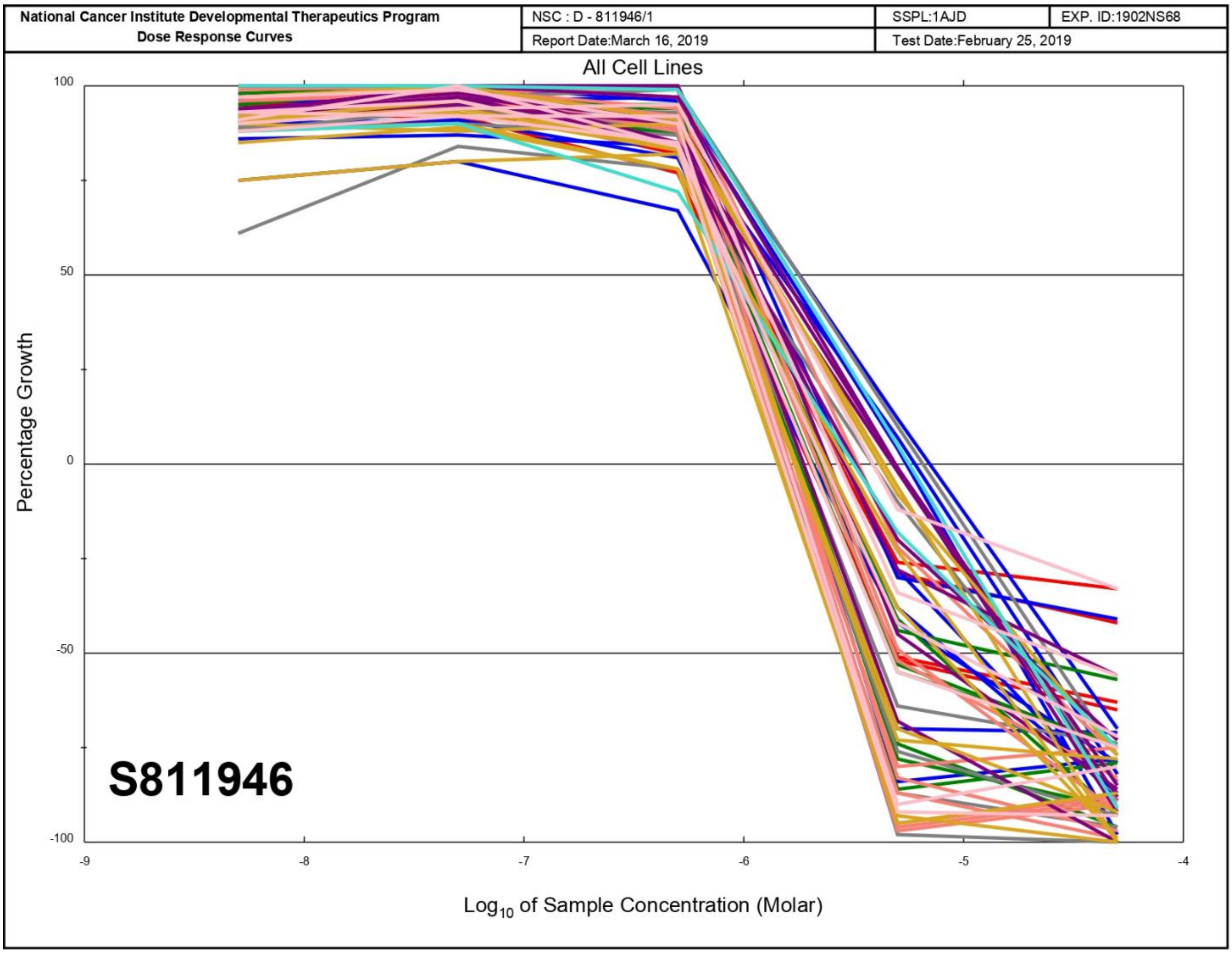


**Figure S17.** The five-dose assay data for compound **4**

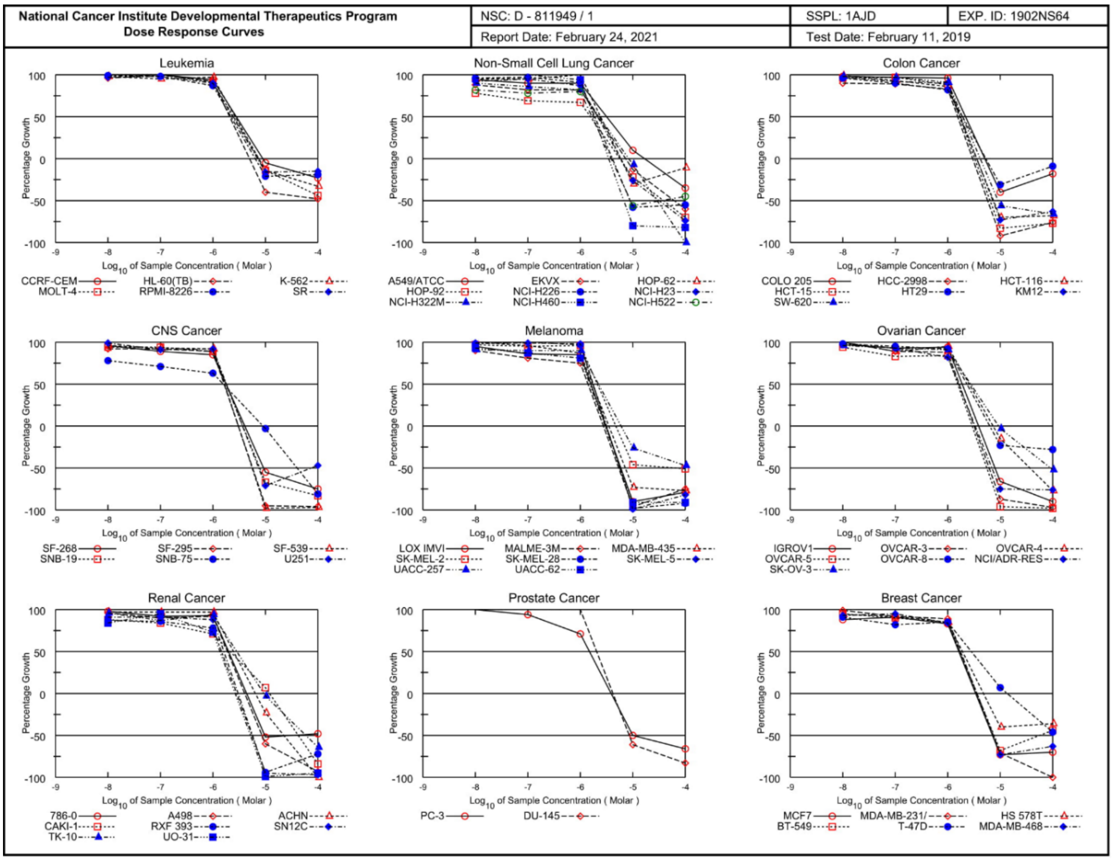
National Cancer Institute Developmental Therapeutics Program
Dose Response Curves
NSC: D - 811949 / 1
SSPL: 1AJD
EXP. ID: 1902NS64
Report Date: February 24, 2021
Test Date: February 11, 2019
Leukemia
Non-Small Cell Lung Cancer
Colon Cancer
CNS Cancer
Melanoma
Ovarian Cancer
Renal Cancer
Prostate Cancer
Breast Cancer
Percentage Growth
Log10 of Sample Concentration ( Molar )
CCRF-CEM
HL-60(TB)
K-562
MOLT-4
RPMI-8226
SR
A549/ATCC
EKVX
HOP-62
HOP-92
NCI-H226
NCI-H23
NCI-H322M
NCI-H460
NCI-H522
COLO 205
HCC-2998
HCT-116
HCT-15
HT29
KM12
SW-620
SF-268
SF-295
SF-539
SNB-19
SNB-75
U251
LOX IMVI
MALME-3M
MDA-MB-435
SK-MEL-2
SK-MEL-28
SK-MEL-5
UACC-257
UACC-62
IGROV1
OVCAR-3
OVCAR-4
OVCAR-5
OVCAR-8
NCI/ADR-RES
SK-OV-3
786-0
A498
ACHN
CAKI-1
RXF 393
SN12C
TK-10
UO-31
PC-3
DU-145
MCF7
MDA-MB-231/
HS 578T
BT-549
T-47D
MDA-MB-468

# National Cancer Institute Developmental Therapeutics Program
# In-Vitro Testing Results

| NSC : D - 811949 / 1 | Experiment ID : 1902NS64 | Test Type : 08 | Units : Molar |
|---|---|---|---|
| Report Date : February 24, 2021 | Test Date : February 11, 2019 | QNS : | MC : |
| COMI : PAV-114 | Stain Reagent : SRB Dual-Pass Related | SSPL : 1AJD | |

| | Time | | Log10 Concentration | | | | | | | | | | | | |
|---|---|---|---|---|---|---|---|---|---|---|---|---|---|---|---|
| | | | Mean Optical Densities | | | | | Percent Growth | | | | | | | |
| Panel/Cell Line | Zero | Ctrl | -8.0 | -7.0 | -6.0 | -5.0 | -4.0 | -8.0 | -7.0 | -6.0 | -5.0 | -4.0 | GI50 | TGI | LC50 |
| **Leukemia** | | | | | | | | | | | | | | | |
| CCRF-CEM | 0.622 | 2.800 | 2.814 | 2.826 | 2.662 | 0.593 | 0.476 | 101 | 101 | 94 | -5 | -23 | 2.78E-6 | 8.97E-6 | > 1.00E-4 |
| HL-60(TB) | 1.030 | 3.033 | 2.963 | 3.105 | 2.865 | 0.623 | 0.538 | 96 | 104 | 92 | -40 | -48 | 2.08E-6 | 5.00E-6 | > 1.00E-4 |
| K-562 | 0.277 | 1.732 | 1.710 | 1.662 | 1.683 | 0.238 | 0.185 | 98 | 95 | 97 | -14 | -33 | 2.63E-6 | 7.44E-6 | > 1.00E-4 |
| MOLT-4 | 0.799 | 2.830 | 3.012 | 2.967 | 2.643 | 0.689 | 0.449 | 109 | 107 | 91 | -14 | -44 | 2.45E-6 | 7.38E-6 | > 1.00E-4 |
| RPMI-8226 | 0.747 | 2.030 | 2.021 | 2.000 | 1.858 | 0.588 | 0.606 | 99 | 98 | 87 | -21 | -19 | 2.18E-6 | 6.35E-6 | > 1.00E-4 |
| SR | 0.381 | 1.717 | 1.677 | 1.687 | 1.578 | 0.321 | 0.325 | 97 | 98 | 90 | -16 | -15 | 2.38E-6 | 7.09E-6 | > 1.00E-4 |
| **Non-Small Cell Lung Cancer** | | | | | | | | | | | | | | | |
| A549/ATCC | 0.489 | 2.069 | 1.979 | 1.907 | 1.906 | 0.649 | 0.318 | 94 | 90 | 90 | 10 | -35 | 3.15E-6 | 1.68E-5 | > 1.00E-4 |
| EKVX | 0.764 | 2.386 | 2.184 | 2.101 | 2.090 | 0.666 | 0.307 | 88 | 82 | 82 | -13 | -60 | 2.17E-6 | 7.32E-6 | 6.17E-5 |
| HOP-62 | 0.392 | 1.140 | 1.105 | 1.104 | 1.143 | 0.273 | 0.348 | 95 | 95 | 100 | -30 | -11 | 2.43E-6 | 5.86E-6 | > 1.00E-4 |
| HOP-92 | 1.558 | 2.226 | 2.078 | 2.016 | 2.009 | 1.223 | 0.469 | 78 | 69 | 67 | -22 | -70 | 1.57E-6 | 5.73E-6 | 3.88E-5 |
| NCI-H226 | 1.598 | 3.110 | 3.047 | 3.061 | 2.979 | 0.664 | 0.712 | 96 | 97 | 91 | -58 | -55 | 1.89E-6 | 4.07E-6 | 8.78E-6 |
| NCI-H23 | 0.658 | 2.000 | 1.893 | 1.928 | 1.825 | 0.489 | 0.171 | 92 | 95 | 87 | -26 | -74 | 2.13E-6 | 5.91E-6 | 3.18E-5 |
| NCI-H322M | 0.616 | 1.511 | 1.425 | 1.382 | 1.349 | 0.571 | -0.023 | 90 | 86 | 82 | -7 | -100 | 2.28E-6 | 8.27E-6 | 2.88E-5 |
| NCI-H460 | 0.242 | 1.934 | 1.982 | 1.928 | 1.828 | 0.048 | 0.044 | 103 | 100 | 94 | -80 | -82 | 1.78E-6 | 3.45E-6 | 6.69E-6 |
| NCI-H522 | 0.957 | 2.450 | 2.175 | 2.125 | 2.147 | 0.420 | 0.528 | 82 | 78 | 80 | -56 | -45 | 1.66E-6 | 3.86E-6 | . |
| **Colon Cancer** | | | | | | | | | | | | | | | |
| COLO 205 | 0.455 | 1.711 | 1.683 | 1.673 | 1.665 | 0.275 | 0.372 | 98 | 97 | 96 | -40 | -18 | 2.19E-6 | 5.12E-6 | > 1.00E-4 |
| HCC-2998 | 0.647 | 2.114 | 1.962 | 1.953 | 1.870 | 0.055 | 0.155 | 90 | 89 | 83 | -92 | -76 | 1.55E-6 | 3.00E-6 | 5.79E-6 |
| HCT-116 | 0.117 | 1.290 | 1.273 | 1.207 | 1.122 | 0.035 | 0.038 | 99 | 93 | 86 | -70 | -68 | 1.69E-6 | 3.55E-6 | 7.43E-6 |
| HCT-15 | 0.376 | 2.767 | 2.677 | 2.693 | 2.468 | 0.064 | 0.087 | 96 | 97 | 88 | -83 | -77 | 1.66E-6 | 3.26E-6 | 6.41E-6 |
| HT29 | 0.234 | 1.556 | 1.507 | 1.426 | 1.321 | 0.163 | 0.213 | 96 | 90 | 82 | -31 | -9 | 1.93E-6 | 5.36E-6 | > 1.00E-4 |
| KM12 | 0.477 | 2.173 | 2.126 | 2.045 | 1.999 | 0.129 | 0.175 | 97 | 92 | 90 | -73 | -63 | 1.75E-6 | 3.56E-6 | 7.23E-6 |
| SW-620 | 0.298 | 1.690 | 1.658 | 1.653 | 1.564 | 0.130 | 0.101 | 98 | 97 | 91 | -56 | -66 | 1.90E-6 | 4.14E-6 | 9.05E-6 |
| **CNS Cancer** | | | | | | | | | | | | | | | |
| SF-268 | 0.687 | 2.020 | 1.967 | 1.877 | 1.815 | 0.307 | 0.171 | 96 | 89 | 85 | -55 | -75 | 1.77E-6 | 4.02E-6 | 9.15E-6 |
| SF-295 | 0.929 | 3.113 | 2.946 | 2.942 | 2.878 | 0.046 | 0.034 | 92 | 92 | 89 | -95 | -96 | 1.63E-6 | 3.05E-6 | 5.70E-6 |
| SF-539 | 0.669 | 1.958 | 1.907 | 1.871 | 1.855 | 0.013 | 0.017 | 96 | 93 | 92 | -98 | -97 | 1.66E-6 | 3.05E-6 | 5.58E-6 |
| SNB-19 | 0.555 | 1.816 | 1.739 | 1.738 | 1.681 | 0.185 | 0.092 | 94 | 94 | 89 | -67 | -83 | 1.79E-6 | 3.74E-6 | 7.82E-6 |
| SNB-75 | 1.008 | 1.667 | 1.522 | 1.478 | 1.424 | 0.975 | 0.192 | 78 | 71 | 63 | -3 | -81 | 1.57E-6 | 8.91E-6 | 3.99E-5 |
| U251 | 0.453 | 1.936 | 1.918 | 1.803 | 1.823 | 0.131 | 0.240 | 99 | 91 | 92 | -71 | -47 | 1.82E-6 | 3.67E-6 | . |
| **Melanoma** | | | | | | | | | | | | | | | |
| LOX IMVI | 0.316 | 1.861 | 1.786 | 1.644 | 1.628 | 0.032 | 0.066 | 95 | 86 | 85 | -90 | -79 | 1.58E-6 | 3.06E-6 | 5.91E-6 |
| MALME-3M | 0.731 | 1.590 | 1.508 | 1.425 | 1.377 | 0.030 | 0.180 | 90 | 81 | 75 | -96 | -75 | 1.40E-6 | 2.75E-6 | 5.39E-6 |
| MDA-MB-435 | 0.486 | 1.909 | 1.929 | 1.846 | 1.718 | 0.132 | 0.113 | 101 | 96 | 87 | -73 | -77 | 1.70E-6 | 3.49E-6 | 7.18E-6 |
| SK-MEL-2 | 1.650 | 3.143 | 3.103 | 3.065 | 3.090 | 0.894 | 0.810 | 97 | 95 | 96 | -46 | -51 | 2.12E-6 | 4.76E-6 | 6.55E-5 |
| SK-MEL-28 | 0.726 | 1.799 | 1.779 | 1.827 | 1.768 | 0.017 | 0.060 | 98 | 103 | 97 | -98 | -92 | 1.74E-6 | 3.15E-6 | 5.69E-6 |
| SK-MEL-5 | 1.339 | 3.342 | 3.326 | 3.322 | 3.296 | 0.011 | 0.245 | 99 | 99 | 98 | -99 | -82 | 1.75E-6 | 3.14E-6 | 5.63E-6 |
| UACC-257 | 1.139 | 2.248 | 2.145 | 2.137 | 2.139 | 0.841 | 0.601 | 91 | 90 | 90 | -26 | -47 | 2.21E-6 | 5.95E-6 | > 1.00E-4 |
| UACC-62 | 0.932 | 2.583 | 2.461 | 2.373 | 2.262 | 0.089 | 0.080 | 93 | 87 | 81 | -91 | -91 | 1.51E-6 | 2.96E-6 | 5.80E-6 |
| **Ovarian Cancer** | | | | | | | | | | | | | | | |
| IGROV1 | 0.463 | 1.727 | 1.688 | 1.638 | 1.657 | 0.157 | 0.046 | 97 | 93 | 94 | -66 | -90 | 1.89E-6 | 3.87E-6 | 7.94E-6 |
| OVCAR-3 | 0.421 | 1.138 | 1.160 | 1.062 | 1.052 | 0.057 | 0.012 | 103 | 89 | 88 | -87 | -97 | 1.65E-6 | 3.19E-6 | 6.17E-6 |
| OVCAR-4 | 0.554 | 1.149 | 1.171 | 1.085 | 1.120 | 0.473 | 0.126 | 104 | 89 | 95 | -15 | -77 | 2.57E-6 | 7.36E-6 | 3.66E-5 |
| OVCAR-5 | 0.547 | 1.463 | 1.413 | 1.312 | 1.315 | 0.025 | 0.012 | 94 | 83 | 84 | -96 | -98 | 1.54E-6 | 2.93E-6 | 5.57E-6 |
| OVCAR-8 | 0.470 | 1.975 | 1.941 | 1.905 | 1.856 | 0.361 | 0.338 | 98 | 95 | 92 | -23 | -28 | 2.32E-6 | 6.29E-6 | > 1.00E-4 |
| NCI/ADR-RES | 0.562 | 2.168 | 2.141 | 2.049 | 1.871 | 0.140 | 0.136 | 98 | 93 | 82 | -75 | -76 | 1.59E-6 | 3.31E-6 | 6.91E-6 |
| SK-OV-3 | 0.834 | 1.944 | 1.898 | 1.865 | 1.839 | 0.806 | 0.399 | 96 | 93 | 91 | -3 | -52 | 2.70E-6 | 9.20E-6 | 9.01E-5 |
| **Renal Cancer** | | | | | | | | | | | | | | | |
| 786-0 | 0.599 | 2.825 | 2.792 | 2.649 | 2.677 | 0.286 | 0.312 | 98 | 92 | 93 | -52 | -48 | 1.98E-6 | 4.37E-6 | . |
| A498 | 1.687 | 2.877 | 2.830 | 2.757 | 2.783 | 0.682 | 0.119 | 96 | 90 | 92 | -60 | -93 | 1.90E-6 | 4.05E-6 | 8.65E-6 |
| ACHN | 0.282 | 1.246 | 1.213 | 1.221 | 1.213 | 0.218 | -0.012 | 97 | 97 | 97 | -23 | -100 | 2.46E-6 | 6.45E-6 | 2.26E-5 |
| CAKI-1 | 0.658 | 2.557 | 2.330 | 2.250 | 2.007 | 0.784 | 0.103 | 88 | 84 | 71 | 7 | -84 | 2.12E-6 | 1.18E-5 | 4.19E-5 |
| RXF 393 | 1.105 | 1.582 | 1.521 | 1.516 | 1.475 | 0.064 | 0.312 | 87 | 86 | 78 | -94 | -72 | 1.45E-6 | 2.83E-6 | 5.53E-6 |
| SN12C | 0.507 | 1.712 | 1.601 | 1.609 | 1.563 | 0.024 | 0.015 | 91 | 91 | 88 | -95 | -97 | 1.61E-6 | 3.01E-6 | 5.66E-6 |
| TK-10 | 0.916 | 2.333 | 2.278 | 2.148 | 2.253 | 0.892 | 0.331 | 96 | 87 | 94 | -3 | -64 | 2.87E-6 | 9.40E-6 | 5.93E-5 |
| UO-31 | 0.496 | 1.442 | 1.290 | 1.391 | 1.184 | 0.006 | 0.026 | 84 | 95 | 73 | -99 | -95 | 1.36E-6 | 2.65E-6 | 5.19E-6 |
| **Prostate Cancer** | | | | | | | | | | | | | | | |
| PC-3 | 0.567 | 2.121 | 2.119 | 2.023 | 1.673 | 0.281 | 0.195 | 100 | 94 | 71 | -50 | -66 | 1.49E-6 | 3.85E-6 | 9.92E-6 |
| DU-145 | 0.314 | 1.148 | 1.200 | 1.151 | 1.164 | 0.122 | 0.054 | 106 | 100 | 102 | -61 | -83 | 2.08E-6 | 4.21E-6 | 8.53E-6 |
| **Breast Cancer** | | | | | | | | | | | | | | | |
| MCF7 | 0.481 | 2.565 | 2.322 | 2.380 | 2.220 | 0.131 | 0.142 | 88 | 91 | 83 | -73 | -70 | 1.64E-6 | 3.42E-6 | 7.14E-6 |
| MDA-MB-231/ATCC | 0.636 | 1.419 | 1.412 | 1.360 | 1.330 | 0.185 | -0.023 | 99 | 92 | 89 | -71 | -100 | 1.75E-6 | 3.59E-6 | 7.39E-6 |
| HS 578T | 0.816 | 1.791 | 1.736 | 1.712 | 1.647 | 0.487 | 0.519 | 94 | 92 | 85 | -40 | -36 | 1.91E-6 | 4.77E-6 | > 1.00E-4 |
| BT-549 | 1.028 | 2.554 | 2.476 | 2.408 | 2.323 | 0.329 | 0.571 | 95 | 90 | 85 | -68 | -44 | 1.69E-6 | 3.59E-6 | . |
| T-47D | 0.830 | 1.875 | 1.782 | 1.691 | 1.717 | 0.899 | 0.452 | 91 | 82 | 85 | 7 | -46 | 2.79E-6 | 1.34E-5 | > 1.00E-4 |
| MDA-MB-468 | 0.895 | 1.528 | 1.483 | 1.496 | 1.430 | 0.238 | 0.330 | 93 | 95 | 84 | -73 | -63 | 1.65E-6 | 3.43E-6 | 7.10E-6 |

| National Cancer Institute Developmental Therapeutics Program Mean Graphs | NSC : D - 811949/1 | Units :Molar | SSPL :1AJD | EXP. ID :1902NS64 |
|---|---|---|---|---|
| | Report Date :February 24, 2021 | | Test Date :February 11, 2019 | |

| Panel/Cell Line | $Log_{10}$GI50 | $Log_{10}$TGI | $Log_{10}$LC50 |
|---|---|---|---|
| **Leukemia** | | | |
| CCRF-CEM | -5.56 | -5.05 | > -4.00 |
| HL-60(TB) | -5.68 | -5.30 | > -4.00 |
| K-562 | -5.58 | -5.13 | > -4.00 |
| MOLT-4 | -5.61 | -5.13 | > -4.00 |
| RPMI-8226 | -5.66 | -5.20 | > -4.00 |
| SR | -5.62 | -5.15 | > -4.00 |
| **Non-Small Cell Lung Cancer** | | | |
| A549/ATCC | -5.50 | -4.78 | > -4.00 |
| EKVX | -5.66 | -5.14 | -4.21 |
| HOP-62 | -5.62 | -5.23 | > -4.00 |
| HOP-92 | -5.80 | -5.24 | -4.41 |
| NCI-H226 | -5.72 | -5.39 | -5.06 |
| NCI-H23 | -5.67 | -5.23 | -4.50 |
| NCI-H322M | -5.64 | -5.08 | -4.54 |
| NCI-H460 | -5.75 | -5.46 | -5.17 |
| NCI-H522 | -5.78 | -5.41 | |
| **Colon Cancer** | | | |
| COLO 205 | -5.66 | -5.29 | > -4.00 |
| HCC-2998 | -5.81 | -5.52 | -5.24 |
| HCT-116 | -5.77 | -5.45 | -5.13 |
| HCT-15 | -5.78 | -5.49 | -5.19 |
| HT29 | -5.71 | -5.27 | > -4.00 |
| KM12 | -5.76 | -5.45 | -5.14 |
| SW-620 | -5.72 | -5.38 | -5.04 |
| **CNS Cancer** | | | |
| SF-268 | -5.75 | -5.40 | -5.04 |
| SF-295 | -5.79 | -5.52 | -5.24 |
| SF-539 | -5.78 | -5.52 | -5.25 |
| SNB-19 | -5.75 | -5.43 | -5.11 |
| SNB-75 | -5.80 | -5.05 | -4.40 |
| U251 | -5.74 | -5.44 | |
| **Melanoma** | | | |
| LOX IMVI | -5.80 | -5.51 | -5.23 |
| MALME-3M | -5.85 | -5.56 | -5.27 |
| MDA-MB-435 | -5.77 | -5.46 | -5.14 |
| SK-MEL-2 | -5.67 | -5.32 | -4.18 |
| SK-MEL-28 | -5.76 | -5.50 | -5.24 |
| SK-MEL-5 | -5.76 | -5.50 | -5.25 |
| UACC-257 | -5.65 | -5.23 | > -4.00 |
| UACC-62 | -5.82 | -5.53 | -5.24 |
| **Ovarian Cancer** | | | |
| IGROV1 | -5.72 | -5.41 | -5.10 |
| OVCAR-3 | -5.78 | -5.50 | -5.21 |
| OVCAR-4 | -5.59 | -5.13 | -4.44 |
| OVCAR-5 | -5.81 | -5.53 | -5.25 |
| OVCAR-8 | -5.64 | -5.20 | > -4.00 |
| NCI/ADR-RES | -5.80 | -5.48 | -5.16 |
| SK-OV-3 | -5.57 | -5.04 | -4.05 |
| **Renal Cancer** | | | |
| 786-0 | -5.70 | -5.36 | |
| A498 | -5.72 | -5.39 | -5.06 |
| ACHN | -5.61 | -5.19 | -4.65 |
| CAKI-1 | -5.67 | -4.93 | -4.38 |
| RXF 393 | -5.84 | -5.55 | -5.26 |
| SN12C | -5.79 | -5.52 | -5.25 |
| TK-10 | -5.54 | -5.03 | -4.23 |
| UO-31 | -5.87 | -5.58 | -5.28 |
| **Prostate Cancer** | | | |
| PC-3 | -5.83 | -5.41 | -5.00 |
| DU-145 | -5.68 | -5.38 | -5.07 |
| **Breast Cancer** | | | |
| MCF7 | -5.79 | -5.47 | -5.15 |
| MDA-MB-231/ATCC | -5.76 | -5.44 | -5.13 |
| HS 578T | -5.72 | -5.32 | > -4.00 |
| BT-549 | -5.77 | -5.44 | |
| T-47D | -5.55 | -4.87 | > -4.00 |
| MDA-MB-468 | -5.78 | -5.47 | -5.15 |
| MID | -5.72 | -5.33 | -4.71 |
| Delta | 0.15 | 0.25 | 0.57 |
| Range | 0.37 | 0.8 | 1.28 |

Axis (each graph): +3 +2 +1 0 -1 -2 -3

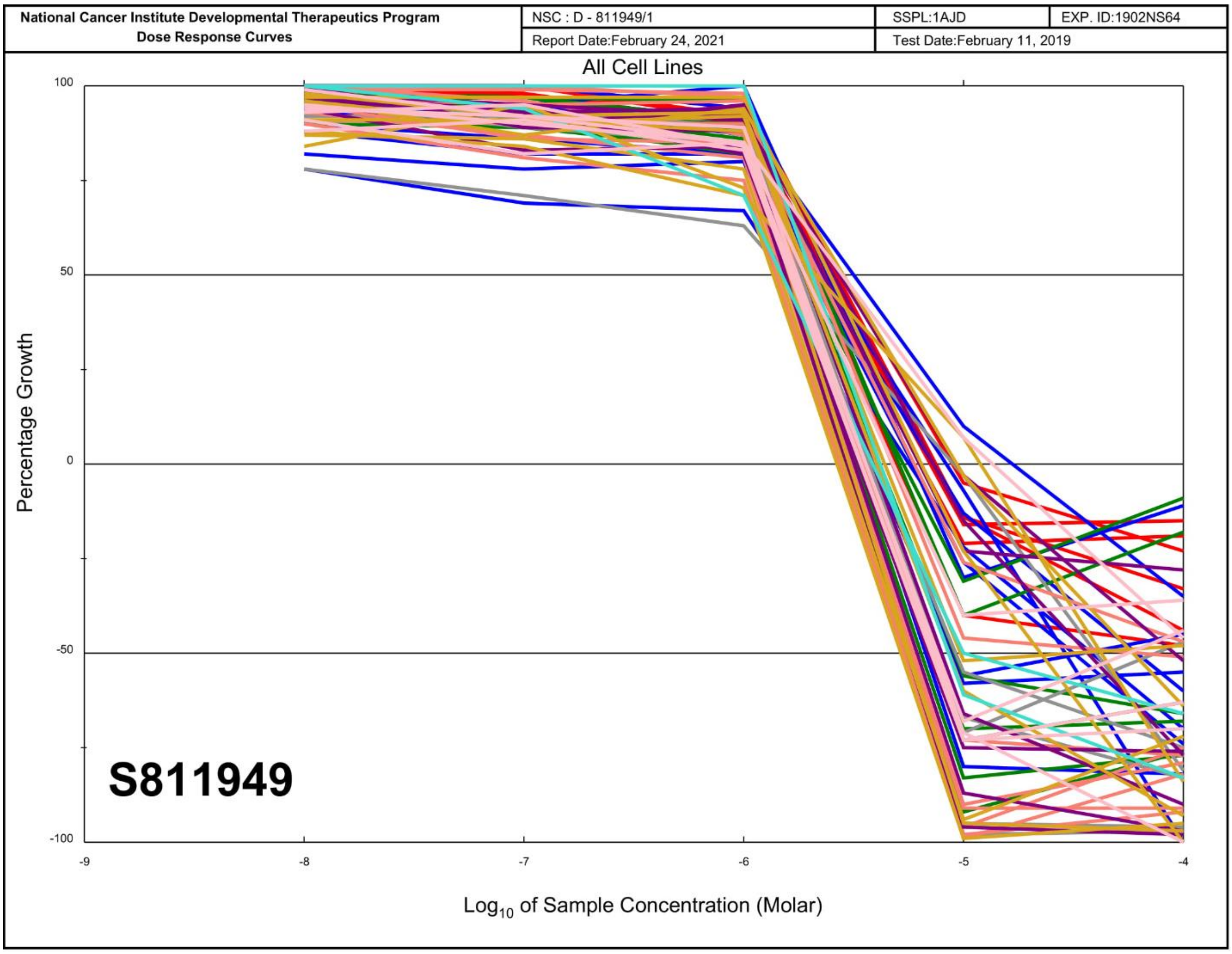


**Figure S18.** The five-dose assay data for compound **6**

| National Cancer Institute Developmental Therapeutics Program<br>Dose Response Curves | NSC: D - 811949 / 1 | SSPL: 1AJD | EXP. ID: 1902NS64 |
|---|---|---|---|
| | Report Date: March 16, 2019 | Test Date: February 11, 2019 | |

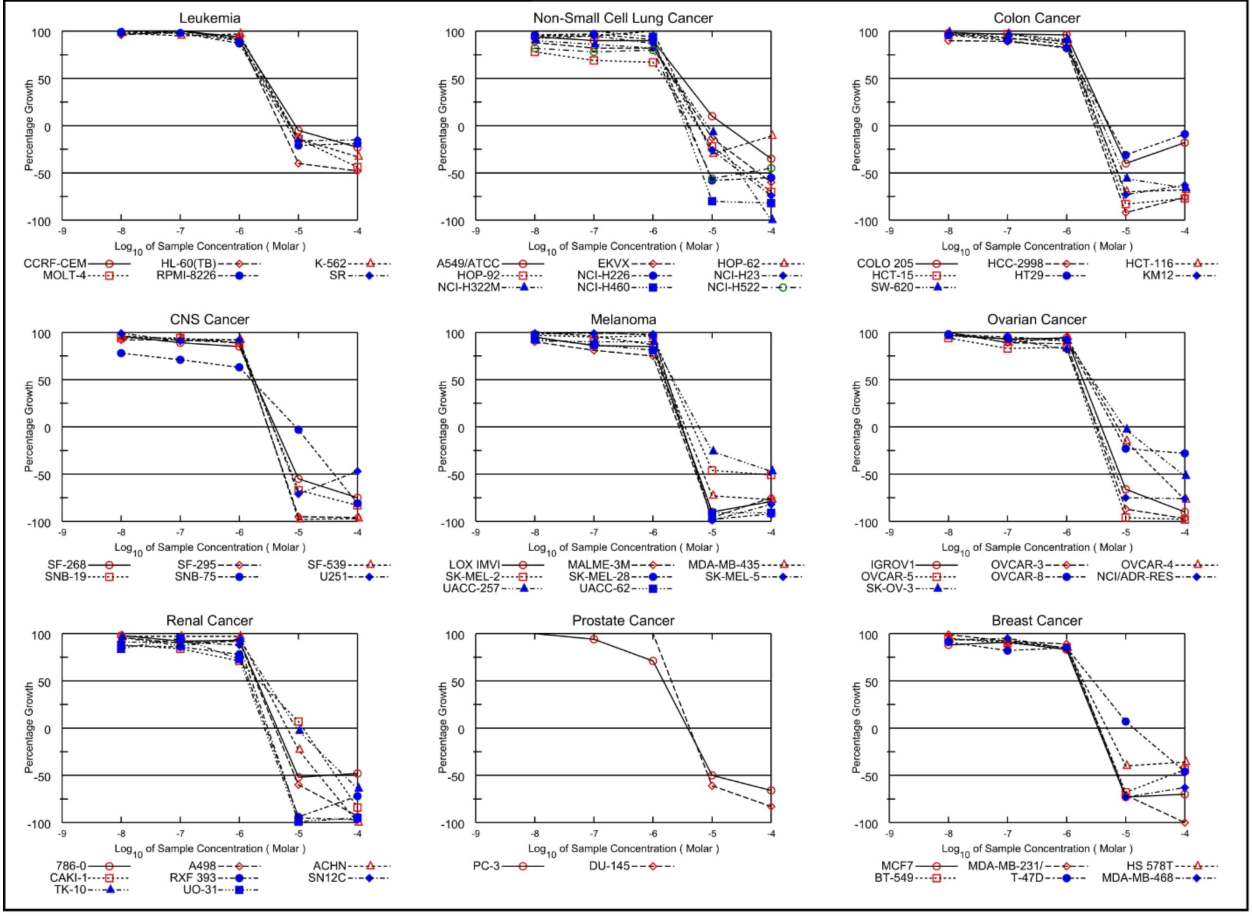

# National Cancer Institute Developmental Therapeutics Program
## In-Vitro Testing Results

| NSC : D - 811949 / 1 | Experiment ID : 1902NS64 | Test Type : 08 | Units : Molar |
|---|---|---|---|
| Report Date : March 16, 2019 | Test Date : February 11, 2019 | QNS : | MC : |
| COMI : PAV-114 | Stain Reagent : SRB Dual-Pass Related | SSPL : 1AJD | |

| | | | Log10 Concentration | | | | | | | | | | | | |
|---|---|---|---|---|---|---|---|---|---|---|---|---|---|---|---|
| | Time | | Mean Optical Densities | | | | | Percent Growth | | | | | | | |
| Panel/Cell Line | Zero | Ctrl | -8.0 | -7.0 | -6.0 | -5.0 | -4.0 | -8.0 | -7.0 | -6.0 | -5.0 | -4.0 | GI50 | TGI | LC50 |
| **Leukemia** | | | | | | | | | | | | | | | |
| CCRF-CEM | 0.622 | 2.800 | 2.814 | 2.826 | 2.662 | 0.593 | 0.476 | 101 | 101 | 94 | -5 | -23 | 2.78E-6 | 8.97E-6 | > 1.00E-4 |
| HL-60(TB) | 1.030 | 3.033 | 2.963 | 3.105 | 2.865 | 0.623 | 0.538 | 96 | 104 | 92 | -40 | -48 | 2.08E-6 | 5.00E-6 | > 1.00E-4 |
| K-562 | 0.277 | 1.732 | 1.710 | 1.662 | 1.683 | 0.238 | 0.185 | 98 | 95 | 97 | -14 | -33 | 2.63E-6 | 7.44E-6 | > 1.00E-4 |
| MOLT-4 | 0.799 | 2.830 | 3.012 | 2.967 | 2.643 | 0.689 | 0.449 | 109 | 107 | 91 | -14 | -44 | 2.45E-6 | 7.38E-6 | > 1.00E-4 |
| RPMI-8226 | 0.747 | 2.030 | 2.021 | 2.000 | 1.858 | 0.588 | 0.606 | 99 | 98 | 87 | -21 | -19 | 2.18E-6 | 6.35E-6 | > 1.00E-4 |
| SR | 0.381 | 1.717 | 1.677 | 1.687 | 1.578 | 0.321 | 0.325 | 97 | 98 | 90 | -16 | -15 | 2.38E-6 | 7.09E-6 | > 1.00E-4 |
| **Non-Small Cell Lung Cancer** | | | | | | | | | | | | | | | |
| A549/ATCC | 0.489 | 2.069 | 1.979 | 1.907 | 1.906 | 0.649 | 0.318 | 94 | 90 | 90 | 10 | -35 | 3.15E-6 | 1.68E-5 | > 1.00E-4 |
| EKVX | 0.764 | 2.386 | 2.184 | 2.101 | 2.090 | 0.666 | 0.307 | 88 | 82 | 82 | -13 | -60 | 2.17E-6 | 7.32E-6 | 6.17E-5 |
| HOP-62 | 0.392 | 1.140 | 1.105 | 1.104 | 1.143 | 0.273 | 0.348 | 95 | 95 | 100 | -30 | -11 | 2.43E-6 | 5.86E-6 | > 1.00E-4 |
| HOP-92 | 1.558 | 2.226 | 2.078 | 2.016 | 2.009 | 1.223 | 0.469 | 78 | 69 | 67 | -22 | -70 | 1.57E-6 | 5.73E-6 | 3.88E-5 |
| NCI-H226 | 1.598 | 3.110 | 3.047 | 3.061 | 2.979 | 0.664 | 0.712 | 96 | 97 | 91 | -58 | -55 | 1.89E-6 | 4.07E-6 | 8.78E-6 |
| NCI-H23 | 0.658 | 2.000 | 1.893 | 1.928 | 1.825 | 0.489 | 0.171 | 92 | 95 | 87 | -26 | -74 | 2.13E-6 | 5.91E-6 | 3.18E-5 |
| NCI-H322M | 0.616 | 1.511 | 1.425 | 1.382 | 1.349 | 0.571 | -0.023 | 90 | 86 | 82 | -7 | -100 | 2.28E-6 | 8.27E-6 | 2.88E-5 |
| NCI-H460 | 0.242 | 1.934 | 1.982 | 1.928 | 1.828 | 0.048 | 0.044 | 103 | 100 | 94 | -80 | -82 | 1.78E-6 | 3.45E-6 | 6.69E-6 |
| NCI-H522 | 0.957 | 2.450 | 2.175 | 2.125 | 2.147 | 0.420 | 0.528 | 82 | 78 | 80 | -56 | -45 | 1.66E-6 | 3.86E-6 | . |
| **Colon Cancer** | | | | | | | | | | | | | | | |
| COLO 205 | 0.455 | 1.711 | 1.683 | 1.673 | 1.665 | 0.275 | 0.372 | 98 | 97 | 96 | -40 | -18 | 2.19E-6 | 5.12E-6 | > 1.00E-4 |
| HCC-2998 | 0.647 | 2.114 | 1.962 | 1.953 | 1.870 | 0.055 | 0.155 | 90 | 89 | 83 | -92 | -76 | 1.55E-6 | 3.00E-6 | 5.79E-6 |
| HCT-116 | 0.117 | 1.290 | 1.273 | 1.207 | 1.122 | 0.035 | 0.038 | 99 | 93 | 86 | -70 | -68 | 1.69E-6 | 3.55E-6 | 7.43E-6 |
| HCT-15 | 0.376 | 2.767 | 2.677 | 2.693 | 2.468 | 0.064 | 0.087 | 96 | 97 | 88 | -83 | -77 | 1.66E-6 | 3.26E-6 | 6.41E-6 |
| HT29 | 0.234 | 1.556 | 1.507 | 1.426 | 1.321 | 0.163 | 0.213 | 96 | 90 | 82 | -31 | -9 | 1.93E-6 | 5.36E-6 | > 1.00E-4 |
| KM12 | 0.477 | 2.173 | 2.126 | 2.045 | 1.999 | 0.129 | 0.175 | 97 | 92 | 90 | -73 | -63 | 1.75E-6 | 3.56E-6 | 7.23E-6 |
| SW-620 | 0.298 | 1.690 | 1.658 | 1.653 | 1.564 | 0.130 | 0.101 | 98 | 97 | 91 | -56 | -66 | 1.90E-6 | 4.14E-6 | 9.05E-6 |
| **CNS Cancer** | | | | | | | | | | | | | | | |
| SF-268 | 0.687 | 2.020 | 1.967 | 1.877 | 1.815 | 0.307 | 0.171 | 96 | 89 | 85 | -55 | -75 | 1.77E-6 | 4.02E-6 | 9.15E-6 |
| SF-295 | 0.929 | 3.113 | 2.946 | 2.942 | 2.878 | 0.046 | 0.034 | 92 | 92 | 89 | -95 | -96 | 1.63E-6 | 3.05E-6 | 5.70E-6 |
| SF-539 | 0.669 | 1.958 | 1.907 | 1.871 | 1.855 | 0.013 | 0.017 | 96 | 93 | 92 | -98 | -97 | 1.66E-6 | 3.05E-6 | 5.58E-6 |
| SNB-19 | 0.555 | 1.816 | 1.739 | 1.738 | 1.681 | 0.185 | 0.092 | 94 | 94 | 89 | -67 | -83 | 1.79E-6 | 3.74E-6 | 7.82E-6 |
| SNB-75 | 1.008 | 1.667 | 1.522 | 1.478 | 1.424 | 0.975 | 0.192 | 78 | 71 | 63 | -3 | -81 | 1.57E-6 | 8.91E-6 | 3.99E-5 |
| U251 | 0.453 | 1.936 | 1.918 | 1.803 | 1.823 | 0.131 | 0.240 | 99 | 91 | 92 | -71 | -47 | 1.82E-6 | 3.67E-6 | . |
| **Melanoma** | | | | | | | | | | | | | | | |
| LOX IMVI | 0.316 | 1.861 | 1.786 | 1.644 | 1.628 | 0.032 | 0.066 | 95 | 86 | 85 | -90 | -79 | 1.58E-6 | 3.06E-6 | 5.91E-6 |
| MALME-3M | 0.731 | 1.590 | 1.508 | 1.425 | 1.377 | 0.030 | 0.180 | 90 | 81 | 75 | -96 | -75 | 1.40E-6 | 2.75E-6 | 5.39E-6 |
| MDA-MB-435 | 0.486 | 1.909 | 1.929 | 1.846 | 1.718 | 0.132 | 0.113 | 101 | 96 | 87 | -73 | -77 | 1.70E-6 | 3.49E-6 | 7.18E-6 |
| SK-MEL-2 | 1.650 | 3.143 | 3.103 | 3.065 | 3.090 | 0.894 | 0.810 | 97 | 95 | 96 | -46 | -51 | 2.12E-6 | 4.76E-6 | 6.55E-5 |
| SK-MEL-28 | 0.726 | 1.799 | 1.779 | 1.827 | 1.768 | 0.017 | 0.060 | 98 | 103 | 97 | -98 | -92 | 1.74E-6 | 3.15E-6 | 5.69E-6 |
| SK-MEL-5 | 1.339 | 3.342 | 3.326 | 3.322 | 3.296 | 0.011 | 0.245 | 99 | 99 | 98 | -99 | -82 | 1.75E-6 | 3.14E-6 | 5.63E-6 |
| UACC-257 | 1.139 | 2.248 | 2.145 | 2.137 | 2.139 | 0.841 | 0.601 | 91 | 90 | 90 | -26 | -47 | 2.21E-6 | 5.95E-6 | > 1.00E-4 |
| UACC-62 | 0.932 | 2.583 | 2.461 | 2.373 | 2.262 | 0.089 | 0.080 | 93 | 87 | 81 | -91 | -91 | 1.51E-6 | 2.96E-6 | 5.80E-6 |
| **Ovarian Cancer** | | | | | | | | | | | | | | | |
| IGROV1 | 0.463 | 1.727 | 1.688 | 1.638 | 1.657 | 0.157 | 0.046 | 97 | 93 | 94 | -66 | -90 | 1.89E-6 | 3.87E-6 | 7.94E-6 |
| OVCAR-3 | 0.421 | 1.138 | 1.160 | 1.062 | 1.052 | 0.057 | 0.012 | 103 | 89 | 88 | -87 | -97 | 1.65E-6 | 3.19E-6 | 6.17E-6 |
| OVCAR-4 | 0.554 | 1.149 | 1.171 | 1.085 | 1.120 | 0.473 | 0.126 | 104 | 89 | 95 | -15 | -77 | 2.57E-6 | 7.36E-6 | 3.66E-5 |
| OVCAR-5 | 0.547 | 1.463 | 1.413 | 1.312 | 1.315 | 0.025 | 0.012 | 94 | 83 | 84 | -96 | -98 | 1.54E-6 | 2.93E-6 | 5.57E-6 |
| OVCAR-8 | 0.470 | 1.975 | 1.941 | 1.905 | 1.856 | 0.361 | 0.338 | 98 | 95 | 92 | -23 | -28 | 2.32E-6 | 6.29E-6 | > 1.00E-4 |
| NCI/ADR-RES | 0.562 | 2.168 | 2.141 | 2.049 | 1.871 | 0.140 | 0.136 | 98 | 93 | 82 | -75 | -76 | 1.59E-6 | 3.31E-6 | 6.91E-6 |
| SK-OV-3 | 0.834 | 1.944 | 1.898 | 1.865 | 1.839 | 0.806 | 0.399 | 96 | 93 | 91 | -3 | -52 | 2.70E-6 | 9.20E-6 | 9.01E-5 |
| **Renal Cancer** | | | | | | | | | | | | | | | |
| 786-0 | 0.599 | 2.825 | 2.792 | 2.649 | 2.677 | 0.286 | 0.312 | 98 | 92 | 93 | -52 | -48 | 1.98E-6 | 4.37E-6 | . |
| A498 | 1.687 | 2.877 | 2.830 | 2.757 | 2.783 | 0.682 | 0.119 | 96 | 90 | 92 | -60 | -93 | 1.90E-6 | 4.05E-6 | 8.65E-6 |
| ACHN | 0.282 | 1.246 | 1.213 | 1.221 | 1.213 | 0.218 | -0.012 | 97 | 97 | 97 | -23 | -100 | 2.46E-6 | 6.45E-6 | 2.26E-5 |
| CAKI-1 | 0.658 | 2.557 | 2.330 | 2.250 | 2.007 | 0.784 | 0.103 | 88 | 84 | 71 | 7 | -84 | 2.12E-6 | 1.18E-5 | 4.19E-5 |
| RXF 393 | 1.105 | 1.582 | 1.521 | 1.516 | 1.475 | 0.064 | 0.312 | 87 | 86 | 78 | -94 | -72 | 1.45E-6 | 2.83E-6 | 5.53E-6 |
| SN12C | 0.507 | 1.712 | 1.601 | 1.609 | 1.563 | 0.024 | 0.015 | 91 | 91 | 88 | -95 | -97 | 1.61E-6 | 3.01E-6 | 5.66E-6 |
| TK-10 | 0.916 | 2.333 | 2.278 | 2.148 | 2.253 | 0.892 | 0.331 | 96 | 87 | 94 | -3 | -64 | 2.87E-6 | 9.40E-6 | 5.93E-5 |
| UO-31 | 0.496 | 1.442 | 1.290 | 1.391 | 1.184 | 0.006 | 0.026 | 84 | 95 | 73 | -99 | -95 | 1.36E-6 | 2.65E-6 | 5.19E-6 |
| **Prostate Cancer** | | | | | | | | | | | | | | | |
| PC-3 | 0.567 | 2.121 | 2.119 | 2.023 | 1.673 | 0.281 | 0.195 | 100 | 94 | 71 | -50 | -66 | 1.49E-6 | 3.85E-6 | 9.92E-6 |
| DU-145 | 0.314 | 1.148 | 1.200 | 1.151 | 1.164 | 0.122 | 0.054 | 106 | 100 | 102 | -61 | -83 | 2.08E-6 | 4.21E-6 | 8.53E-6 |
| **Breast Cancer** | | | | | | | | | | | | | | | |
| MCF7 | 0.481 | 2.565 | 2.322 | 2.380 | 2.220 | 0.131 | 0.142 | 88 | 91 | 83 | -73 | -70 | 1.64E-6 | 3.42E-6 | 7.14E-6 |
| MDA-MB-231/ATCC | 0.636 | 1.419 | 1.412 | 1.360 | 1.330 | 0.185 | -0.023 | 99 | 92 | 89 | -71 | -100 | 1.75E-6 | 3.59E-6 | 7.39E-6 |
| HS 578T | 0.816 | 1.791 | 1.736 | 1.712 | 1.647 | 0.487 | 0.519 | 94 | 92 | 85 | -40 | -36 | 1.91E-6 | 4.77E-6 | > 1.00E-4 |
| BT-549 | 1.028 | 2.554 | 2.476 | 2.408 | 2.323 | 0.329 | 0.571 | 95 | 90 | 85 | -68 | -44 | 1.69E-6 | 3.59E-6 | . |
| T-47D | 0.830 | 1.875 | 1.782 | 1.691 | 1.717 | 0.899 | 0.452 | 91 | 82 | 85 | 7 | -46 | 2.79E-6 | 1.34E-5 | > 1.00E-4 |
| MDA-MB-468 | 0.895 | 1.528 | 1.483 | 1.496 | 1.430 | 0.238 | 0.330 | 93 | 95 | 84 | -73 | -63 | 1.65E-6 | 3.43E-6 | 7.10E-6 |

| National Cancer Institute Developmental Therapeutics Program<br>Mean Graphs | NSC : D - 811949/1 | Units :Molar | SSPL :1AJD | EXP. ID :1902NS64 |
|---|---|---|---|---|
| | Report Date :March 16, 2019 | | Test Date :February 11, 2019 | |

| Panel/Cell Line | $Log_{10}$GI50 | $Log_{10}$TGI | $Log_{10}$LC50 |
|---|---|---|---|
| **Leukemia** | | | |
| CCRF-CEM | -5.56 | -5.05 | > -4.00 |
| HL-60(TB) | -5.68 | -5.30 | > -4.00 |
| K-562 | -5.58 | -5.13 | > -4.00 |
| MOLT-4 | -5.61 | -5.13 | > -4.00 |
| RPMI-8226 | -5.66 | -5.20 | > -4.00 |
| SR | -5.62 | -5.15 | > -4.00 |
| **Non-Small Cell Lung Cancer** | | | |
| A549/ATCC | -5.50 | -4.78 | > -4.00 |
| EKVX | -5.66 | -5.14 | -4.21 |
| HOP-62 | -5.62 | -5.23 | > -4.00 |
| HOP-92 | -5.80 | -5.24 | -4.41 |
| NCI-H226 | -5.72 | -5.39 | -5.06 |
| NCI-H23 | -5.67 | -5.23 | -4.50 |
| NCI-H322M | -5.64 | -5.08 | -4.54 |
| NCI-H460 | -5.75 | -5.46 | -5.17 |
| NCI-H522 | -5.78 | -5.41 | |
| **Colon Cancer** | | | |
| COLO 205 | -5.66 | -5.29 | > -4.00 |
| HCC-2998 | -5.81 | -5.52 | -5.24 |
| HCT-116 | -5.77 | -5.45 | -5.13 |
| HCT-15 | -5.78 | -5.49 | -5.19 |
| HT29 | -5.71 | -5.27 | > -4.00 |
| KM12 | -5.76 | -5.45 | -5.14 |
| SW-620 | -5.72 | -5.38 | -5.04 |
| **CNS Cancer** | | | |
| SF-268 | -5.75 | -5.40 | -5.04 |
| SF-295 | -5.79 | -5.52 | -5.24 |
| SF-539 | -5.78 | -5.52 | -5.25 |
| SNB-19 | -5.75 | -5.43 | -5.11 |
| SNB-75 | -5.80 | -5.05 | -4.40 |
| U251 | -5.74 | -5.44 | |
| **Melanoma** | | | |
| LOX IMVI | -5.80 | -5.51 | -5.23 |
| MALME-3M | -5.85 | -5.56 | -5.27 |
| MDA-MB-435 | -5.77 | -5.46 | -5.14 |
| SK-MEL-2 | -5.67 | -5.32 | -4.18 |
| SK-MEL-28 | -5.76 | -5.50 | -5.24 |
| SK-MEL-5 | -5.76 | -5.50 | -5.25 |
| UACC-257 | -5.65 | -5.23 | > -4.00 |
| UACC-62 | -5.82 | -5.53 | -5.24 |
| **Ovarian Cancer** | | | |
| IGROV1 | -5.72 | -5.41 | -5.10 |
| OVCAR-3 | -5.78 | -5.50 | -5.21 |
| OVCAR-4 | -5.59 | -5.13 | -4.44 |
| OVCAR-5 | -5.81 | -5.53 | -5.25 |
| OVCAR-8 | -5.64 | -5.20 | > -4.00 |
| NCI/ADR-RES | -5.80 | -5.48 | -5.16 |
| SK-OV-3 | -5.57 | -5.04 | -4.05 |
| **Renal Cancer** | | | |
| 786-0 | -5.70 | -5.36 | |
| A498 | -5.72 | -5.39 | -5.06 |
| ACHN | -5.61 | -5.19 | -4.65 |
| CAKI-1 | -5.67 | -4.93 | -4.38 |
| RXF 393 | -5.84 | -5.55 | -5.26 |
| SN12C | -5.79 | -5.52 | -5.25 |
| TK-10 | -5.54 | -5.03 | -4.23 |
| UO-31 | -5.87 | -5.58 | -5.28 |
| **Prostate Cancer** | | | |
| PC-3 | -5.83 | -5.41 | -5.00 |
| DU-145 | -5.68 | -5.38 | -5.07 |
| **Breast Cancer** | | | |
| MCF7 | -5.79 | -5.47 | -5.15 |
| MDA-MB-231/ATCC | -5.76 | -5.44 | -5.13 |
| HS 578T | -5.72 | -5.32 | > -4.00 |
| BT-549 | -5.77 | -5.44 | |
| T-47D | -5.55 | -4.87 | > -4.00 |
| MDA-MB-468 | -5.78 | -5.47 | -5.15 |
| MID | -5.72 | -5.33 | -4.71 |
| Delta | 0.15 | 0.25 | 0.57 |
| Range | 0.37 | 0.8 | 1.28 |

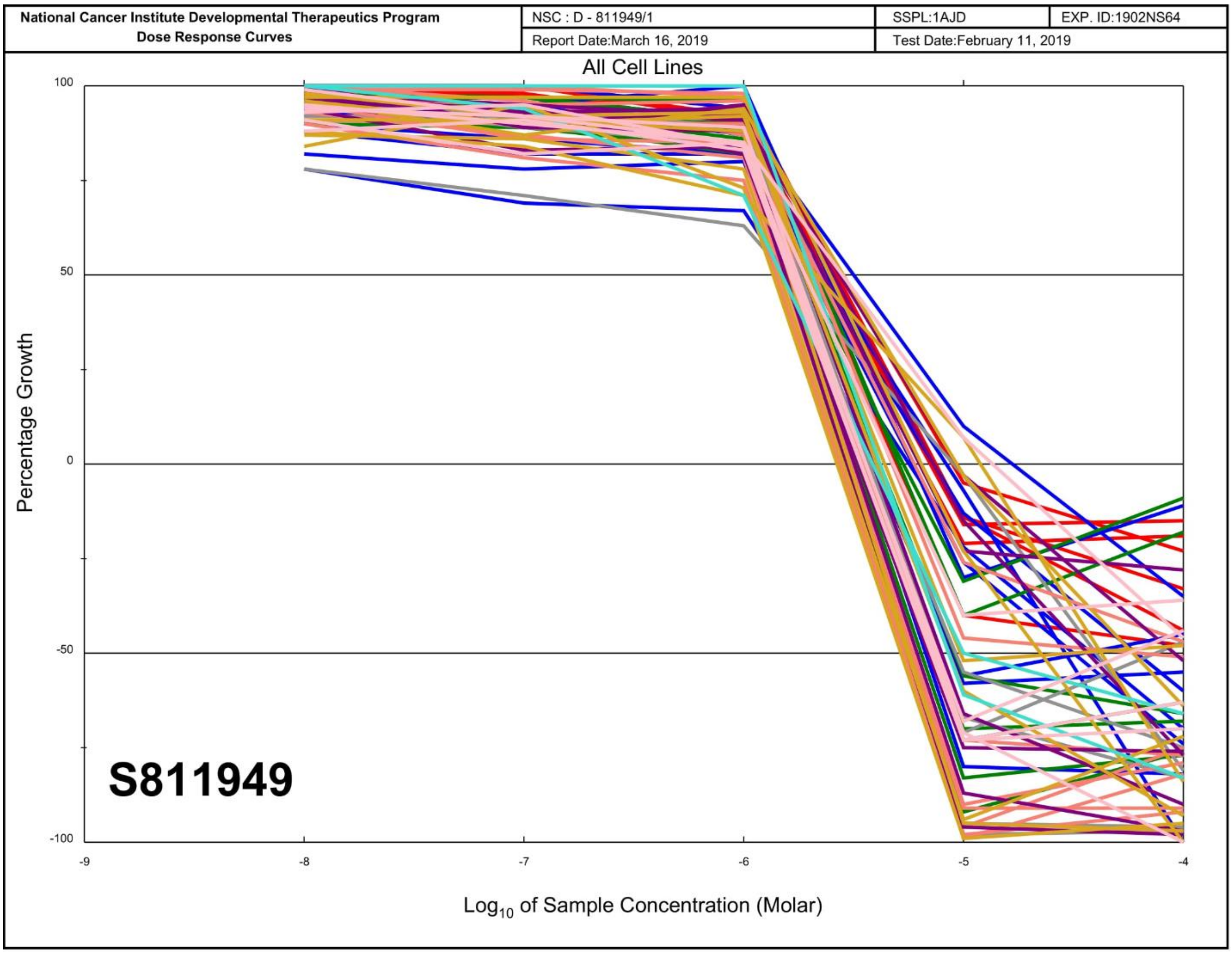


**Figure S19.** The five-dose assay data for compound **9**
